# Genomic diversity of *Helicobacter pylori* populations from different regions of the human stomach

**DOI:** 10.1080/19490976.2022.2152306

**Published:** 2022-12-05

**Authors:** Daniel James Wilkinson, Benjamin Dickins, Karen Robinson, Jody Anne Winter

**Affiliations:** a School of Veterinary Medicine and Science, University of Nottingham, Nottingham, UK; b School of Science and Technology, Nottingham Trent University, UK; c Nottingham Digestive Diseases Centre, School of Medicine, University of Nottingham, Nottingham, UK; d NIHR Nottingham Biomedical Research Centre, Nottingham University Hospitals NHS Trust, Nottingham, UK

**Keywords:** Deep sequencing, polymorphic, bacteria, adaptation, allelic diversity

## Abstract

Individuals infected with *Helicobacter pylori* harbor unique and diverse populations of quasispecies, but diversity between and within different regions of the human stomach and the process of bacterial adaptation to each location are not yet well understood. We applied whole-genome deep sequencing to characterize the within- and between-stomach region genetic diversity *of H. pylori* populations from paired antrum and corpus biopsies of 15 patients, along with single biopsies from one region of an additional 3 patients, by scanning allelic diversity. We combined population deep sequencing with more conventional sequencing of multiple *H. pylori* single colony isolates from individual biopsies to generate a unique dataset. Single colony isolates were used to validate the scanning allelic diversity pipelines. We detected extensive population allelic diversity within the different regions of each patient’s stomach. Diversity was most commonly found within non-coding, hypothetical, outer membrane, restriction modification system, virulence, lipopolysaccharide biosynthesis, efflux systems, and chemotaxis-associated genes. Antrum and corpus populations from the same patient grouped together phylogenetically, indicating that most patients were initially infected with a single strain, which then diversified. Single colonies from the antrum and corpus of the same patients grouped into distinct clades, suggesting mechanisms for within-location adaptation across multiple *H. pylori* isolates from different patients. The comparisons made available by combined sequencing and analysis of isolates and populations enabled comprehensive analysis of the genetic changes associated with *H. pylori* diversification and stomach region adaptation.

## Introduction

*Helicobacter pylori* typically first colonizes people in early childhood and then persists as a chronic, lifelong infection^1^. This usually results in gastritis, which is most often asymptomatic in nature.^2^ The chronic inflammatory nature of the infection, and the high mutation and recombination rate of this bacterium, are thought to contribute to a diverse bacterial population (quasispecies) within the infected gastric mucosa. Colonizing bacteria in the antrum and corpus regions of the human stomach will be exposed to different levels of acidity, inflammatory factors, access to sheltering glands and mucus. This variation in environmental conditions within stomachs may drive bacterial diversification over time. Particularly in high-prevalence areas, humans are colonized by multiple strains,^3^ further increasing this diversity. The high level of genetic diversity of *H. pylori* has implications for the design of successful eradication regimens and vaccines. However, the extent and characteristics of *H. pylori* diversity within infected individuals are not yet fully understood.

Genomic approaches have highlighted the intimate relationship of *H. pylori* infection and co-evolution with humans which has occurred at least since anatomically modern humans migrated out of Africa approximately 58,000 years ago.^4–6^ Genomic analyses of *H. pylori* isolates across the world have revealed a global population structure and diversity.^7–9^ Comparative genomics approaches have been taken, usually with single colony isolates from each patient, to investigate global population diversity in *H. pylori*. Other studies have characterized *H. pylori* strains from specific geographical regions.^10–13^ These global and geographical genetic analyses have facilitated the identification of genotypes associated with different disease types and severity. For example, a recent genome-wide association study^14^ (GWAS) identified SNPs and genes in *H. pylori* genomes that could be used to assess gastric cancer risk in infected people. A similar GWAS approach was undertaken to identify regions of the genome associated with the progression of duodenal ulceration to gastric cancer from East Asian (hspEAsia) *H. pylori* strains.^15^ Another study identified six genes that were associated with peptic ulcer disease and gastric adenocarcinoma, by comparing multi-ethnic populations at high to low risk of these diseases.^16^ Therefore, despite there being a very high level of diversity in *H. pylori* genomes, analysis at global and local geographical levels has provided important insights into bacterial virulence and disease progression. In addition, comparative genomic analysis of multiple single colony isolates from different regions of individual patient stomachs has informed our understanding of *H. pylori* niche adaptation and intragastric migration at the individual host level.^17^ Ailloud *et al* (2019)^17^ performed phylogenetic analyses on multiple *H. pylori* isolates from different stomach regions of 16 adult patients, and their findings suggested that pressures for genetic adaptation were different according to characteristics of the gastric niche, although there appeared to be migration of bacteria between regions of the stomach.

Studies of bacterial populations that rely on sequencing single colony isolates can only capture a subset of the genetic diversity, but bacterial diversity at the population level can be more comprehensively assessed using deep sequencing methodologies. Within-patient genetic diversity of *Burkholderia dolosa* from chronically infected individuals was previously investigated using a population deep sequencing approach,^18^ where the reads mapped onto a reference genome identified the most variable genes and regions, and captured a snapshot of within-patient genetic diversity of the whole population. For this study, we adopted a similar approach^18^ and adapted it to *H. pylori* populations from gastric biopsies taken from the antrum and corpus of infected patients. Single colonies were also isolated from a subset of these biopsies for conventional whole-genome sequencing. Here, we present a high-resolution analysis of *H. pylori* genetic structure and population diversity using these techniques in *H. pylori* populations for the first time.

## Results

To characterize *H. pylori* population diversity, a combination of deep sequencing of isolates from the antrum and corpus of human stomachs, and comparative analysis of consensus assembled genomes and multiple single colony isolates from the same populations, was applied. Gastric biopsies were obtained from the antrum and corpus of 18 infected individuals (median age 63.5 years, range 40–79 years, seven males and 11 females). *H. pylori* was cultured from the biopsies as confluent population sweeps, and this growth was streaked out for isolation of single colonies. Paired gastric isolates were recovered from the antrum and corpus of 15 patients, whereas isolates were only recovered from one site for the remaining three patients. Paired biopsies with markedly different histological inflammation and damage severity (Sydney scores), and/or differing *H. pylori vacA, cagA and cagE* virulence factor PCR genotypes, between antrum and corpus, were prioritized for inclusion in this study which aimed to utilize a combined approach of deep population and single colony isolate sequencing. The *H. pylori* population growth and 119 single colonies were sequenced using the Illumina MiSeq platform. Annotation was guided by the reference strain *H. pylori* 26695. The total data set consisted of deep sequenced populations from 33 *H. pylori* population sweeps (15 paired antral and corporal populations, and from three single location populations) and 119 genomes from single colony isolates extracted from the populations of 11 different patients

### Genome alignments revealed larger scale differences between antrum and corpus populations

**Figure 1.**
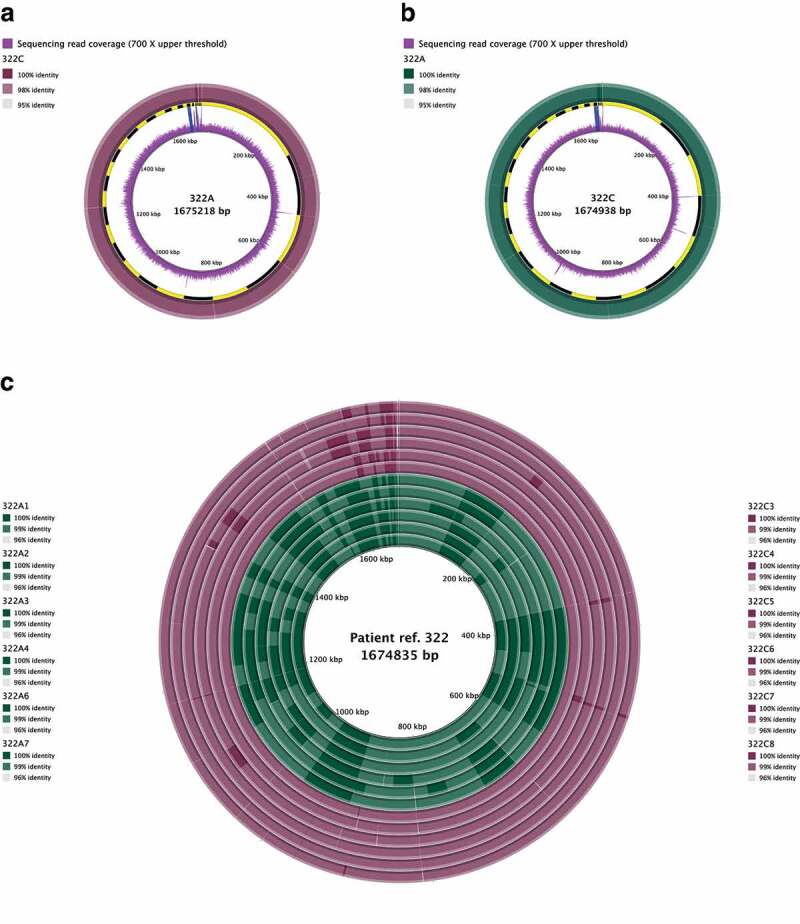
Representative whole-genome alignment for *H. pylori* strains isolated from patient 322. Whole-genome alignments of the consensus genomes generated by deep population sequencing of antrum and corpus *H. pylori* populations, using antrum (a) or corpus (b) consensus genome as the reference. Panel c depicts the alignment of colony isolate assembled genomes against the patient reference (assembly created by combining the curated reads from both antrum and corpus regions). Colour intensity of each ring indicates percentage identity between antrum and corpus consensus genomes. Positions of contigs within the assembled reference genome are shown as a ring alternating in colour between yellow and black in panels a-b to show contig boundaries. Blue bars in the coverage ring show regions >700X. Alignments for the other patients are shown in Suppl. Fig. 1-16.

Noticeable gaps or stretches with <95% BLASTN identity were identified between patients with paired antrum and corpus aligned population consensus assemblies from 12/15 patients (Fig. 1 A-B; Suppl. Fig. 1–14 A-B). There were more nonsynonymous mutations between paired antrum and corpus populations suggesting that selection pressures were acting independently within and between these regions, resulting in more nonsynonymous mutations between the populations (Suppl. Fig. 17).

For the analysis of isolates from multiple regions of the same stomach, a patient-specific combined antrum and corpus consensus assembly was *de novo* assembled using all deep population sequencing reads from the antrum and corpus datasets and used as the reference sequence (Fig. 1C; Suppl. Figure 5C, 7C, 9C, 10C, 11C, 12C, 13C, 14C). This was done to reduce the reference bias. For the two patients where paired antrum and corpus deep sequencing reads were unavailable as only one region was deep sequenced, the population consensus assembly was used as the reference to compare the single colony isolates (Suppl. Fig. 15–16).

Single colony isolate genomes from the antrum and/or corpus of 11 patients were aligned and visualized using high BLASTN identity between 96% and 100% (Fig. 1C; Suppl. Figure 5C, 7C, 9C, 10C, 11C, 12C, 13C, 14C, 15, 16) to identify small scale differences between the aligned genomes. Defined stretches of BLASTN identity were evident between single colony isolates from the antrum and corpus of each patient. This “visual fingerprint” could differentiate the isolates taken from the antrum and corpus, suggesting stomach region-specific adaptation and between-region differences.

While Figure 1 and supplementary figures 5C, 7C, 9C, 10C 11C, 12C, 13C, 14C, 15 and 16 identified differences in aligned single colony genomes from paired antrum and corpus regions, the pan-genome analysis revealed a “fingerprint” of accessory genes associated with each patient (Suppl. Fig. 18). There were very minor differences between single colony isolates from the same patient in their accessory genes including between isolates from the antrum and corpus regions. This suggests that most patients in our dataset were infected by a single strain and diversity between strains was likely due to mutations accumulating over the time of infection. The exception to this was patient 565 in which 4/6 single colony isolates showed a different accessory genome fingerprint in comparison to the other strains (2/6 from the corpus and 6/6 from the antrum region) which had the same accessory gene fingerprint. This suggests that patient 565 was likely colonized by a multi-strain infection.

### Deep sequencing and read mapping identified the most variable genes within H. pylori populations

**Figure 2.**
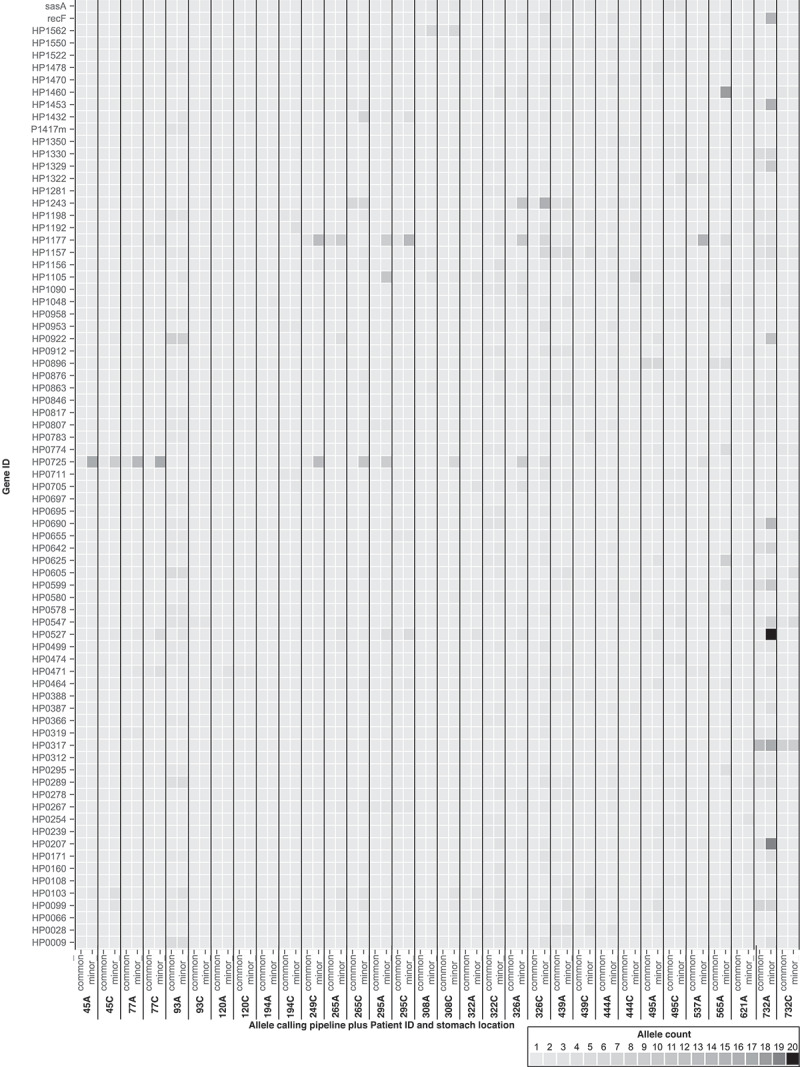
This is a filtered version of Suppl. Fig 19,20 to highlight the most diverse genes within our dataset. This heat map shows the common and minor allelic variation across genes (indicated as “HP” gene IDs in line with the nomenclature) for each antrum- and corpus-derived *H. pylori* population, derived from deep sequencing of the population from each biopsy and read-mapping back to the consensus genome to identify variant bases at each locus. Higher color intensity indicates a larger number of variant bases within that gene. Only variable genes represented by two or more patient samples were selected in the common allele variation dataset. Due to the high number of allelic genes in the minor allele variation, genes were filtered to highlight minor allelic variant genes shared between six or more different patient samples. In both allele calling datasets, sample 565C was excluded due to the extreme variation observed, likely due to this sample harboring a mixed strain infection. For an undocketed heatmap, including sample 565C, see Suppl. Fig. 19,20. A list of the gene product descriptions for each gene ID (HP number) can be found in Suppl. Table 1. A list of the minor allelic variants can be found in Suppl. Table 2.

A read mapping and polymorphic detection pipeline was developed (Table 1), to initially investigate within-region diversity using stringent thresholds to identify alleles with very high confidence, including elevated allele fractions, which were defined as ‘common alleles’ within datasets (Figure 2; Suppl. Fig. 19). Stringency thresholds were then lowered for some parameters (particularly the number of alternative reads mapping in the forward and reverse direction) to detect “minor” allele diversity (Table 1; Figure 2; Suppl. Fig. 20). “Minor” alleles were present at lower frequencies in the population, while “common” alleles were present at higher frequencies and represent polymorphisms that may be approaching fixation in the population or represent genes/bases under more recent selective pressures.^18^

**Table 1.** Description of the steps and software used in the common and minor allele calling pipelines. We adopted the workflow from Lieberman *et al* (2014)^18^ with the following format; remove adapter read-through (Trimmomatic^20^), trim low-quality bases from reads (Sickle^21^), align reads (Bowtie2^22^), call potential variants (FreeBayes^23^ and vcflib^24^). For the minor allele calling pipeline we adopted the thresholds described by Lieberman *et al* (2014)^18^ and adapted them for the ‘common allele’ calling pipeline to identify alleles that were of high confidence (additional manual trimming of SNPs and SNPs supported on both forward and reverse reads) and consisted of elevated allele fractions (15 or more alternative allele calls from reads aligning in both the forward and reverse direction).

Common allele calling pipeline	Minor allele calling pipeline
Sequencing reads were trimmed for Illumina sequencing adapters and adapter read-through using Trimmomatic.^20^ Reads were further trimmed with Sickle^21^ to Phred 30 with a minimum read length of 50 bp	Sequencing reads were trimmed for Illumina sequencing adapters and adapter read-through using Trimmomatic.^20^ Reads were further trimmed with Sickle^21^ to Phred 30 with a minimum read length of 50 bp
Bowtie2^22^ (version 2.3.4.3) used to map curated paired-end reads to the consensus assembled genome in ‘”very sensitive mode” with a maximum fragment length of 2000 bp and the number of ambiguous characters allowed between an aligned read was reduced to ~1%.	Bowtie2^22^ used to map curated paired-end reads to the consensus assembled genome in ‘”very sensitive mode” with a maximum fragment length of 2000 bp and the number of ambiguous characters allowed between an aligned read was reduced to ~1%.
SAMtools suite^25^ (version 1.9) was used to sort sequence alignment and remove PCR duplicates.	SAMtools^25^ suite was used to sort sequence alignment and remove PCR duplicates.
FreeBayes^23^ (version 1.3.1) was used with a mapping quality of Phred 34 and base quality of Phred 30.	FreeBayes^23^ was used with a mapping quality of Phred 34, base quality of Phred 30 and the minimum alternative fraction of reads to support an alternative allele was set to 3%.
Alternative alleles were called only when ≥15 supporting reads were observed in the forward and reverse direction through vcflib^24^ (version 1.0.0). Insertions and deletions were also filtered out. SNPs located in the first or last 500 bp of an assembled contig were removed manually. SNPs were manually filtered out if they were identified within a repeat region of ≥6 nucleotides where the alternative allele was >3 bases from the beginning of an individual read.	Insertions and deletions were also filtered out through vcflib.^24^ SNPs located in the first or last 500 bp of an assembled contig were removed manually.

A total of 7,920 common allelic variants were detected within 1011 genes from 33 deep-sequenced samples. Excluding hypothetical proteins, the highest common allelic variation (Figure 2; Suppl. Fig. 19) was observed within outer membrane protein (OMP) associated genes (*babA/B/C, frpB, hopA/B/C/D/E/G/H/I/L/N/Q/U/Z, hofA/B/C/D/E/F/G/H, homA/C/D, hefA/D/G, horB/C/D/E/F/G/I/J/K, lptB/D* and *sabA*) (HP gene IDs can be found in Suppl. Table 1) with polymorphisms in these genes detected in *H. pylori* populations isolated from 22/33 patient biopsies. Polymorphisms in virulence-related genes including the *vacA* paralogue HP0289 (n = 5), *babA* (n = 5), *cagA* (n = 4) and *sabA* (n = 4) were also identified in the common allele dataset. Other notable genes with high allelic diversity within the common allele dataset were those encoding the glutathione-regulated potassium-efflux system protein HP0471 (n = 5), DNA-directed RNA polymerase subunit beta/beta (HP1198; n = 5) and acetyl-CoA acetyltransferase (HP0690; n = 5).

There were 16,492 minor allelic variants within 1,738 genes from 33 deep-sequenced samples (Suppl. Table 2). As with the common allelic variant analysis, OMP genes (in addition to *hopF/H/K, hefB/C, horA/H/I/L* and *lptA*) (Figure 2; Suppl. Fig. 20) (HP gene IDs can be found in Suppl. Table 1) had the highest number of variants in the minor allele dataset (n = 27). Methyl-accepting chemotaxis genes (*tlpA*/*B*/*C* and HP0599; n = 18), restriction modification genes (*hsdM/R, hdsM/R, hpy8I, mboIIR*, HP0479, HP0592, HP1351, HP1367, HP1368, HP1371, HP1471, HP1472, HP1499, HP1517, HP1521, HP1522; n = 19), *vacA* like genes (*imaA, vlpC*, HP0610; n = 14), *cag*PAI genes (cagΔ/β/γ/A/C/D/E/F/H/M/N/S/W/X/Y/Z; n = 18), sialic acid-binding adhesin gene *sabA* (n = 11), lipopolysaccharide biosynthesis genes (HP0208; HP0208; n = 13) and the glutathione-regulated potassium-efflux system (HP0471; n = 11) were also highly variable (Figure 2; Suppl. Fig. 20).

The length of *H. pylori* colonization time (years) was estimated for each patient and stomach location using the deep population sequencing data and the mutation rate determined by Kennemann *et al* (2011)^26^ and denoted in Suppl. Table 3. Briefly, the number of observed minor allelic variant positions for each dataset were divided by the number of mutations per site per year (41.7 mutations per year in a genome size ~ 1.63254 Mb). Excluding the patient in which a mixed infection was suspected (patient 565), the average length of infection of *H. pylori* from a snapshot in time, was calculated to be ~3.1 years (range 0.07–21.7 years; Suppl. Table 3).

### Combining population deep sequencing, read mapping and genome alignment allows more powerful analysis of H. pylori population structure and diversity

**Figure 3.**
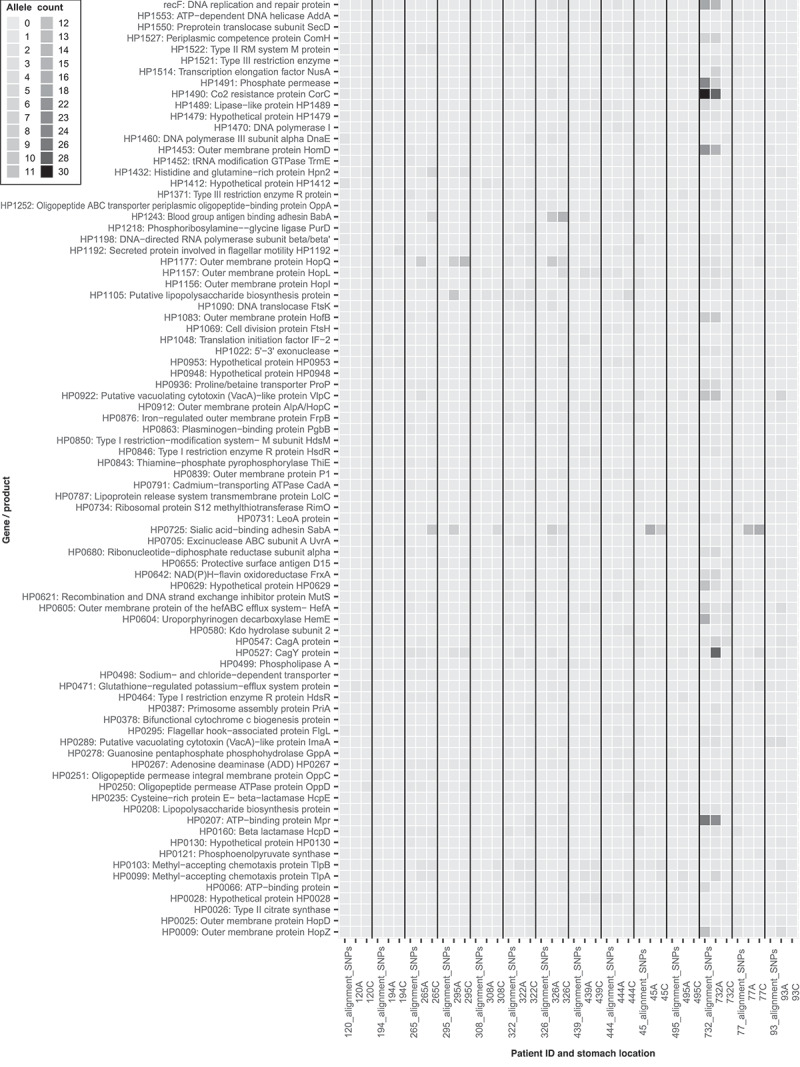
This is a filtered version of Suppl. Fig 21 to highlight the most diverse genes within our dataset. This is a heat map showing the most variable genes and populations identified by a combination of analytical approaches (within and between bacterial populations from the antrum and corpus regions of patient stomachs). Each column contains data from one patient. Within each column, three datasets are shown. From left to right these are: between stomach region variation from whole-genome alignment antrum versus corpus; within antrum minor allele variation; within corpus minor allele variation. Darker color intensity indicates a larger number of variant bases within that gene/gene product. Gene products/genes that were shown to be diverse six or more times by any methodology across the patient datasets were included in this figure to reduce figure size and to highlight genes/gene products with higher observed genetic diversity. Hypothetical proteins and intergenic nucleotide diversity were removed from this dataset to improve visualization. Only patients with paired antrum and corpus data were included. Patient 565 was excluded due to the extreme variation observed, likely due to this sample harboring a mixed strain infection. A undocketed figure including patient 565 can be found in Suppl. Fig. 21. This Figure combines the information presented in Figure 2 plus Suppl. Fig. 20 (detection of minor allelic variants within each stomach region by read-mapping of deep sequencing reads back to the consensus genome) and the methodology described in Suppl. Fig 23 (detection of validated SNPs by whole-genome alignment between stomach regions).

Heat maps were produced (Figure 3; Supp. Fig. 21) to combine the key findings from the population consensus whole-genome alignments (Fig. 1A-B; Suppl. Fig. 1–14 A-B) and read mapping (minor allele calling pipeline) approach (Figure 2; Suppl. Fig. 20) and these highlighted the number of differences detected by each approach. Some genes, for example *cagY* (HP0527) in patient 265, were identified as having differences between the antrum and corpus regions by genome alignment but also had polymorphic diversity *within* one or both region(s) detected by the minor allele calling pipeline. Other genes were different between the regions (by consensus genome alignment) but showed no within niche diversity at either region e.g. *dnaE* (HP1460) in patient 120. This shows how both techniques can be used in tandem to better capture the genetic diversity both within and between populations of *H. pylori*.

### Single colony sequencing added value by allowing phylogenetic and pan-genome analyses

**Figure 4.**
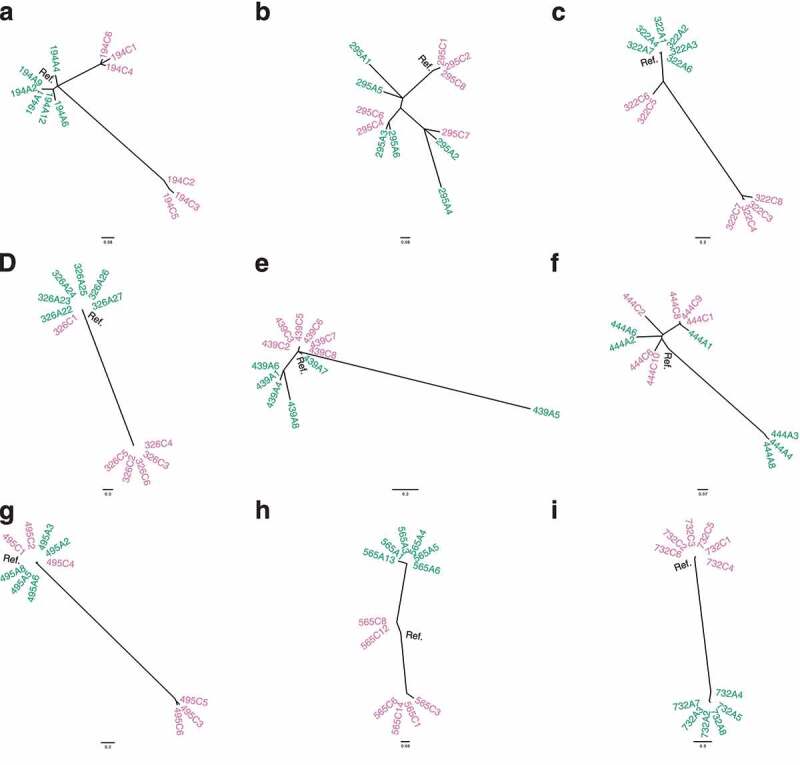
Phylogenetic trees depicting single colony isolates from patients with paired antrum and corpus isolates: a) 194, b) 295, c) 322, d) 326, e) 439, f) 444, g) 495, h) 565 and i) 732. Antrum-derived strains are shown in green and corpus-derived strains in reddish purple. The patient reference consensus genome is shown in black. Phylogenies were rooted to the midpoint in decreasing order and drawn in a radial format.

Phylogenetic trees were constructed for the single colony isolates from patients with isolates from paired antrum and corpus regions (Figure 4). While the antrum and corpus isolates from some patients separated out into different clades, this was not always the case (e.g. Figure 4b, 4f). There was evidence of migration of *H. pylori* between antrum and corpus in 5/9 patients with one or more isolate(s) clustering in the opposite region’s isolate cluster. Pan-genome analysis confirmed that the isolates from each patient clustered together (Suppl. Fig. 18), indicating that most patients had originally been infected with a single strain that subsequently diversified. The exception was patient 565 in whom two distinct *H. pylori* clades were evident suggesting a mixed strain infection.

### Population deep sequencing with read mapping is much more sensitive for detecting polymorphisms than conventional single colony sequencing, but combining both methods is best

Single colony isolates were used to validate the deep sequencing minor allele calling pipeline. Using a read mapping approach and exact base matching to compare methodologies (minor allele calling of deep sequenced populations and SNP calling between single colony isolates) we determined an average of 68.69% (95% CL: 59.13–78.25%) alleles were detected only by the population deep sequencing, 8.13% (95% CL: 4.83–11.43%) by the single colony isolate analysis and 23.18% (95% CL: 14.93–31.43%) were concordant between methodologies (Suppl. Fig. 22).

## Discussion

By employing a combination of deep and single colony sequencing approaches in *H. pylori* for the first time, we detected extensive diversity within and between bacterial populations from the antrum and corpus regions of patient stomachs, including in virulence and colonization-associated genes. This combined approach generated a richer dataset than either individual approach.

Of the studies that have investigated genetic diversity of *H. pylori* within individual patients,^17,27–36^ only four^17,27,34,36^ used a whole-genome sequencing approach. Others used PCR amplification and sequencing to focus on specific genes and loci. Didelot *et al* (2013)^27^ sequenced single isolates from the antrum and corpus of 45 individuals and found evidence of micro-evolution of the bacterial population within each individual’s stomach.

In this study, we used deep sequencing to detect much higher levels of genetic diversity in *H. pylori* populations than has previously been reported, even when very stringent parameters were applied (Table 1; Figure 2). When the parameters were relaxed to detect minor allelic variants (Table 1; Figure 2), a much larger number of polymorphic sites were detected. This study presents a comprehensive snapshot of *H. pylori* genetic diversity at a single point in time.

Some of the most frequently identified common allelic variant genes were related to virulence and colonization, e.g. OMPs, *vacA* paralogue HP0289, *babA* (HP1243), and *cagA* (HP0547). In comparison, many of the minor allelic variants were in OMP genes which make up approximately 4% of the *H. pylori* coding genome^37^ and are highly diverse and polymorphic.^37–39^ Most studies to date have studied polymorphic variation in *H. pylori* OMPs between patients, geographical regions, sequential isolates from animal models or familial isolated strains to reach these conclusions.^27,38,40,41^ The comparative genetics approaches of such studies have contributed to the identification and understanding of OMP polymorphic diversity, but polymorphic diversity has rarely been identified^36^ within populations taken from the same time point, despite the identification of OMP phase variation and gene conversion.^40–42^ This is perhaps hampered by the difficulty and increased workload in isolating single colonies from population sweeps, the lack of paired biopsy samples from the same patient and the increased sequencing costs such investigations incur.

*cagA* gene diversity between individuals and geographical regions^8,43^ is well characterized. In this study, we evidence both within- and between-stomach region genetic diversity of *cagA* (HP0547; Figure 2; Figure 3; Suppl. Fig. 19–21) which supports the observations of other studies.^17,36^ Within- and between-region diversity in *cagA* sequences could result in variations in virulence activity within the stomach, thus influencing gastritis patterns and disease development. Within-region polymorphic diversity of *vacA* paralogues HP0289, HP0610 and HP0922 was also observed. These genes are thought to play roles in host colonization and collagen degradation. Diversity here could therefore impact on colonization, persistence, and disease outcomes.

Several important genes and groups of genes were identified within both the common and minor allele pipelines (Figure 2; Suppl. Fig. 19–20). These included the DNA-directed RNA polymerase beta subunit (HP1198/*rpoBC*). Certain mutations within the *rpoB* gene of *H. pylori* have been shown to increase resistance to rifamycins.^44,45^ We also observed diversity in the clarithromycin resistance-associated gene *kefB* (HP0471).^46,47^ These observations might help to explain eradication therapy failure within patients whereby minority-resistant strains persist within the population. Methyl-accepting chemotaxis associated genes (HP0082; HP0103; HP0099; HP0599) were also identified (including population consensus genome alignment-based SNPs in patient 45). These genes are important for *H. pylori* colonization and survival, as *tlpA* (HP0099) senses arginine, bicarbonate, and acidic pH,^48,49^ and *tlpB* (HP0103) is essential for chemotaxis away from acidic pH and toward more favorable conditions.^50^ Such allelic diversity could affect the sensitivity of chemotactic responses and enable colonization of different areas of the stomach by these strains.

In addition, restriction-modification system genes were identified as highly allelic. Furuta *et al* (2015),^51^ observed similar genetic diversity among restriction-modification associated genes using a comparative genetics approach with single colony isolates obtained from five families. Other studies have also observed restriction-modification system diversity between strains taken from different patients.^11,52,53^ Bacterial restriction-modification systems confer protection against invading foreign DNA^54^ but do not pose a barrier to homologous recombination.^55^ Restriction-modification systems can also influence gene expression^56,57^ and may have roles in adhesion and virulence.^58,59^ Diversity in restriction-modification genes could therefore play a role in niche adaptation and persistence.

Lipopolysaccharide biosynthesis associated genes^60^ were also highly polymorphic in the common and minor allele pipelines (Figure 2; Suppl. Fig. 19–20). 18 samples had within-region minor allelic diversity amongst genes of the putative outer membrane biogenesis complex components.^60^ While we have focused on differences and diversity, our dataset could equally be used to identify useful regions of conservation that might be exploited in vaccine development. For example, genetic interruption of the HP0270 gene in *H. pylori*, a homolog of the LpxM protein involved in lipid A biosynthesis, has been shown to be lethal.^61^ Our data suggest that HP0270 is conserved as no allelic diversity was observed within any samples in our minor allele calling pipeline. Other such examples are likely to be extracted from this dataset which might be useful for vaccine design.

Estimation of the length of time *H. pylori* populations had been colonizing at the time of sampling (Suppl. Table 3) was likely to be an underestimate due to the stringency of the minor allele calling pipeline (Table 1). This was backed up by the single colony isolate SNP analysis (Suppl. Fig. 22) which identified 8.1% of SNPs not called by the minor allele pipeline. Furthermore, selection pressures acting on the populations over time are likely to add to this underestimation. Our dataset suggested that the average population showed a signature of mutations consistent with a diversification over 3.1 years (range 0.07–21.7 years; Suppl. Table 3). This is consistent with many other studies.^17,26,27^ However, some populations, such as the antrum and corpus locations of patients 565 and 732, estimated a length of infection as long as 289 years. It is speculated that these patients were likely infected with a mixed strain infection which has diversified over time. In these cases, the snapshot of the length of diversification is likely to be an overestimation due to homologous recombination acting between these different strains.^27^ These observations might have been overlooked if only a single colony sequencing approach was used as deep sequencing was able to snapshot an abundance of allelic diversity within the populations.

Our combined approach revealed that some genes identified with *between*-region diversity by genome alignment also had polymorphic diversity *within* one or both regions of the stomach (Figure 3; Suppl. Fig. 21). For example, the iron regulated OMP gene (HP0876) of sample 265C was polymorphic within this population but was also identified by whole-genome alignment as variable between the consensus genome sequences of the antrum and corpus-derived samples from patient 265. This observation was not uncommon across the dataset.

The phylogenetic relationships between single colony isolates obtained from the antrum and corpus in this study (Figure 4) agree with Ailloud *et al* (2019).^17^ There were distinct clades of *H. pylori* between the antrum and corpus in some patients, and there was evidence of migration between the two sites in others. Ailloud *et al* (2019)^17^ showed that migration of *H. pylori* strains from the antrum to the corpus was relatively infrequent, whereas migration between the corpus and fundus is a more common event, perhaps due to the more significant environmental differences between the antrum and corpus regions. Our phylogenetic analysis also indicated that where an antrum cluster was present, corpus strains were more frequently observed within antrum clades than vice versa. This suggests that while migrations between the antrum and corpus are infrequent, the corpus isolates are more likely to migrate to the antrum than vice versa. Again, this could be due to the differences between the antrum and corpus environments where antrum isolates are less fit or poorly adapted to colonize the harsher oxyntic epithelium whereas the corpus strains are able to colonize the more neutral antrum glands.

An alternative explanation for the non-clustering of antrum and corpus strains in patients 295 and 444 could be due to the biopsy sampling location and method. Fung *et al* (2019),^62^ showed how founder strains initially colonize glands then spread to adjacent glands in the immediate vicinity. This creates islands of closely related *H. pylori* strains, and where island boundaries occur the inhabitants may then compete for space. At these boundaries there are glands containing a mixture of strains, or adjacent glands containing different strains side by side. Transition zones between the antrum and corpus typically contain mixed populations. The size of a biopsy (how many strain islands it spans) and its proximity to the antrum-corpus transition zone may influence the *H. pylori* diversity observed. This could potentially disrupt the genetic clustering and phylogenetic topology of the antrum and corpus isolates. Therefore, standardized sampling locations within the antrum and corpus would be beneficial for this type of analysis. However, this may not be feasible in practice because biopsies are often taken from areas likely to harbor *H. pylori* infection, such as adjacent to visually diseased epithelium.

The pan-genome analyses of the single colony isolates and the consensus assembled deep-sequenced populations, both showed that the strains from each patient had a unique pattern of gene presence and absences, distinct from the patterns observed in strains from other patients (Suppl. Fig. 18). The accessory genome “fingerprint” identified between patients appeared to be largely core genes between strains taken from the same stomach, regardless of the stomach location. However, while the *H. pylori* strains from patient 565 all clustered together, there was substantial gene content differences between single colony isolates from this patient. The corpus isolates from this patient were much more diverse than the antrum isolates, not just allelically but also in their accessory genes. Consequently, patient 565 may have been infected by more than one *H. pylori* strain that exchanged DNA through homologous recombination and/or natural transformation over time and now share a similar genetic makeup but the population is much more genetically diverse in comparison to a single strain infection.

By combining conventional sequencing of single colony isolates from the antrum and corpus with population deep sequencing of the same samples, from multiple patients, we were able to comprehensively characterize *H. pylori* population diversity. The dual approach allowed for comparative analysis to determine how well the data generated from each approach agreed with each other. This confirmed that the deep sequencing allelic calling pipeline was able to detect 91.87% of the SNPs, with only 8.13% of SNPs identified from the single colony pipeline alone (Suppl. Fig. 22). The population deep sequencing minor allele calling methodology captured a more comprehensive snapshot of population genetic diversity compared to the single colony approach. However, the minor allele calling pipeline is still likely to be an underestimate of the true diversity present, due to the stringent quality control parameters that were applied, and single colony isolate sequencing from the same populations added value because it enabled additional phylogenetic and recombination analyses that would not have been possible using the deep sequencing data alone.

Combining the two complementary approaches of deep population sequencing and single colony isolate sequencing generated more information than either individual strategy. But this combined approach was intensive and was only applied to a relatively small number of UK-based patients in this study. This study did not have sufficient sample size to identify any significant associations between genomic traits in the *H. pylori* populations and the presence of ulcers, intestinal metaplasia, or severe inflammation in the patients’ stomachs. This study also relied on a culture-based approach, so may not provide a full picture of the genetic diversity of strains present in the stomach due to the selection pressures applied by culture on agar plates prior to sequencing. Although genetic changes over time can be inferred from the data, this study was not longitudinal.

In conclusion, we have shown that single colony analysis alone can identify fixed differences in the genomes of *H. pylori* between stomach regions, but population deep sequencing reveals that underlying variation is still present and the population as a whole retains a high degree of potential plasticity. This may help explain why *H. pylori* can persist in a chronic infection. Identifying loci with minimal variation might usefully inform future vaccine design, while loci in which high numbers of minor allelic variants are concentrated indicate which genes are critical for niche adaptation and persistence.

## Materials and methods

**Figure 5.**
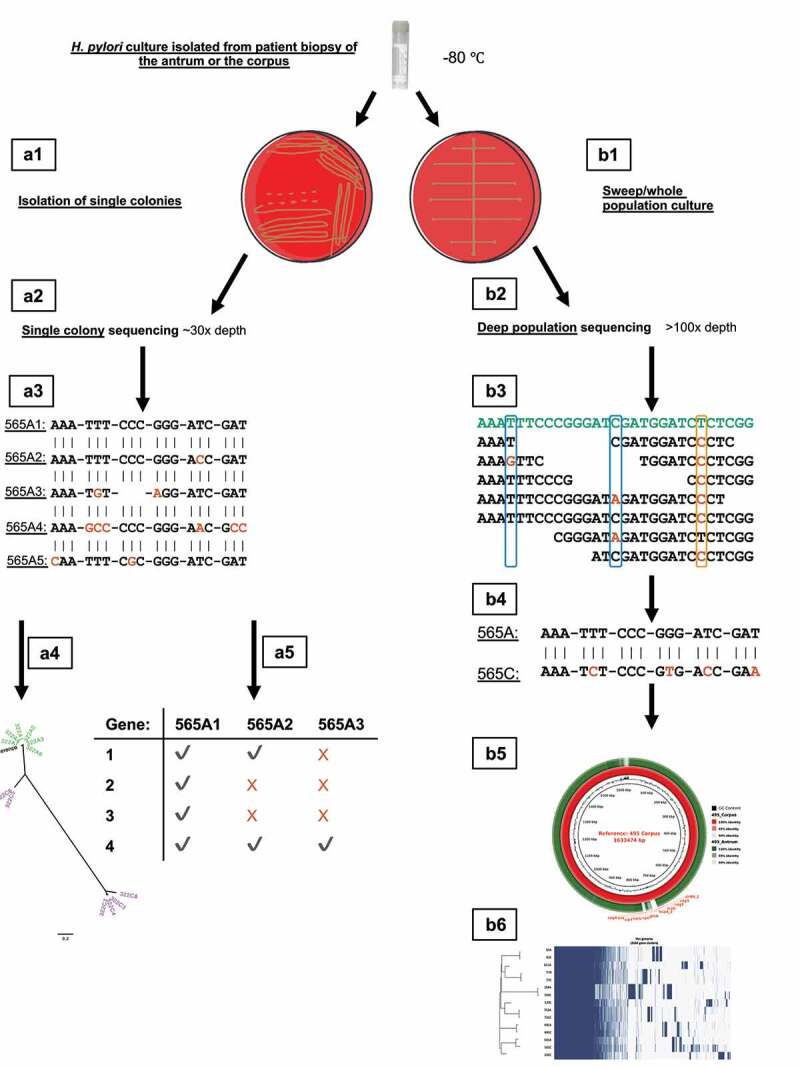
Overview of study design. *H. pylori* isolates from corpus and antrum biopsies were subjected to population deep sequencing (right; b1-6) and streaked out to single colonies which were individually sequenced (left; a1-5). Data from the two approaches were used to analyze *H. pylori* diversity within and between stomach regions and individuals. a1 – isolation of single colony isolates; a2 – single colony isolates sequenced to ~30x depth; a3 – single colony isolate genomes aligned to investigate between strain diversity with orange nucleotide bases representing SNPs; a4 – single colony isolate genomes used to create phylogenetic trees; a5 – gene presence and absence analysis. b1 – culture of *H. pylori* populations from each biopsy; b2 – population deep sequencing to >100x depth; b3 – within population diversity investigated by mapping reads to the consensus genome (green colored nucleotides) with minor (blue boxes) and common (orange box) allelic diversity highlighted; b4 – between stomach region diversity analyzed by aligning the consensus genome assemblies from each regions’ population; b5 – BLASTN identity between stomach region populations investigated using the consensus assembled population genomes, highlighting areas of high diversity using BRIG;^19^ b6 – pan-genome analysis.

A summary of the methods used in this study is presented in Figure 5.

### Bacterial culture

*H. pylori* was grown on blood agar base 2 with 5% horse blood (Oxoid, Basingstoke, UK) for 24–48 h at 37°C under humid, microaerobic conditions. Cultures were stored in iso-sensitest medium containing 15% (v/v) glycerol (Sigma Aldrich, UK) at −80°C.

### Clinical samples

Gastric biopsies were donated by 18 *H. pylori*-infected patients (median age 63.5 years, range 40–79 years; 38.9% male) attending the Queen’s Medical Center, Nottingham, UK, for routine upper GI tract endoscopy to investigate dyspeptic symptoms. Written informed consent was obtained, and the study was approved by the Nottingham Research Ethics Committee 2 (08/H0408/195). Patients were excluded if taking antibiotics, proton pump inhibitors or >150 mg/day aspirin, 2 weeks preceding the endoscopy. Sections cut from fixed, paraffin embedded biopsies, were stained with hematoxylin and eosin and assessed by an experienced histopathologist who was blinded to all other data. Modified Sydney scores for inflammation, activity, atrophy, and intestinal metaplasia were provided.^63^ Isolates underwent PCR genotyping for the virulence genes *vacA, cagA* and *cagE* as described previously.^64,65^ Patients with variable Sydney scores and/or *H. pylori* virulence genotypes between their antrum and corpus biopsies were prioritized for inclusion in this study. Biopsies taken from the antrum and corpus of 15 patients, along with single biopsies from one region of 3 patients were swept across blood base #2 agar plates containing 5% (v/v) horse blood and incubated for 2–3 days at 37°C under microaerophilic conditions (10% CO_2_, 5% O_2_, 85% N_2_). *H. pylori* growth from each biopsy sweep was picked and pooled into isosensitest broth containing 15% (v/v) glycerol for long-term storage at −80°C. For single colony isolation, pooled cultures were streaked out to obtain multiple single colony isolates. These were picked at random and passaged one to two times to increase bacterial numbers for long-term storage.

### DNA extraction and whole-genome sequencing

Genomic DNA from population sweeps and single colony isolates was extracted using the QIAGEN QIAmp DNA Mini Kit following manufacturer’s instructions. DNA quality was determined using the NanoDrop2000 spectrophotometer using strict absorbance ratio cutoff values of A260/A280 1.8–1.9 and A260/A230 1.9–2.2. Genomic DNA was quantified using the dsDNA high sensitivity assay on a Qubit v4.0 fluorometer and diluted to 0.3 ng/μl for creation of Nextera XT paired-end libraries. Sequencing used Illumina V3 chemistry cartridges spiked with 1% PhiX DNA run at 2 × 250 cycles on the MiSeq platform.

The raw sequencing data generated in this study were deposited on the NCBI website with the accession code PRJNA787419.

### Sequencing read curation, contamination detection, whole-genome assembly, and annotation

Sequencing reads were trimmed for Illumina sequencing adapters and adapter read-through using Trimmomatic^20^ (version 0.38). Reads were further trimmed with Sickle^21^ (version 1.33) to Phred 30 with a minimum read length of 50bp. Trimmed reads were inspected by FastQC^66^ 0.11.7 for confirmation of expected trimming. Curated reads were passed through Kraken^67^ 1.0 and the MiniKraken database (RefSeq complete bacterial, archaeal and viral genomes captured on 18/10/2017) to detect contaminated samples, defined as <92% of curated sequencing reads mapped to *H. pylori* and/or <95% of the reads mapped to the *Helicobacter* genus. Two deep-sequenced samples (308A and 326A) contained contaminant reads from an unknown species.

Curated reads were used to construct *de novo* assemblies using the SPAdes^68^ 3.11.1 assembler in careful mode creating consensus whole-genome assemblies for the deep-sequenced clinical sweep samples and contigs <500 bp were removed. For analysis of strains from multiple regions of the same stomach (Fig. 1C; Suppl. Figure 5C, 7C, 9C, 10C, 11C, 12C, 13C, 14C, 15, 16), a patient-specific combined antrum and corpus consensus assembly was *de novo* assembled using the same settings by combining the curated reads from both antrum and corpus regions. This was done to reduce the reference bias associated with using either the consensus assembly from the deep sequenced antrum or corpus populations. For strains isolated from patients where only one region was population deep sequenced, the population consensus assembled genome sequence from that region was used as the reference genome. Sequencing depth/coverage was determined using mosdepth 0.2.3.^69^ Contaminant reads were assembled into separate contigs and excluded from all further analyses with the exception of BLAST ring image generator (BRIG; version 0.95) analysis^19^ in Suppl. Fig 8–9.

Consensus population sweeps and single colony assemblies were annotated using Prokka^70^ (version 1.13) guided by the reference *H. pylori* strain 26695 (NC_000915.1) with an e-value threshold of 0.001 to account for gene diversity. A list of the gene product descriptions for each gene ID (HP numbers) can be found in Suppl. Table 1.

### Within-region diversity of deep-sequenced bacterial sweeps

A read mapping and polymorphic detection pipeline was developed similar to that of Lieberman *et al* (2014)^18^ using identical thresholds for minor allele calling and adapted thresholds to identify alleles with very high confidence thresholds including elevated allele fractions, which were defined as ‘common alleles’ within datasets. An overview of the pipeline developed for this study is denoted in Table 1.

Validation of the minor allele calling pipeline was achieved by mapping the curated single colony sequencing reads of the antrum and the corpus to the corresponding clinical sweep deep-sequenced consensus assembled reference genomes from each region (19 regions total) using Snippy^71^ 4.4.0 with an alternative allele support >90%, coverage of ≥6, minimum mapping quality of Phred 34 and minimum base quality of Phred 30. Data from 565C were excluded due to the potential presence of different *H. pylori* strains, 565C1 and 565C6, which had an unusually high number of contigs (565C1 – 1,501; 565C6 – 1,295), low N50 statistics (565C1 – 3,665; 565C6 – 3,977) and large genome lengths (565C1 – ~2.62 Mbp; 565C6 – ~2.58 Mbp).

Within-region diversity was further investigated using the single colony isolates from each patient and analyzed through the BRIG^19^ (Fig. 1C; Suppl. Figure 5C, 7C, 9C, 10C, 11C, 12C, 13C, 14C, 15, 16). Briefly, the single colony isolate genomes from both the antrum and/or corpus of each patient were aligned^72^ to the patient reference consensus genome which was assembled as previously described using all deep population sequencing reads from the antrum and corpus datasets (population consensus reference genome if only one location was sequenced) and run through BRIG with a lower identity threshold of 96%. Where there were gaps in the query genomes, the genes were annotated and overlaid to highlight the missing genes.

Length of *H. pylori* colonization time (years) was estimated for each patient and stomach location using the deep population sequencing data and the mutation rate determined by Kennemann *et al* (2011)^26^ and denoted in Suppl. Table 3. Briefly, the number of observed minor allelic variant positions for each dataset were divided by the number of mutations per site per year (41.7 mutations per year in a genome size ~ 1.63254 Mb). The following equation was used where X is the total number of minor allelic sites identified by the minor allele calling pipeline for each population:

$$Estimated\;colonisation\left(years\right)=\frac{\overset{X}{\left(total\;number\;of\;minor\;allelic\;variant\;sites\right)}}{\overset{41.7}{\left(expected\;number\;of\;mutations\;per\;site\;per\;year\right)}}$$

### Between-region diversity

BRIG was also used to investigate diversity between stomach regions. Paired antrum and corpus consensus assembled genomes from the deep population sequencing were used, and BLASTN identity was set to 95% to account for more potential diversity in this dataset. The antrum and corpus consensus genomes for each patient were used as the reference sequence, with the alternative region used as the query sequence in order to fully investigate between-region genomic diversity. Where there were gaps in the query genomes, the genes were annotated and overlaid to highlight the missing genes.

Mauve^72^ was used to align paired antrum and corpus region population consensus genomes for each patient and the alignment SNPs between the aligned genomes were exported.

Between-region diversity was further investigated using a consensus and read mapping alignment approach depicted in Suppl. Fig. 23 and was used to identify alignment SNPs of high confidence by comparison to the alignment SNPs identified by the Mauve alignment. These curated, between region population consensus genome alignment SNPs were used to compare against the minor allele calling pipeline results displayed in Figure 3.

High confidence alignment SNPs identified by consensus genome alignment and read mapping (Figure 3; Suppl. Fig. 23) were passed through a custom build of SnpEff^73^ 4.3 to determine whether alignment identified SNPs between paired antrum and corpus region population consensus genomes were synonymous or nonsynonymous (Suppl. Fig. 17).

Core genome phylogenetic trees were constructed from patients with paired antrum and corpus single colony isolates through Snippy^71^ 4.4.0 and sites of recombination were detected and removed via Gubbins 2.3.1. Polymorphic sites between the aligned genomes were extracted to create a SNP alignment file using the SNP-sites tool^74^ 2.4.1. The SNP alignment-based phylogeny was constructed using FastTree^75^ 2.1.1 which infers approximately maximum-likelihood trees.

Pan-genome analysis of all single colony isolates was undertaken by Panaroo^76^ version 1.2.2 using default settings (Suppl. Fig. 18).

### Heat maps and Venn diagrams

Heat maps were created using ggplot2 and RColorBrewer through R statistical software^77^ 3.5.1. “HP” gene reference numbers (Suppl. Table 1) from the reference strain 26695 (NC_000915.1) were used for nomenclature followed by the gene product information as provided by the genome annotation pipeline described above. Where there were no matches to “HP” numbers, the gene abbreviation (if applicable) was used followed by the gene product information.

Venn diagrams were created using the R statistical software VennDiagram package 1.6.20.^78^ Where deep-sequenced within population allelic sites matched exactly with SNPs identified between single colony isolates and the consensus assembled genome for each patient and stomach location, the overlapping and unique SNP and allelic sites were determined. This data was used as an internal control to identify the overlap between the single colony and deep sequencing minor allele calling methodologies. We show good concordance between the two methodologies suggesting that the minor allele calling methodology has power to identify true positive variant sites within the populations.

## Supplementary Material

Supplemental Material

## Data Availability

The data that support the findings of this study are openly available on the NCBI website at https://www.ncbi.nlm.nih.gov/bioproject/PRJNA787419 with the accession code PRJNA787419.

## References

[1] Miyaji H, Azuma T, Ito S, Abe Y, Gejyo F, Hashimoto N, Sugimoto H, Suto H, Ito Y, Yamazaki Y, et al. *Helicobacter pylori* infection occurs via close contact with infected individuals in early childhood. J Gastroenterol Hepatol. 2000;15(3):257–19. doi:10.1046/j.1440-1746.2000.02070.x. 10764025

[2] Watari J, Chen N, Amenta PS, Fukui H, Oshima T, Tomita T, Miwa H, Lim K-J, Das KM. *Helicobacter pylori* associated chronic gastritis, clinical syndromes, precancerous lesions, and pathogenesis of gastric cancer development. World J Gastroenterol. 2014;20(18):5461–5473. doi:10.3748/wjg.v20.i18.5461. 24833876 PMC4017061

[3] Jorgensen M, Daskalopoulos G, Warburton V, Mitchell HM, Hazell SL. Multiple strain colonization and metronidazole resistance in *Helicobacter pylori*-infected patients: identification from sequential and multiple biopsy specimens. J Infect Dis. 1996;174(3):631–635. doi:10.1093/infdis/174.3.631. 8769626

[4] Falush D, Wirth T, Linz B, Pritchard JK, Stephens M, Kidd M, Blaser MJ, Graham DY, Vacher S, Perez-Perez GI, et al. Traces of human migrations in *Helicobacter pylori* populations. Science. 2003;299(5612):1582–1585. doi:10.1126/science.1080857. 12624269

[5] Moodley Y, Linz B, Bond RP, Nieuwoudt M, Soodyall H, Schlebusch CM, Bernhöft S, Hale J, Suerbaum S, Mugisha L, et al. Age of the association between *Helicobacter pylori* and man. PLoS Pathog. 2012;8(5):e1002693. doi:10.1371/journal.ppat.1002693. 22589724 PMC3349757

[6] Linz B, Balloux F, Moodley Y, Manica A, Liu H, Roumagnac P, Falush D, Stamer C, Prugnolle F, van der Merwe SW, et al. An African origin for the intimate association between humans and *Helicobacter pylori*. Nature. 2007;445(7130):915–918. doi:10.1038/nature05562.An. 17287725 PMC1847463

[7] Duncan SS, Valk PL, Shaffer CL, Bordenstein SR, Cover TL. J-Western forms of *Helicobacter pylori cagA* constitute a distinct phylogenetic group with a widespread geographic distribution. J Bacteriol. 2012;194(6):1593–1604. doi:10.1128/JB.06340-11. 22247512 PMC3294863

[8] Olbermann P, Josenhans C, Moodley Y, Uhr M, Stamer C, Vauterin M, Suerbaum S, Achtman M, Linz B. A global overview of the genetic and functional diversity in the *Helicobacter pylori cag* pathogenicity island. PLoS Genet. 2010;6(8):e1001069. doi:10.1371/journal.pgen.1001069. 20808891 PMC2924317

[9] Montano V, Didelot X, Foll M, Linz B, Reinhardt R, Suerbaum S, Moodley Y, Jensen JD. Worldwide population structure, long-term demography, and local adaptation of *Helicobacter pylori*. Genetics. 2015;200(3):947–963. doi:10.1534/genetics.115.176404. 25995212 PMC4512554

[10] Cortes MCC, Yamakawa A, Casingal CR, Fajardo LSN, Juan MLG, De Guzman BB, Bondoc EM, Mahachai V, Yamazaki Y, Yoshida M, et al. Diversity of the *cagA* gene of *Helicobacter pylori* strains from patients with gastroduodenal diseases in the Philippines. FEMS Immunol Med Microbiol. 2010;60(1):90–97. doi:10.1111/j.1574-695X.2010.00722.x. 20670275

[11] Kojima KK, Furuta Y, Yahara K, Fukuyo M, Shiwa Y, Nishiumi S, Yoshida M, Azuma T, Yoshikawa H, Kobayashi I. Population evolution of *Helicobacter pylori* through diversification in DNA methylation and interstrain sequence homogenization. Mol Biol Evol. 2016;33(11):2848–2859. doi:10.1093/molbev/msw162. 27604221

[12] Latifi-Navid S, Ghorashi SA, Siavoshi F, Linz B, Massarrat S, Khegay T, Salmanian A-H, Shayesteh AA, Masoodi M, Ghanadi K, et al. Ethnic and geographic differentiation of *Helicobacter pylori* within Iran. PLoS One. 2010;5(3):e9645. doi:10.1371/journal.pone.0009645. 20339588 PMC2842290

[13] Vale FF, Vadivelu J, Oleastro M, Breurec S, Engstrand L, Perets TT, Mégraud F, Lehours P. Dormant phages of *Helicobacter pylori* reveal distinct populations in Europe. Sci Rep. 2015;5(1):14333. doi:10.1038/srep14333. 26387443 PMC4585682

[14] Berthenet E, Yahara K, Thorell K, Pascoe B, Meric G, Mikhail JM, Engstrand L, Enroth H, Burette A, Megraud F, et al. A GWAS on *Helicobacter pylori* strains points to genetic variants associated with gastric cancer risk. BMC Biol. 2018;16(1):84. doi:10.1186/s12915-018-0550-3. 30071832 PMC6090961

[15] Tuan VP, Yahara K, Dung HDQ, Binh TT, Huu Tung P, Tri TD, Thuan NPM, Van KV, Trang TTH, Phuc BH, et al. Genome-wide association study of gastric cancer- and duodenal ulcer-derived *Helicobacter pylori* strains reveals discriminatory genetic variations and novel oncoprotein candidates. Microb genomics. 2021;7(11). doi:10.1099/mgen.0.000680 PMC8743543 34846284

[16] Gunaletchumy SP, Seevasant I, Tan MH, Croft LJ, Mitchell HM, Goh KL, Loke MF, Vadivelu J. *Helicobacter pylori* genetic diversity and gastro-duodenal diseases in Malaysia. Sci Rep. 2015;4(1):7431. doi:10.1038/srep07431. PMC5377019 25503415

[17] Ailloud F, Didelot X, Woltemate S, Pfaffinger G, Overmann J, Bader RC, Schulz C, Malfertheiner P, Suerbaum S. Within-host evolution of *Helicobacter pylori* shaped by niche-specific adaptation, intragastric migrations and selective sweeps. Nat Commun. 2019;10(1):2273. doi:10.1038/s41467-019-10050-1. 31118420 PMC6531487

[18] Lieberman TD, Flett KB, Yelin I, Martin TR, McAdam AJ, Priebe GP, Kishony R. Genetic variation of a bacterial pathogen within individuals with cystic fibrosis provides a record of selective pressures. Nat Genet. 2014;46(1):82–87. doi:10.1038/ng.2848. 24316980 PMC3979468

[19] Alikhan N-F, Petty NK, Ben Zakour NL, Beatson SA. BLAST Ring Image Generator (BRIG): simple prokaryote genome comparisons. BMC Genomics. 2011;12(1):402. doi:10.1186/1471-2164-12-402. 21824423 PMC3163573

[20] Bolger AM, Lohse M, Usadel B. Trimmomatic: aflexible trimmer for Illumina sequence data. Bioinformatics. 2014;30(15):2114–2120. doi:10.1093/bioinformatics/btu170. 24695404 PMC4103590

[21] Joshi N, Fass J. 2011. Sickle: a sliding-window, adaptive, quality-based trimming tool for FastQ files. [accessed 2014 Jul 23]. https://github.com/najoshi/sickle.

[22] Langmead B, Salzberg SL. Fast gapped-read alignment with Bowtie 2. Nat Methods. 2012;9(4):357–359. doi:10.1038/nmeth.1923. 22388286 PMC3322381

[23] Garrison E, Marth G. 2012. Haplotype-based variant detection from short-read sequencing. [accessed 2019 Jun 3]. http://arxiv.org/abs/1207.3907

[24] Garrison E. Vcflib, a simple C++ library for parsing and manipulating VCF files. bioRxiv. 2016. doi:10.1101/2021.05.21.445151.

[25] Li H, Handsaker B, Wysoker A, Fennell T, Ruan J, Homer N, Marth G, Abecasis G, Durbin R. 1000 genome project data processing subgroup 1000 genome project data processing. The sequence alignment/map format and SAMtools. Bioinformatics. 2009;25(16):2078–2079. doi:10.1093/bioinformatics/btp352. 19505943 PMC2723002

[26] Kennemann L, Didelot X, Aebischer T, Kuhn S, Drescher B, Droege M, Reinhardt R, Correa P, Meyer TF, Josenhans C, et al. *Helicobacter pylori* genome evolution during human infection. Proc Natl Acad Sci. 2011;108(12):5033–5038. doi:10.1073/pnas.1018444108. 21383187 PMC3064335

[27] Didelot X, Nell S, Yang I, Woltemate S, van der Merwe S, Suerbaum S. Genomic evolution and transmission of *Helicobacter pylori* in two South African families. Proc Natl Acad Sci. 2013;110(34):13880–13885. doi:10.1073/pnas.1304681110. 23898187 PMC3752273

[28] Raymond J, Thiberg J-M, Chevalier C, Kalach N, Bergeret M, Labigne A, Dauga C. Genetic and transmission analysis of *Helicobacter pylori* strains within a family. Emerg Infect Dis. 2004;10(10):1816–1821. doi:10.3201/eid1010.040042. 15504269 PMC3323258

[29] Kivi M, Tindberg Y, Sörberg M, Casswall TH, Befrits R, Hellström PM, Bengtsson C, Engstrand L, Granström M. Concordance of *Helicobacter pylori* strains within families. J Clin Microbiol. 2003;41(12):5604–5608. doi:10.1128/JCM.41.12.5604-5608.2003. 14662948 PMC309035

[30] López-Vidal Y, Ponce-de-León S, Castillo-Rojas G, Barreto-Zúñiga R, Torre-Delgadillo A. High diversity of *vacA* and *cagA Helicobacter pylori* genotypes in patients with and without gastric cancer. PLoS One. 2008;3(12):e3849. doi:10.1371/journal.pone.0003849. 19050763 PMC2585809

[31] Matteo MJ, Granados G, Perez CV, Olmos M, Sanchez C, Catalano M. *Helicobacter pylori cag* pathogenicity island genotype diversity within the gastric niche of a single host. J Med Microbiol. 2007;56(5):664–669. doi:10.1099/jmm.0.46885-0. 17446291

[32] Israel DA, Salama N, Krishna U, Rieger UM, Atherton JC, Falkow S, Peek RM Jr. *Helicobacter pylori* genetic diversity within the gastric niche of a single human host. Proc Natl Acad Sci U S A. 2001;98(25):14625–14630. doi:10.1073/pnas.251551698. 11724955 PMC64732

[33] Reyes-Leon A, Atherton JC, Argent RH, Puente JL, Torres J. Heterogeneity in the activity of Mexican *Helicobacter pylori* strains in gastric epithelial cells and its association with diversity in the *cagA* gene. Infect Immun. 2007;75(7):3445–3454. doi:10.1128/IAI.01951-06. 17438024 PMC1932923

[34] Noto JM, Chopra A, Loh JT, Romero-Gallo J, Piazuelo MB, Watson M, Leary S, Beckett AC, Wilson KT, Cover TL, et al. Pan-genomic analyses identify key *Helicobacter pylori* pathogenic loci modified by carcinogenic host microenvironments. Gut. 2017;1–12. doi:10.1136/gutjnl-2017-313863. PMC5857411 28924022

[35] Kuipers EJ, Israel DA, Kusters JG, Gerrits MM, Weel J, van Der Ende A, van Der Hulst RW, Wirth HP, Höök-Nikanne J, Thompson SA, et al. Quasispecies development of *Helicobacter pylori* observed in paired isolates obtained years apart from the same host. J Infect Dis. 2000;181(1):273–282. doi:10.1086/315173. 10608776 PMC2766531

[36] Thorell K, Hosseini S, Palacios Gonzáles RVP, Chaotham C, Graham DY, Paszat L, Rabeneck L, Lundin SB, Nookaew I, Sjöling Å. Identification of a Latin American-specific BabA adhesin variant through whole genome sequencing of *Helicobacter pylori* patient isolates from Nicaragua. BMC Evol Biol. 2016;16(1):1–16. doi:10.1186/s12862-016-0619-y. 26928576 PMC4770546

[37] Alm RA, Bina J, Andrews BM, Doig P, Hancock RE, Trust TJ. Comparative genomics of *Helicobacter pylori*: analysis of the outer membrane protein families. Infect Immun. 2000;68(7):4155–4168. doi:10.1128/IAI.68.7.4155-4168.2000. 10858232 PMC101716

[38] Kim A, Servetas SL, Kang J, Kim J, Jang S, Choi YH, Su H, Jeon Y-E, Hong YA, Yoo Y-J, et al. *Helicobacter pylori* outer membrane protein, HomC, shows geographic dependent polymorphism that is influenced by the Bab family. J Microbiol. 2016;54(12):846–852. doi:10.1007/s12275-016-6434-8. 27888458

[39] Bauwens E, Joosten M, Taganna J, Rossi M, Debraekeleer A, Tay A, Peters F, Backert S, Fox J, Ducatelle R, et al. In silico proteomic and phylogenetic analysis of the outer membrane protein repertoire of gastric *Helicobacter* species. Sci Rep. 2018;8(1):15453. doi:10.1038/s41598-018-32476-1. 30337679 PMC6194013

[40] Solnick JV, Hansen LM, Salama NR, Boonjakuakul JK, Syvanen M. Modification of *Helicobacter pylori* outer membrane protein expression during experimental infection of rhesus macaques. Proc Natl Acad Sci U S A. 2004;101(7):2106–2111. doi:10.1073/pnas.0308573100. 14762173 PMC357059

[41] Liu H, Fero JB, Mendez M, Carpenter BM, Servetas SL, Rahman A, Goldman MD, Boren T, Salama NR, Merrell DS, et al. Analysis of a single *Helicobacter pylori* strain over a 10-year period in a primate model. Int J Med Microbiol. 2015;305(3):392–403. doi:10.1016/j.ijmm.2015.03.002. 25804332 PMC4376324

[42] Yamaoka Y, Ojo O, Fujimoto S, Odenbreit S, Haas R, Gutierrez O, El-Zimaity HMT, Reddy R, Arnqvist A, Graham DY. *Helicobacter pylori* outer membrane proteins and gastroduodenal disease. Gut. 2006;55(6):775–781. doi:10.1136/gut.2005.083014. 16322107 PMC1856239

[43] Peters TM, Owen RJ, Slater E, Varea R, Teare EL, Saverymuttu S. Genetic diversity in the *Helicobacter pylori cag* pathogenicity island and effect on expression of anti-CagA serum antibody in UK patients with dyspepsia. J Clin Pathol. 2001;54(3):219–223. doi:10.1136/JCP.54.3.219. 11253135 PMC1731375

[44] Heep M, Rieger U, Beck D, Lehn N. Mutations in the beginning of the *rpoB* gene can induce resistance to rifamycins in both *Helicobacter pylori* and *Mycobacterium tuberculosis*. Antimicrob Agents Chemother. 2000;44(4):1075–1077. doi:10.1128/AAC.44.4.1075-1077.2000. 10722516 PMC89817

[45] Heep M, Odenbreit S, Beck D, Decker J, Prohaska E, Rieger U, Lehn N. Mutations at four distinct regions of the *rpoB* gene can reduce the susceptibility of *Helicobacter pylori* to rifamycins. Antimicrob Agents Chemother. 2000;44(6):1713–1715. doi:10.1128/AAC.44.6.1713-1715.2000. 10817737 PMC89941

[46] Binh TT, Shiota S, Suzuki R, Matsuda M, Trang TTH, Kwon DH, Iwatani S, Yamaoka Y. Discovery of novel mutations for clarithromycin resistance in *Helicobacter pylori* by using next-generation sequencing. J Antimicrob Chemother. 2014;69(7):1796–1803. doi:10.1093/jac/dku050. 24648504 PMC4054984

[47] Geng X, Li W, Chen Z, Gao S, Hong W, Ge X, Hou G, Hu Z, Zhou Y, Zeng B, et al. The bifunctional enzyme SpoT Is Involved in the clarithromycin tolerance of *Helicobacter pylori* by upregulating the transporters HP0939, HP1017, HP0497, and HP0471. Antimicrob Agents Chemother. 2017;61(5). doi:10.1128/AAC.02011-16 PMC5404559 28242673

[48] Cerda OA, Núñez-Villena F, Soto SE, Ugalde JM, López-Solís R, Toledo H. tlpA gene expression is required for arginine and bicarbonate chemotaxis in *Helicobacter pylori*. Biol Res. 2011;44(3):277–282. doi:10.4067/S0716-97602011000300009. 22688915

[49] Huang JY, Goers Sweeney E, Guillemin K, Amieva MR. Multiple acid sensors control *Helicobacter pylori* colonization of the stomach. PLoS Pathog. 2017;13(1):e1006118. doi:10.1371/journal.ppat.1006118. 28103315 PMC5245789

[50] Croxen MA, Sisson G, Melano R, Hoffman PS. The *Helicobacter pylori* chemotaxis receptor TlpB (HP0103) is required for pH taxis and for colonization of the gastric mucosa. J Bacteriol. 2006;188(7):2656–2665. doi:10.1128/JB.188.7.2656-2665.2006. 16547053 PMC1428400

[51] Furuta Y, Konno M, Osaki T, Yonezawa H, Ishige T, Imai M, Shiwa Y, Shibata-Hatta M, Kanesaki Y, Yoshikawa H, et al. Microevolution of virulence-related genes in *Helicobacter pylori* familial infection. PLoS One. 2015;10(5):1–17. doi:10.1371/journal.pone.0127197. PMC4433339 25978460

[52] Nobusato A, Uchiyama I, Kobayashi I. Diversity of restriction-modification gene homologues in *Helicobacter pylori*. Gene. 2000;259(1–2):89–98. doi:10.1016/s0378-1119(00)00455-8. 11163966

[53] Aras RA, Small AJ, Ando T, Blaser MJ. *Helicobacter pylori* interstrain restriction-modification diversity prevents genome subversion by chromosomal DNA from competing strains. Nucleic Acids Res. 2002;30(24):5391–5397. doi:10.1093/nar/gkf686. 12490707 PMC140068

[54] Vasu K, Nagamalleswari E, Nagaraja V. Promiscuous restriction is a cellular defense strategy that confers fitness advantage to bacteria. Proc Natl Acad Sci U S A. 2012;109(20):E1287–93. doi:10.1073/pnas.1119226109. 22509013 PMC3356625

[55] Bubendorfer S, Krebes J, Yang I, Hage E, Schulz TF, Bahlawane C, Didelot X, Suerbaum S. Genome-wide analysis of chromosomal import patterns after natural transformation of *Helicobacter pylori*. Nat Commun. 2016;7(1):11995. doi:10.1038/ncomms11995. 27329939 PMC4917963

[56] Vitoriano I, Vítor JMB, Oleastro M, Roxo-Rosa M, Vale FF. Proteome variability among *Helicobacter pylori* isolates clustered according to genomic methylation. J Appl Microbiol. 2013;114(6):1817–1832. doi:10.1111/jam.12187. 23480599

[57] Srikhanta YN, Gorrell RJ, Steen JA, Gawthorne JA, Kwok T, Grimmond SM, Robins-Browne RM, Jennings MP. Phasevarion mediated epigenetic gene regulation in H*elicobacter pylori*. PLoS One. 2011;6(12):e27569. doi:10.1371/journal.pone.0027569. 22162751 PMC3230613

[58] Lehours P, Dupouy S, Chaineux J, Ruskoné-Fourmestraux A, Delchier J-C, Morgner A, Mégraud F, Ménard A. Genetic diversity of the HpyC1I restriction modification system in *Helicobacter pylori*. Res Microbiol. 2007;158(3):265–271. doi:10.1016/J.RESMIC.2006.12.002. 17346936

[59] Gauntlett JC, Nilsson H-O, Fulurija A, Marshall BJ, Benghezal M. Phase-variable restriction/modification systems are required for *Helicobacter pylori* colonization. Gut Pathogens. 2014;6:35. doi:10.1186/s13099-014-0035-z. 25349630 PMC4209511

[60] Liechti G, Goldberg JB. Outer membrane biogenesis in *Escherichia coli*, Neisseria meningitidis, and Helicobacter pylori: paradigm deviations in *H. pylori*. Front Cell Infect Microbiol. 2012;2:29. doi:10.3389/fcimb.2012.00029. 22919621 PMC3417575

[61] Rubin EJ, Trent MS. Colonize, evade, flourish: how glyco-conjugates promote virulence of *Helicobacter pylori*. Gut Microbes. 2013;4(6):439–453. doi:10.4161/gmic.25721. 23859890 PMC3928157

[62] Fung C, Tan S, Nakajima M, Skoog EC, Camarillo-Guerrero LF, Klein JA, Lawley TD, Solnick JV, Fukami T, Amieva MR. High-resolution mapping reveals that microniches in the gastric glands control *Helicobacter pylori* colonization of the stomach. PLOS Biol. 2019;17(5):e3000231. doi:10.1371/journal.pbio.3000231. 31048876 PMC6497225

[63] Kaneko K, Zaitoun AM, Letley DP, Rhead JL, Torres J, Spendlove I, Atherton JC, Robinson K. The active form of *Helicobacter pylori* vacuolating cytotoxin induces decay-accelerating factor CD55 in association with intestinal metaplasia in the human gastric mucosa. J Pathol. 2022;258(2):199–209. doi:10.1002/path.5990. 35851954 PMC9543990

[64] Rhead JL, Letley DP, Mohammadi M, Hussein N, Mohagheghi MA, Eshagh Hosseini M, Atherton JC. A new *Helicobacter pylori* vacuolating cytotoxin determinant, the intermediate region, is associated with gastric cancer. Gastroenterology. 2007;133(3):926–936. doi:10.1053/j.gastro.2007.06.056. 17854597

[65] Atherton JC, Cao P, Peek RM, Tummuru MKR, Blaser MJ, Cover TL. Mosaicism in vacuolating cytotoxin alleles of *Helicobacter pylori*. J Biol Chem. 1995;270(30):17771–17777. doi:10.1074/jbc.270.30.17771. 7629077

[66] Andrews S. 2010. FastQC - A Quality Control application for FastQ files. [accessed 2018 Jan 10]. https://github.com/s-andrews/FastQC

[67] Wood DE, Salzberg SL. Kraken: ultrafast metagenomic sequence classification using exact alignments. Genome Biol. 2014;15(3):R46. doi:10.1186/gb-2014-15-3-r46. 24580807 PMC4053813

[68] Bankevich A, Nurk S, Antipov D, Gurevich AA, Dvorkin M, Kulikov AS, Lesin VM, Nikolenko SI, Pham S, Prjibelski AD, et al. SPAdes: a new genome assembly algorithm and its applications to single-cell sequencing. J Comput Biol. 2012;19(5):455–477. doi:10.1089/cmb.2012.0021. 22506599 PMC3342519

[69] Pedersen BS, Quinlan AR. Mosdepth: quick coverage calculation for genomes and exomes. Bioinformatics. 2018;34(5):867–868. doi:10.1093/bioinformatics/btx699. 29096012 PMC6030888

[70] Seemann T. Prokka: rapid prokaryotic genome annotation. Bioinformatics. 2014;30(14):2068–2069. doi:10.1093/bioinformatics/btu153. 24642063

[71] Seemann T. 2015. snippy: fast bacterial variant calling from NGS reads. [accessed 2019 July 15]. https://github.com/tseemann/snippy

[72] Darling ACE, Mau B, Blattner FR, Perna NT. Mauve: multiple alignment of conserved genomic sequence with rearrangements. Genome Res. 2004;14(7):1394–1403. doi:10.1101/gr.2289704. 15231754 PMC442156

[73] Cingolani P, Platts A, Wang LL, Coon M, Nguyen T, Wang L, Land SJ, Lu X, Ruden DM. A program for annotating and predicting the effects of single nucleotide polymorphisms, SnpEff: sNPs in the genome of *Drosophila melanogaster* strain w1118; iso-2; iso-3. Fly. 2012;6(2):80–92. doi:10.4161/fly.19695. 22728672 PMC3679285

[74] Keane JA, Page AJ, Delaney AJ, Taylor B, Seemann T, Harris SR, Soares J. SNP-sites: rapid efficient extraction of SNPs from multi-FASTA alignments. Microbiol Genomics. 2016;2(4). doi:10.1099/mgen.0.000056. PMC5320690 28348851

[75] Price MN, Dehal PS, Arkin AP. FastTree 2 – approximately maximum-likelihood trees for large alignments. PLoS One. 2010;5(3):e9490. doi:10.1371/journal.pone.0009490. 20224823 PMC2835736

[76] Tonkin-Hill G, MacAlasdair N, Ruis C, Weimann A, Horesh G, Lees JA, Gladstone RA, Lo S, Beaudoin C, Floto RA, et al. Producing polished prokaryotic pangenomes with the Panaroo pipeline. Genome Biol. 2020;21(1):180. doi:10.1186/s13059-020-02090-4. 32698896 PMC7376924

[77] R Core Team. R: a language and environment for statistical computing. 2018 R Foundation for Statistical Computing, Vienna (Austria). [accessed 2018 July 31]. https://www.r-project.org/

[78] Hanbo C. 2018. VennDiagram: generate High-Resolution Venn and Euler Plots. [accessed 2018 July 31]. https://rdrr.io/cran/VennDiagram

