# Supplementary material for: Genomic diversity of Helicobacter pylori populations from different regions of the human stomach
Source: Gut Microbes. 2022 Dec 5;14(1):2152306. doi: 10.1080/19490976.2022.2152306 (PMC9728471; doi:10.1080/19490976.2022.2152306)
Supplement: Supplemental Material [file KGMI_A_2152306_SM1608.zip › Suppl Figure and table legends.docx]

**Suppl. Table 1**. List of gene ID’s used within this study with corresponding gene product annotations. Gene abbreviations are also provided. Where a gene ID is not listed in this table, the gene was identified and annotated through the PROKKA^21^ database.

**Suppl. Table 2**. List of genes with minor allele variation. Hypothetical genes are included along with the corresponding patient ID. The total number of allelic sites within genes across samples as well as the number of deep sequenced population (n=33) with identifiable minor allelic variants are displayed. Gene abbreviations and products associated with gene IDs can be found in Suppl. Table 1 for cross-reference.

**Suppl. Table 3**. Table denoting the total number of common allelic variation, minor allelic variation and multiple nucleotide polymorphisms (MNPs – potentially highlighting recombination events) found within each sample (n=33). The length of infection of the deep sequenced populations at a snapshot in time is also calculated using the methods described in the materials and methods section.

**Suppl. Fig. 1**. Whole genome alignment for *H. pylori* strains isolated from patient 45. Whole genome alignments of the consensus genomes generated by deep population sequencing of antrum and corpus *H. pylori* populations, using antrum (A) or corpus (B) consensus genome as the reference. Colour intensity of the outermost ring indicates percentage identity between antrum and corpus consensus genomes. Positions of contigs within the assembled reference genome are shown as a ring alternating in colour between yellow and black. Blue bars in the coverage ring show regions >700X. Plots generated using BRIG.

**Suppl. Fig. 2**. Whole genome alignment for *H. pylori* strains isolated from patient 77. Whole genome alignments of the consensus genomes generated by deep population sequencing of antrum and corpus *H. pylori* populations, using antrum (A) or corpus (B) consensus genome as the reference. Colour intensity of the outermost ring indicates percentage identity between antrum and corpus consensus genomes. Positions of contigs within the assembled reference genome are shown as a ring alternating in colour between yellow and black. Blue bars in the coverage ring show regions >700X. Plots generated using BRIG.

**Suppl. Fig. 3**. Whole genome alignment for *H. pylori* strains isolated from patient 93. Whole genome alignments of the consensus genomes generated by deep population sequencing of antrum and corpus *H. pylori* populations, using antrum (A) or corpus (B) consensus genome as the reference. Colour intensity of the outermost ring indicates percentage identity between antrum and corpus consensus genomes. Positions of contigs within the assembled reference genome are shown as a ring alternating in colour between yellow and black. Blue bars in the coverage ring show regions >700X. Plots generated using BRIG.

**Suppl. Fig. 4**. Whole genome alignment for *H. pylori* strains isolated from patient 120. Whole genome alignments of the consensus genomes generated by deep population sequencing of antrum and corpus *H. pylori* populations, using antrum (A) or corpus (B) consensus genome as the reference. Colour intensity of the outermost ring indicates percentage identity between antrum and corpus consensus genomes. Positions of contigs within the assembled reference genome are shown as a ring alternating in colour between yellow and black. Blue bars in the coverage ring show regions >700X. Plots generated using BRIG.

**Suppl. Fig. 5.** Representative whole genome alignment for *H. pylori* strains isolated from patient 194. Whole genome alignments of the consensus genomes generated by deep population sequencing of antrum and corpus *H. pylori* populations, using antrum (A) or corpus (B) consensus genome as the reference. Panel C depicts the alignment of colony isolate assembled genomes against the patient reference (assembly created by combining the curated reads from both antrum and corpus regions). Colour intensity of the outermost ring indicates percentage identity between antrum and corpus consensus genomes. Positions of contigs within the assembled reference genome are shown as a ring alternating in colour between yellow and black. Blue bars in the coverage ring show regions >700X. Plots generated using BRIG.

**Suppl. Fig. 6**. Whole genome alignment for *H. pylori* strains isolated from patient 265. Whole genome alignments of the consensus genomes generated by deep population sequencing of antrum and corpus *H. pylori* populations, using antrum (A) or corpus (B) consensus genome as the reference. Colour intensity of the outermost ring indicates percentage identity between antrum and corpus consensus genomes. Positions of contigs within the assembled reference genome are shown as a ring alternating in colour between yellow and black. Blue bars in the coverage ring show regions >700X. Plots generated using BRIG.

**Suppl. Fig. 7.** Representative whole genome alignment for *H. pylori* strains isolated from patient 295. Whole genome alignments of the consensus genomes generated by deep population sequencing of antrum and corpus *H. pylori* populations, using antrum (A) or corpus (B) consensus genome as the reference. Panel C depicts the alignment of colony isolate assembled genomes against the patient reference (assembly created by combining the curated reads from both antrum and corpus regions). Colour intensity of the outermost ring indicates percentage identity between antrum and corpus consensus genomes. Positions of contigs within the assembled reference genome are shown as a ring alternating in colour between yellow and black. Blue bars in the coverage ring show regions >700X. Plots generated using BRIG.

**Suppl. Fig. 8**. Whole genome alignment for *H. pylori* strains isolated from patient 308. Whole genome alignments of the consensus genomes generated by deep population sequencing of antrum and corpus *H. pylori* populations, using antrum (A) or corpus (B) consensus genome as the reference. Colour intensity of the outermost ring indicates percentage identity between antrum and corpus consensus genomes. Positions of contigs within the assembled reference genome are shown as a ring alternating in colour between yellow and black. Blue bars in the coverage ring show regions >700X. The antrum contained contigs that were not of *H. pylori* origin and were removed from the dataset for all other analysis. Plots generated using BRIG.

**Suppl. Fig. 9.** Representative whole genome alignment for *H. pylori* strains isolated from patient 326. Whole genome alignments of the consensus genomes generated by deep population sequencing of antrum and corpus *H. pylori* populations, using antrum (A) or corpus (B) consensus genome as the reference. Panel C depicts the alignment of colony isolate assembled genomes against the deep sequenced *H. pylori* population consensus assembled genome from the corpus dataset due to contaminant reads present in the deep sequenced population in the antral region. Colour intensity of the outermost ring indicates percentage identity between antrum and corpus consensus genomes. Positions of contigs within the assembled reference genome are shown as a ring alternating in colour between yellow and black. Blue bars in the coverage ring show regions >700X. The deep sequenced *H. pylori* population from the antrum contained contigs that were not of *H. pylori* origin and were removed from the dataset for all other analysis. Plots generated using BRIG.

**Suppl. Fig. 10**. Representative whole genome alignment for *H. pylori* strains isolated from patient 439. Whole genome alignments of the consensus genomes generated by deep population sequencing of antrum and corpus *H. pylori* populations, using antrum (A) or corpus (B) consensus genome as the reference. Panel C depicts the alignment of colony isolate assembled genomes against the patient reference (assembly created by combining the curated reads from both antrum and corpus regions). Colour intensity of the outermost ring indicates percentage identity between antrum and corpus consensus genomes. Positions of contigs within the assembled reference genome are shown as a ring alternating in colour between yellow and black. Blue bars in the coverage ring show regions >700X. Where there were gaps in the query genomes, the genes were annotated and overlaid to highlight the missing genes. A list of the gene product descriptions for each gene ID (HP number) can be found in Suppl. Table 1. Plots generated using BRIG.

**Suppl. Fig. 11.** Representative whole genome alignment for *H. pylori* strains isolated from patient 444. Whole genome alignments of the consensus genomes generated by deep population sequencing of antrum and corpus *H. pylori* populations, using antrum (A) or corpus (B) consensus genome as the reference. Panel C depicts the alignment of colony isolate assembled genomes against the patient reference (assembly created by combining the curated reads from both antrum and corpus regions). Colour intensity of the outermost ring indicates percentage identity between antrum and corpus consensus genomes. Positions of contigs within the assembled reference genome are shown as a ring alternating in colour between yellow and black. Blue bars in the coverage ring show regions >700X. Plots generated using BRIG.

**Suppl. Fig. 12.** Representative whole genome alignment for *H. pylori* strains isolated from patient 495. Whole genome alignments of the consensus genomes generated by deep population sequencing of antrum and corpus *H. pylori* populations, using antrum (A) or corpus (B) consensus genome as the reference. Panel C depicts the alignment of colony isolate assembled genomes against the patient reference (assembly created by combining the curated reads from both antrum and corpus regions). Colour intensity of the outermost ring indicates percentage identity between antrum and corpus consensus genomes. Positions of contigs within the assembled reference genome are shown as a ring alternating in colour between yellow and black. Blue bars in the coverage ring show regions >700X. Where there were gaps in the query genomes, the genes were annotated and overlaid to highlight the missing genes. A list of the gene product descriptions for each gene ID (HP number) can be found in Suppl. Table 1. Plots generated using BRIG.

**Suppl. Fig. 13.** Representative whole genome alignment for *H. pylori* strains isolated from patient 565. Whole genome alignments of the consensus genomes generated by deep population sequencing of antrum and corpus *H. pylori* populations, using antrum (A) or corpus (B) consensus genome as the reference. Panel C depicts the alignment of colony isolate assembled genomes against the patient reference (assembly created by combining the curated reads from both antrum and corpus regions). Colour intensity of the outermost ring indicates percentage identity between antrum and corpus consensus genomes. Positions of contigs within the assembled reference genome are shown as a ring alternating in colour between yellow and black. Blue bars in the coverage ring show regions >700X. Where there were gaps in the query genomes, the genes were annotated and overlaid to highlight the missing genes. A list of the gene product descriptions for each gene ID (HP number) can be found in Suppl. Table 1. Plots generated using BRIG.

**Suppl. Fig. 14.** Representative whole genome alignment for *H. pylori* strains isolated from patient 732. Whole genome alignments of the consensus genomes generated by deep population sequencing of antrum and corpus *H. pylori* populations, using antrum (A) or corpus (B) consensus genome as the reference (assembly created by combining the curated reads from both antrum and corpus regions). Panel C depicts the alignment of colony isolate assembled genomes against the patient reference. Colour intensity of the outermost ring indicates percentage identity between antrum and corpus consensus genomes. Positions of contigs within the assembled reference genome are shown as a ring alternating in colour between yellow and black. Blue bars in the coverage ring show regions >700X. Plots generated using BRIG.

**Suppl. Fig. 15**. Alignment of colony isolate assembled genomes from the corpus region of patient 249 against the deep sequenced consensus assembled population reference (249C). Colour intensity of the outermost ring indicates percentage identity between antrum and corpus consensus genomes. Plots generated using BRIG. Where there were gaps in the query genomes, the genes were annotated and overlaid to highlight the missing genes. A list of the gene product descriptions for each gene ID (HP number) can be found in Suppl. Table 1.

**Suppl. Fig. 16**. Alignment of colony isolate assembled genomes from the antral region of patient 537 against the deep sequenced consensus assembled population reference (537A). Colour intensity of the outermost ring indicates percentage identity between antrum and corpus consensus genomes. Plots generated using BRIG.

**Suppl. Fig. 17**. The curated SNPs (Supp. Fig. 23) identified by the combined whole genome alignment and alternative stomach region mapping methodologies (depicted in Suppl. Fig. 21 and defined in the materials and methods) were used to identify the number of synonymous or nonsynonymous mutations. A paired t-test was with the total numbers of synonymous and nonsynonymous mutations between the paired populations was carried out.

**Suppl. Fig. 18**. Pan-genome analysis of all single colony isolates used within this study. Single colony isolates grouped together when isolated from the same patient. Patient 1 (patient samples 295 and 326 which were identified as sequentially isolated patient biopsies), patient 2 (patient samples 249 and 537 which were identified as sequentially isolated patient biopsies), patient 3 (patient samples 194), patient 4 (patient samples 565), patient 5 (patient samples 439), patient 6 (patient samples 322), patient 7 (patient samples 732), patient 8 (patient samples 495), patient 9 (patient samples 444).

**Suppl. Fig 19.** Heat map showing all common allelic variation across genes/gene products for each antrum- and corpus-derived *H. pylori* population, derived from deep sequencing of the population from each biopsy and read-mapping back to the consensus genome to identify variant bases at each locus (Table 1). Higher colour intensity indicates a larger number of variant bases within that gene/gene product. A list of the gene product descriptions for each gene ID (HP number) can be found in Suppl. Table 1.

**Suppl. Fig 20.** Heat map showing all minor allelic variation across genes/gene products for each antrum- and corpus-derived *H. pylori* population, derived from deep sequencing of the population from each biopsy and read-mapping back to the consensus genome to identify variant bases at each locus (Table 1). Higher colour intensity indicates a larger number of variant bases within that gene/gene product. A list of the gene product descriptions for each gene ID (HP number) can be found in Suppl. Table 1. A list of the minor allelic variants can be found in Suppl. Table 2.

**Supp. Fig. 21**. Heat map showing the most variable genes and populations identified by a combination of analytical approaches (within and between bacterial populations from the antrum and corpus regions of patient stomachs). Each column contains data from one patient. Within each column, three datasets are shown. From left to right these are: between stomach region variation from whole genome alignment antrum versus corpus (Supp. Fig. 23); within antrum minor allele variation (Table 1); within corpus minor allele variation (Table 1). Darker colour intensity indicates a larger number of variant bases within that gene/gene product. Only patients with paired antrum and corpus data were included. This figure combines the information presented in Fig. 2 plus Suppl. Fig. 20 (minor allelic variants) and the methodology depicted in Supp. Fig 23. A list of the gene product descriptions for each gene ID (HP number) can be found in Suppl. Table 1.

**Suppl. Fig. 22**. Venn diagram showing the percentage of allelic variant sites identified in this study by deep sequencing (blue) and single colony sequencing (pink) pipelines. Deep sequencing of the whole population detected the majority of the population diversity and was more powerful than the single colony method. However, some diversity (8.13%) was not identified by the deep sequencing dataset, presumably due to the stringent filtering and quality control methods used to minimise identification of false positives.

**Suppl. Fig. 23**. Between region diversity investigated using a consensus and read mapping alignment approach. This approach was taken to identify between region SNPs of high quality by supporting alignment SNPs with alternative stomach region read mapping to the opposite location consensus genome.
