# Supplementary material for: Genomic diversity of Helicobacter pylori populations from different regions of the human stomach
Source: Gut Microbes. 2022 Dec 5;14(1):2152306. doi: 10.1080/19490976.2022.2152306 (PMC9728471; doi:10.1080/19490976.2022.2152306)
Supplement: Supplemental Material [file KGMI_A_2152306_SM1608.zip › SupplFig1.pdf]

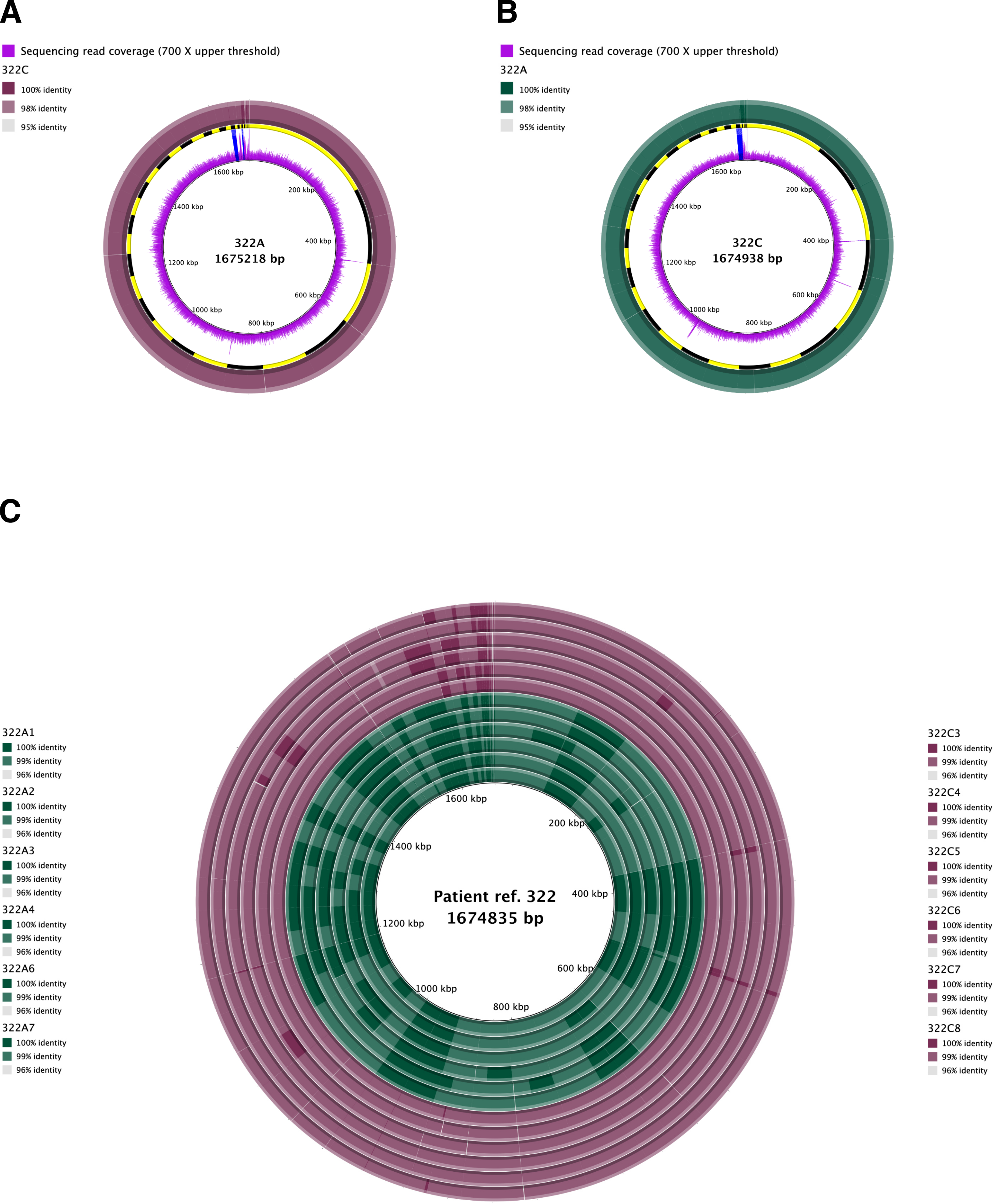
A
Sequencing read coverage (700 X upper threshold)
322C
100% identity
98% identity
95% identity
1600 kbp
200 kbp
1400 kbp
400 kbp
322A
1675218 bp
1200 kbp
600 kbp
1000 kbp
800 kbp
B
Sequencing read coverage (700 X upper threshold)
322A
100% identity
98% identity
95% identity
1600 kbp
200 kbp
1400 kbp
400 kbp
322C
1674938 bp
1200 kbp
600 kbp
1000 kbp
800 kbp
C
322A1
100% identity
99% identity
96% identity
322A2
100% identity
99% identity
96% identity
322A3
100% identity
99% identity
96% identity
322A4
100% identity
99% identity
96% identity
322A6
100% identity
99% identity
96% identity
322A7
100% identity
99% identity
96% identity
1600 kbp
200 kbp
1400 kbp
Patient ref. 322
1674835 bp
400 kbp
1200 kbp
600 kbp
1000 kbp
800 kbp
322C3
100% identity
99% identity
96% identity
322C4
100% identity
99% identity
96% identity
322C5
100% identity
99% identity
96% identity
322C6
100% identity
99% identity
96% identity
322C7
100% identity
99% identity
96% identity
322C8
100% identity
99% identity
96% identity
