# Supplementary material for: Genomic diversity of Helicobacter pylori populations from different regions of the human stomach
Source: Gut Microbes. 2022 Dec 5;14(1):2152306. doi: 10.1080/19490976.2022.2152306 (PMC9728471; doi:10.1080/19490976.2022.2152306)
Supplement: Supplemental Material [file KGMI_A_2152306_SM1608.zip › SupplFig10.pdf]

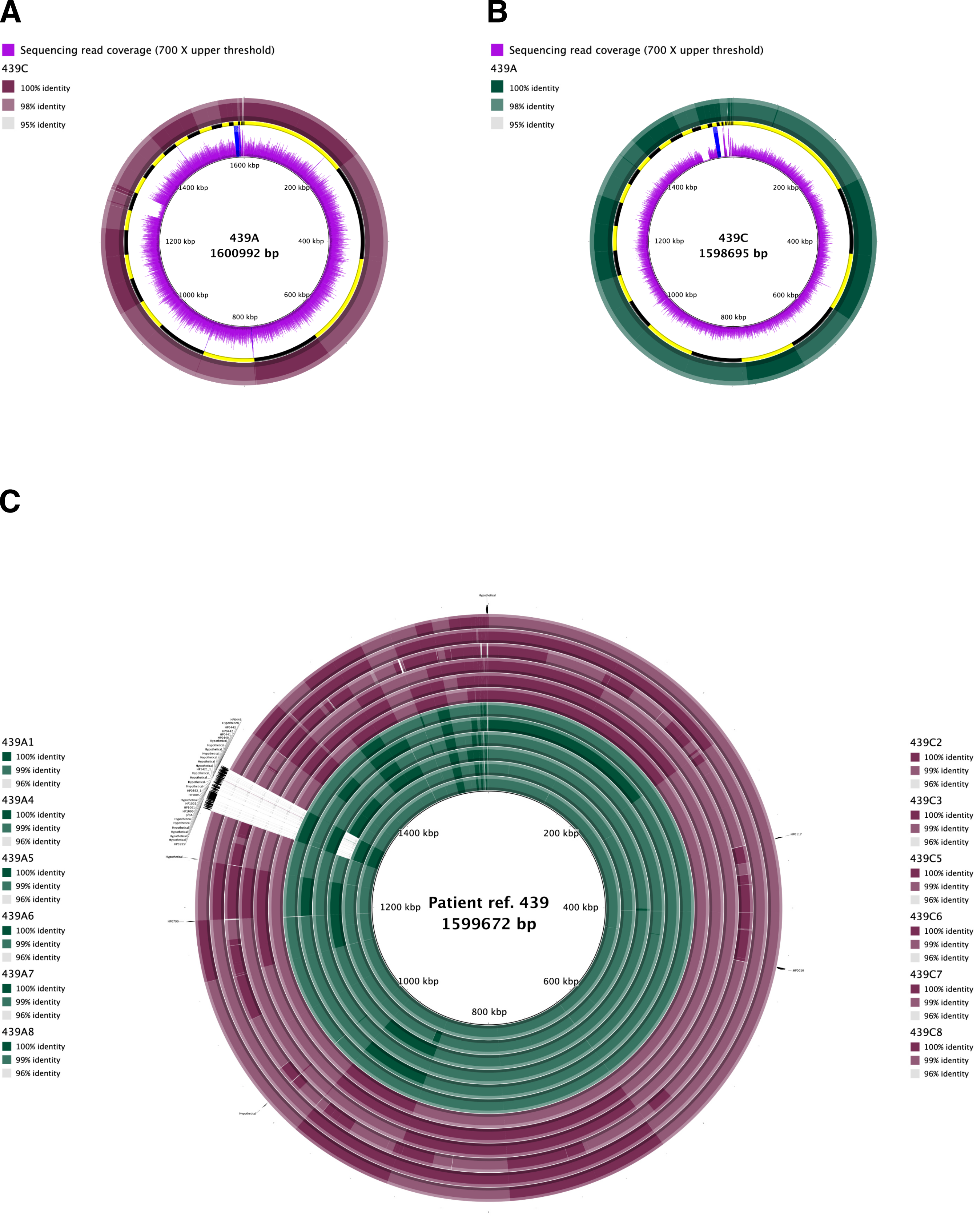
A
Sequencing read coverage (700 X upper threshold)
439C
100% identity
98% identity
95% identity
1600 kbp
200 kbp
400 kbp
600 kbp
800 kbp
1000 kbp
1200 kbp
1400 kbp
439A
1600992 bp
B
Sequencing read coverage (700 X upper threshold)
439A
100% identity
98% identity
95% identity
200 kbp
400 kbp
600 kbp
800 kbp
1000 kbp
1200 kbp
1400 kbp
439C
1598695 bp
C
439A1
100% identity
99% identity
96% identity
439A4
100% identity
99% identity
96% identity
439A5
100% identity
99% identity
96% identity
439A6
100% identity
99% identity
96% identity
439A7
100% identity
99% identity
96% identity
439A8
100% identity
99% identity
96% identity
439C2
100% identity
99% identity
96% identity
439C3
100% identity
99% identity
96% identity
439C5
100% identity
99% identity
96% identity
439C6
100% identity
99% identity
96% identity
439C7
100% identity
99% identity
96% identity
439C8
100% identity
99% identity
96% identity
Patient ref. 439
1599672 bp
200 kbp
400 kbp
600 kbp
800 kbp
1000 kbp
1200 kbp
1400 kbp
