# Supplementary material for: Genomic diversity of Helicobacter pylori populations from different regions of the human stomach
Source: Gut Microbes. 2022 Dec 5;14(1):2152306. doi: 10.1080/19490976.2022.2152306 (PMC9728471; doi:10.1080/19490976.2022.2152306)
Supplement: Supplemental Material [file KGMI_A_2152306_SM1608.zip › SupplFig11.pdf]

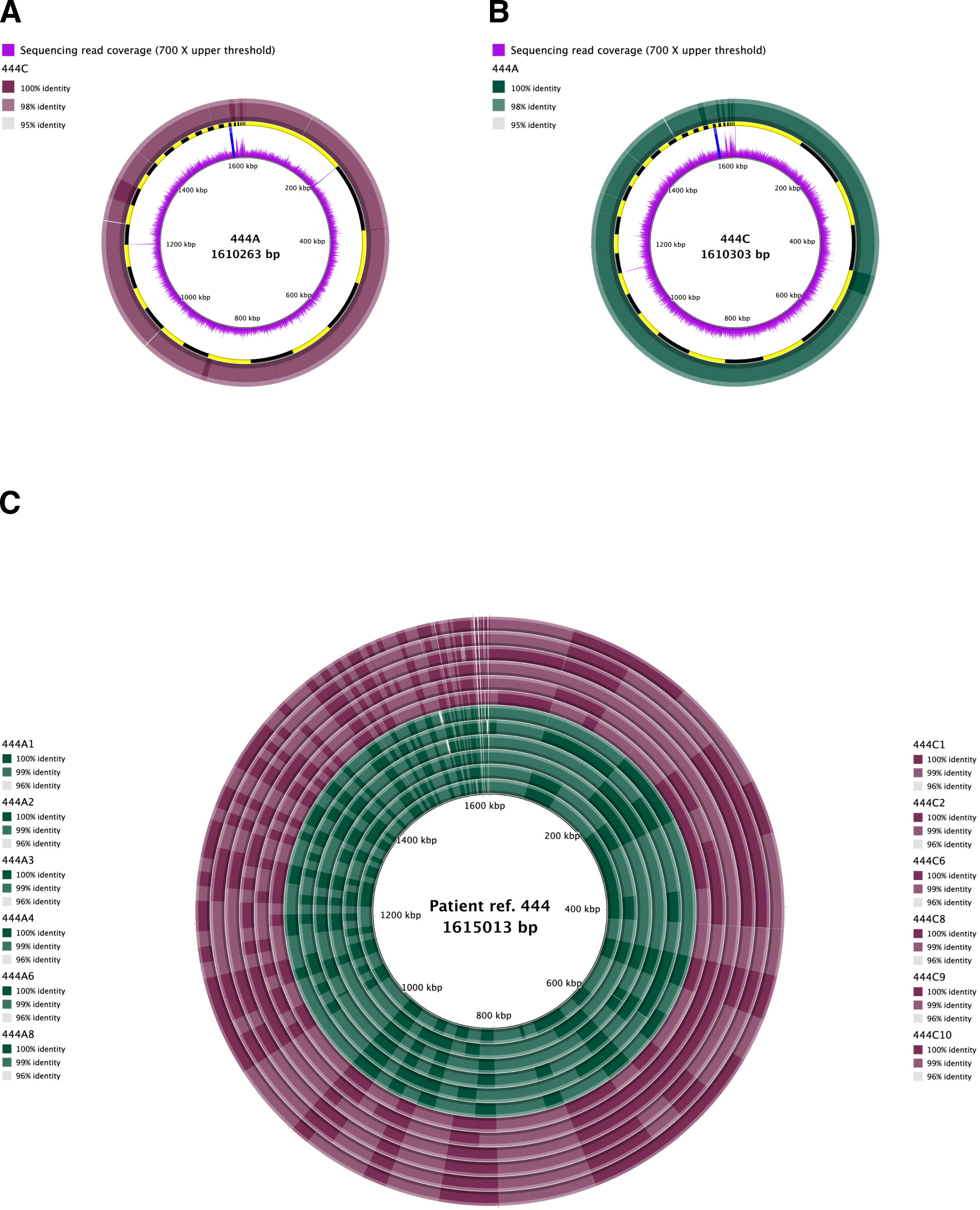
A
Sequencing read coverage (700 X upper threshold)
444C
100% identity
98% identity
95% identity
444A
1610263 bp
1600 kbp
200 kbp
400 kbp
600 kbp
800 kbp
1000 kbp
1200 kbp
1400 kbp
B
Sequencing read coverage (700 X upper threshold)
444A
100% identity
98% identity
95% identity
444C
1610303 bp
1600 kbp
200 kbp
400 kbp
600 kbp
800 kbp
1000 kbp
1200 kbp
1400 kbp
C
444A1
100% identity
99% identity
96% identity
444A2
100% identity
99% identity
96% identity
444A3
100% identity
99% identity
96% identity
444A4
100% identity
99% identity
96% identity
444A6
100% identity
99% identity
96% identity
444A8
100% identity
99% identity
96% identity
Patient ref. 444
1615013 bp
1600 kbp
200 kbp
400 kbp
600 kbp
800 kbp
1000 kbp
1200 kbp
1400 kbp
444C1
100% identity
99% identity
96% identity
444C2
100% identity
99% identity
96% identity
444C6
100% identity
99% identity
96% identity
444C8
100% identity
99% identity
96% identity
444C9
100% identity
99% identity
96% identity
444C10
100% identity
99% identity
96% identity
