# Supplementary material for: Genomic diversity of Helicobacter pylori populations from different regions of the human stomach
Source: Gut Microbes. 2022 Dec 5;14(1):2152306. doi: 10.1080/19490976.2022.2152306 (PMC9728471; doi:10.1080/19490976.2022.2152306)
Supplement: Supplemental Material [file KGMI_A_2152306_SM1608.zip › SupplFig13.pdf]

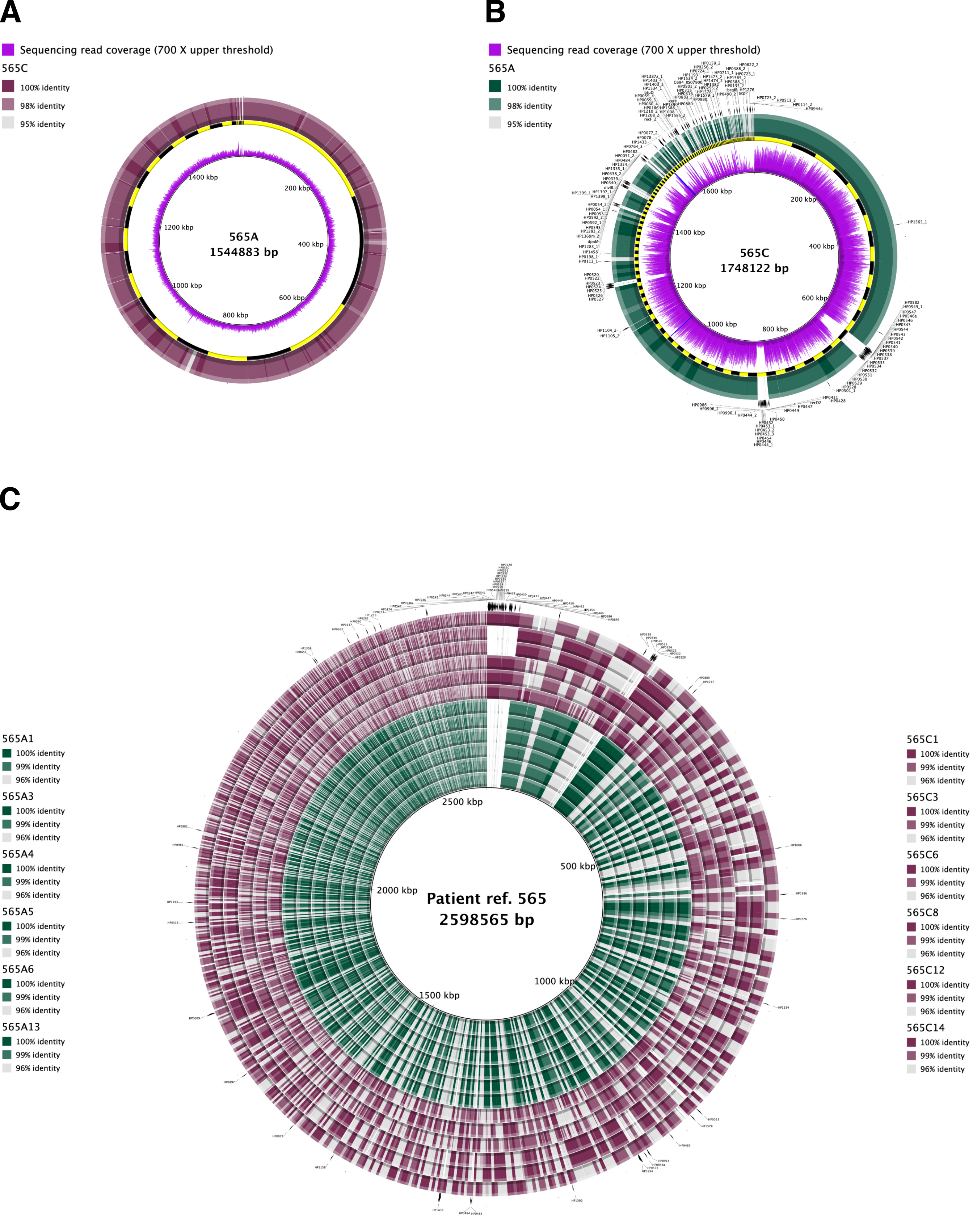
A
Sequencing read coverage (700 X upper threshold)
565C
100% identity
98% identity
95% identity
565A
1544883 bp
200 kbp
400 kbp
600 kbp
800 kbp
1000 kbp
1200 kbp
1400 kbp
B
Sequencing read coverage (700 X upper threshold)
565A
100% identity
98% identity
95% identity
565C
1748122 bp
200 kbp
400 kbp
600 kbp
800 kbp
1000 kbp
1200 kbp
1400 kbp
1600 kbp
C
565A1
100% identity
99% identity
96% identity
565A3
100% identity
99% identity
96% identity
565A4
100% identity
99% identity
96% identity
565A5
100% identity
99% identity
96% identity
565A6
100% identity
99% identity
96% identity
565A13
100% identity
99% identity
96% identity
Patient ref. 565
2598565 bp
500 kbp
1000 kbp
1500 kbp
2000 kbp
2500 kbp
565C1
100% identity
99% identity
96% identity
565C3
100% identity
99% identity
96% identity
565C6
100% identity
99% identity
96% identity
565C8
100% identity
99% identity
96% identity
565C12
100% identity
99% identity
96% identity
565C14
100% identity
99% identity
96% identity
