# Supplementary material for: Genomic diversity of Helicobacter pylori populations from different regions of the human stomach
Source: Gut Microbes. 2022 Dec 5;14(1):2152306. doi: 10.1080/19490976.2022.2152306 (PMC9728471; doi:10.1080/19490976.2022.2152306)
Supplement: Supplemental Material [file KGMI_A_2152306_SM1608.zip › SupplFig14.pdf]

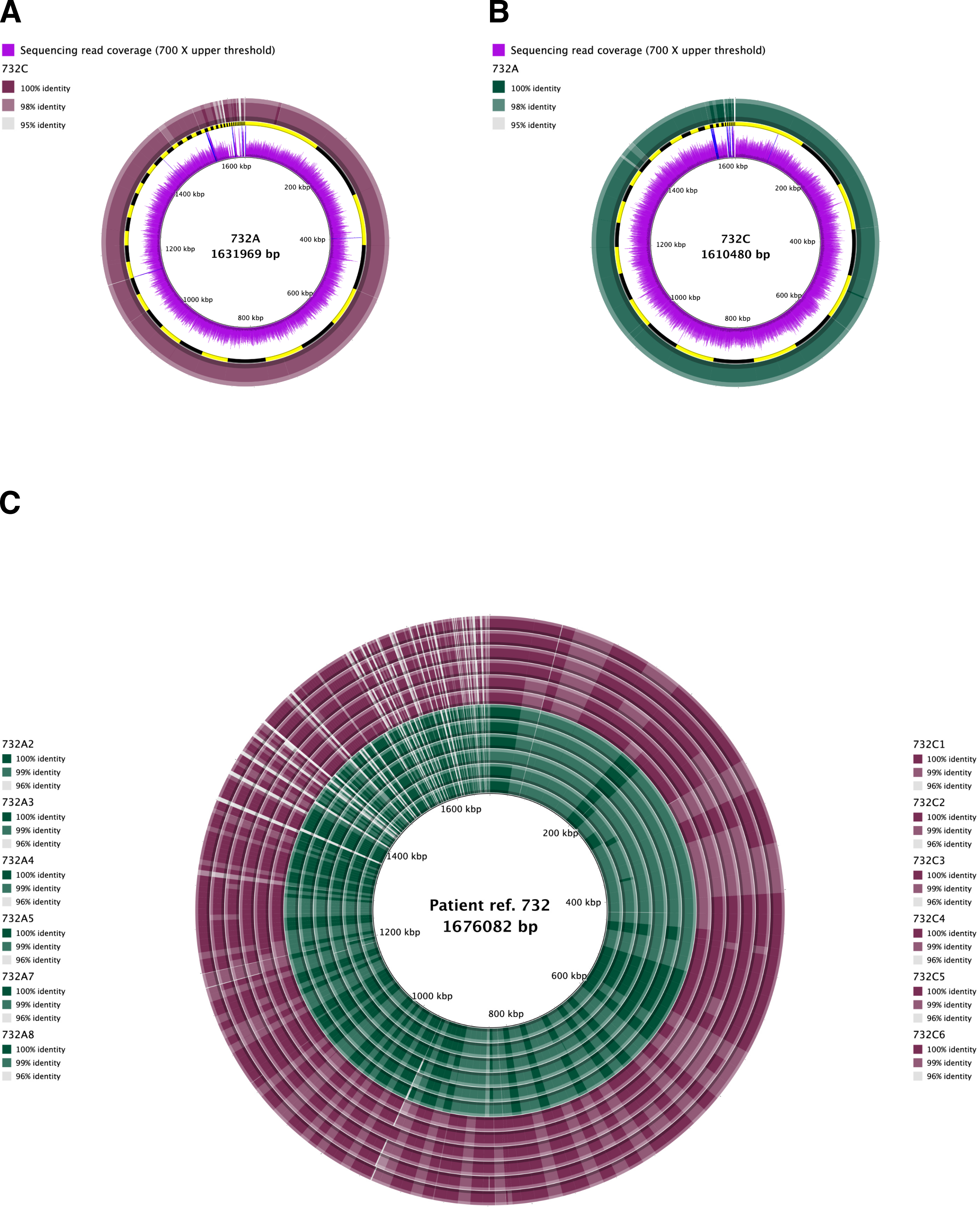
A
Sequencing read coverage (700 X upper threshold)
732C
100% identity
98% identity
95% identity
1600 kbp
200 kbp
1400 kbp
400 kbp
1200 kbp
732A
1631969 bp
1000 kbp
600 kbp
800 kbp
B
Sequencing read coverage (700 X upper threshold)
732A
100% identity
98% identity
95% identity
1600 kbp
200 kbp
1400 kbp
400 kbp
1200 kbp
732C
1610480 bp
1000 kbp
600 kbp
800 kbp
C
732A2
100% identity
99% identity
96% identity
732A3
100% identity
99% identity
96% identity
732A4
100% identity
99% identity
96% identity
732A5
100% identity
99% identity
96% identity
732A7
100% identity
99% identity
96% identity
732A8
100% identity
99% identity
96% identity
1600 kbp
200 kbp
1400 kbp
400 kbp
Patient ref. 732
1676082 bp
1200 kbp
600 kbp
1000 kbp
800 kbp
732C1
100% identity
99% identity
96% identity
732C2
100% identity
99% identity
96% identity
732C3
100% identity
99% identity
96% identity
732C4
100% identity
99% identity
96% identity
732C5
100% identity
99% identity
96% identity
732C6
100% identity
99% identity
96% identity
