# Supplementary material for: Genomic diversity of Helicobacter pylori populations from different regions of the human stomach
Source: Gut Microbes. 2022 Dec 5;14(1):2152306. doi: 10.1080/19490976.2022.2152306 (PMC9728471; doi:10.1080/19490976.2022.2152306)
Supplement: Supplemental Material [file KGMI_A_2152306_SM1608.zip › SupplFig15.pdf]

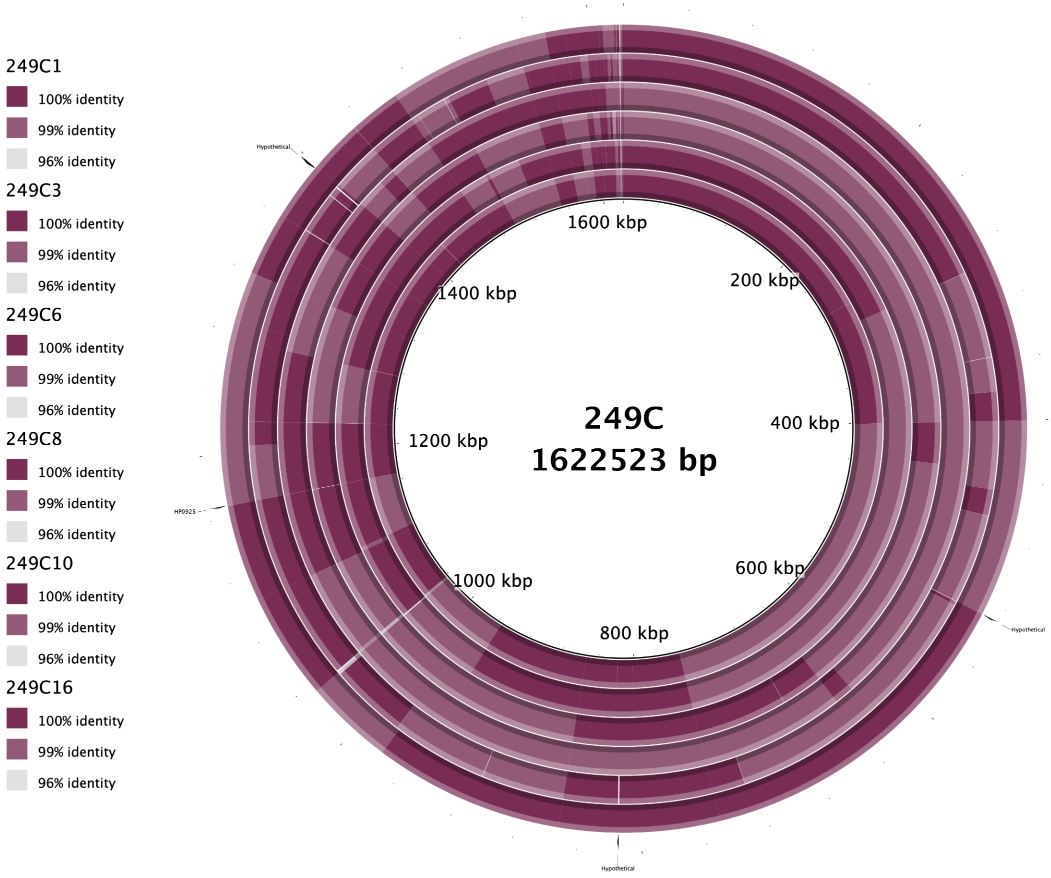
249C1
100% identity
99% identity
96% identity
249C3
100% identity
99% identity
96% identity
249C6
100% identity
99% identity
96% identity
249C8
100% identity
99% identity
96% identity
249C10
100% identity
99% identity
96% identity
249C16
100% identity
99% identity
96% identity
249C
1622523 bp
1600 kbp
200 kbp
400 kbp
600 kbp
800 kbp
1000 kbp
1200 kbp
1400 kbp
Hypothetical
HP0925
Hypothetical
Hypothetical
