# Supplementary material for: Genomic diversity of Helicobacter pylori populations from different regions of the human stomach
Source: Gut Microbes. 2022 Dec 5;14(1):2152306. doi: 10.1080/19490976.2022.2152306 (PMC9728471; doi:10.1080/19490976.2022.2152306)
Supplement: Supplemental Material [file KGMI_A_2152306_SM1608.zip › SupplFig23.pdf]

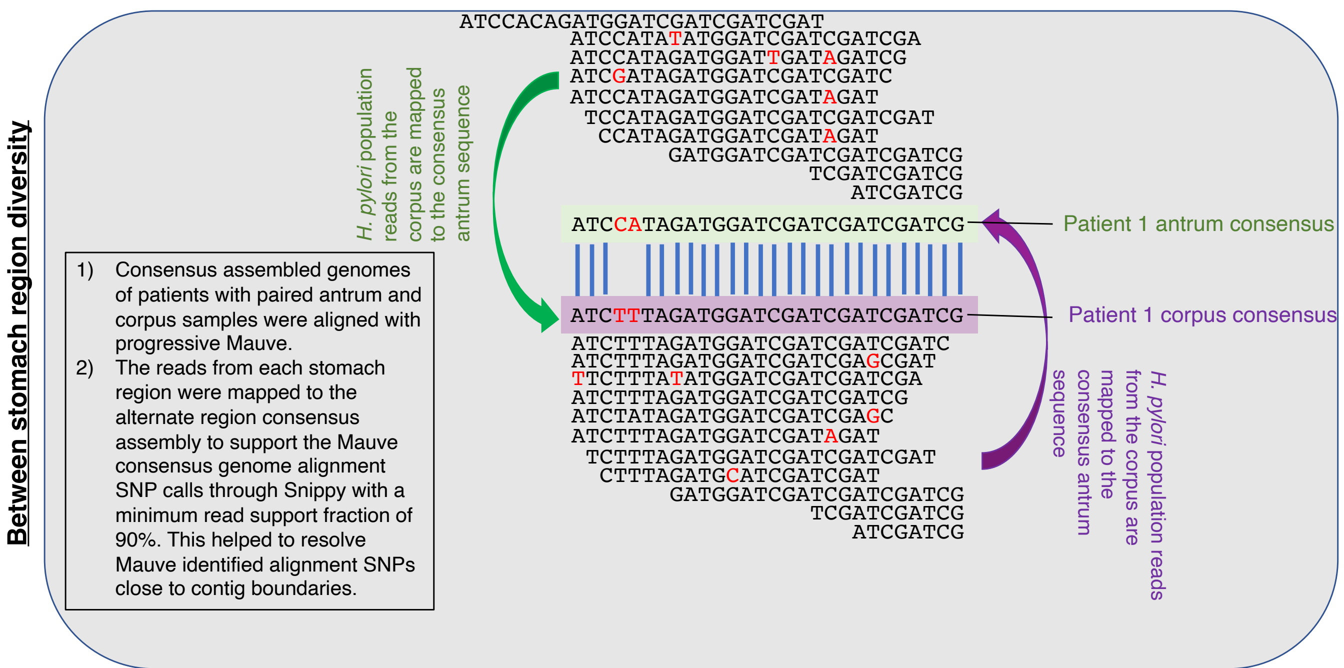

Between stomach region diversity
ATCCACAGATGGATCGATCGATCGAT
ATCCATATATGGATCGATCGATCGA
ATCCATAGATGGATTGATAGATCG
ATCGATAGATGGATCGATCGATC
ATCCATAGATGGATCGATAGAT
TCCATAGATGGATCGATCGATCGAT
CCATAGATGGATCGATAGAT
GATGGATCGATCGATCGATCG
TCGATCGATCG
ATCGATCG
H. pylori population reads from the corpus are mapped to the consensus antrum sequence
ATCCATAGATGGATCGATCGATCGATCG
Patient 1 antrum consensus
ATCTTTAGATGGATCGATCGATCGATCG
Patient 1 corpus consensus
ATCTTTAGATGGATCGATCGATCGATC
ATCTTTAGATGGATCGATCGAGCGAT
TTCTTTATATGGATCGATCGATCGA
ATCTTTAGATGGATCGATCGATCG
ATCTATAGATGGATCGATCGAGC
ATCTTTAGATGGATCGATAGAT
TCTTTAGATGGATCGATCGATCGAT
CTTTAGATGCATCGATCGAT
GATGGATCGATCGATCGATCG
TCGATCGATCG
ATCGATCG
H. pylori population reads from the corpus are mapped to the consensus antrum sequence
1) Consensus assembled genomes of patients with paired antrum and corpus samples were aligned with progressive Mauve.
2) The reads from each stomach region were mapped to the alternate region consensus assembly to support the Mauve consensus genome alignment SNP calls through Snippy with a minimum read support fraction of 90%. This helped to resolve Mauve identified alignment SNPs close to contig boundaries.
