# Supplementary material for: Genomic diversity of Helicobacter pylori populations from different regions of the human stomach
Source: Gut Microbes. 2022 Dec 5;14(1):2152306. doi: 10.1080/19490976.2022.2152306 (PMC9728471; doi:10.1080/19490976.2022.2152306)
Supplement: Supplemental Material [file KGMI_A_2152306_SM1608.zip › SupplFig4.pdf]

A

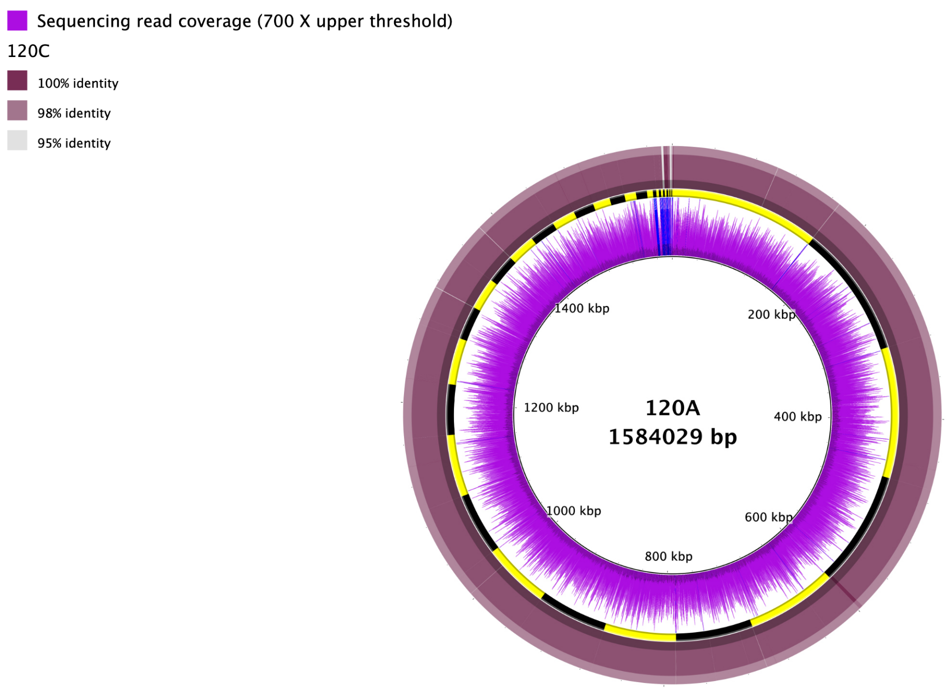

Sequencing read coverage (700 X upper threshold)
120C
100% identity
98% identity
95% identity
1400 kbp
200 kbp
1200 kbp
120A
1584029 bp
400 kbp
1000 kbp
600 kbp
800 kbp

B

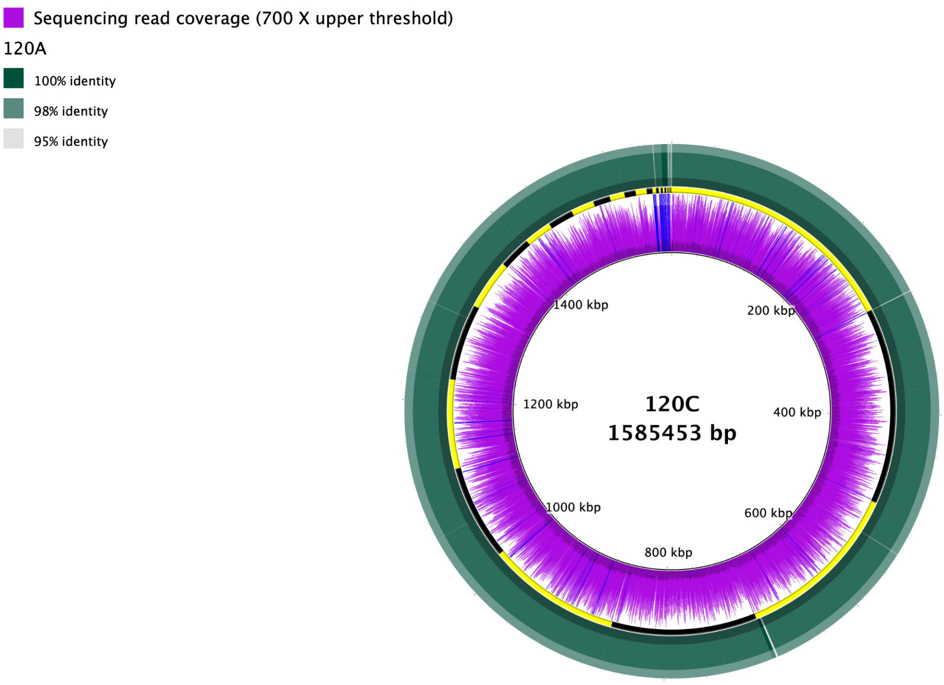

Sequencing read coverage (700 X upper threshold)
120A
100% identity
98% identity
95% identity
1400 kbp
200 kbp
1200 kbp
120C
1585453 bp
400 kbp
1000 kbp
600 kbp
800 kbp
