# Supplementary material for: Genomic diversity of Helicobacter pylori populations from different regions of the human stomach
Source: Gut Microbes. 2022 Dec 5;14(1):2152306. doi: 10.1080/19490976.2022.2152306 (PMC9728471; doi:10.1080/19490976.2022.2152306)
Supplement: Supplemental Material [file KGMI_A_2152306_SM1608.zip › SupplFig5.pdf]

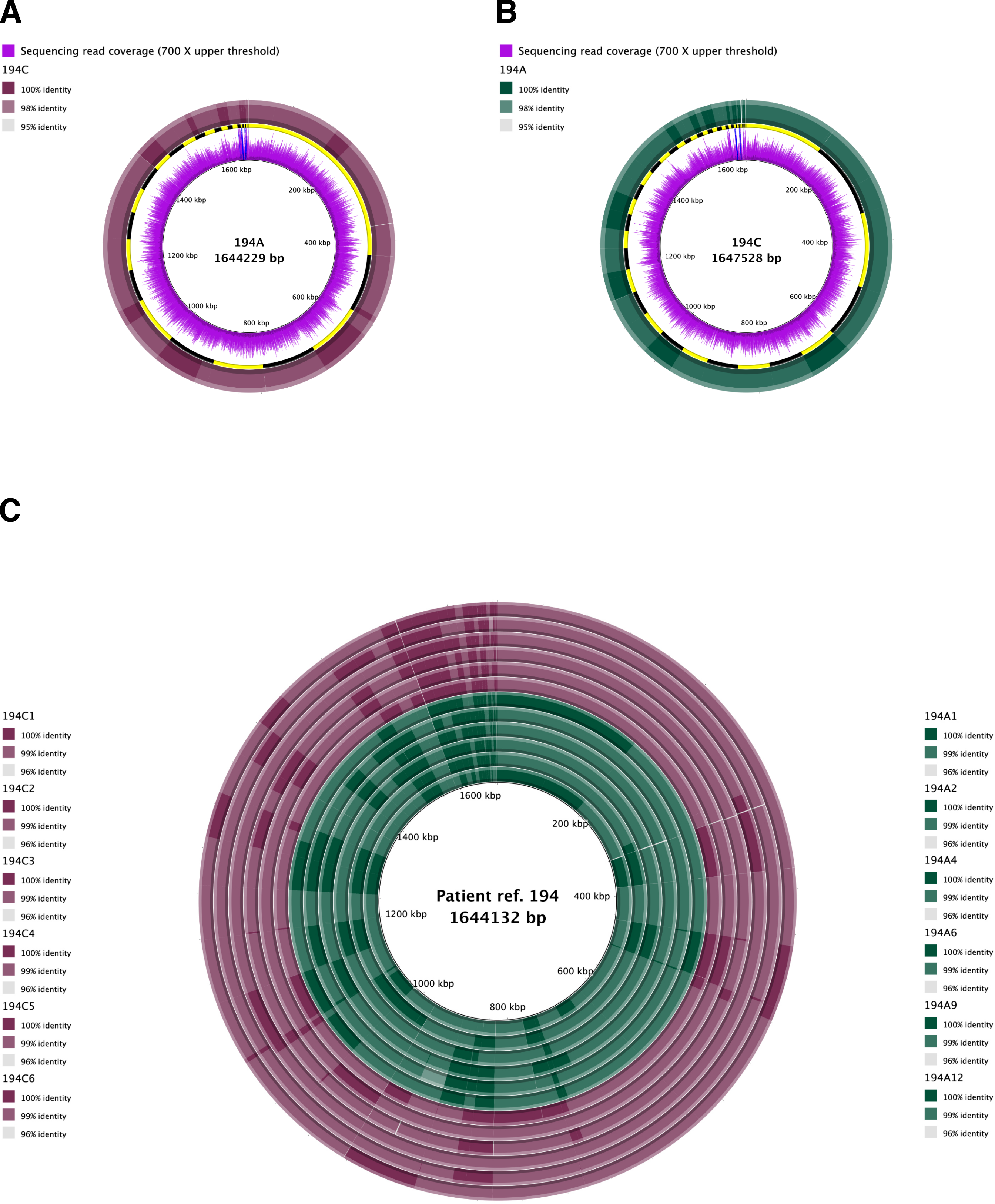
A
Sequencing read coverage (700 X upper threshold)
194C
100% identity
98% identity
95% identity
1600 kbp
200 kbp
400 kbp
600 kbp
800 kbp
1000 kbp
1200 kbp
1400 kbp
194A
1644229 bp
B
Sequencing read coverage (700 X upper threshold)
194A
100% identity
98% identity
95% identity
1600 kbp
200 kbp
400 kbp
600 kbp
800 kbp
1000 kbp
1200 kbp
1400 kbp
194C
1647528 bp
C
194C1
100% identity
99% identity
96% identity
194C2
100% identity
99% identity
96% identity
194C3
100% identity
99% identity
96% identity
194C4
100% identity
99% identity
96% identity
194C5
100% identity
99% identity
96% identity
194C6
100% identity
99% identity
96% identity
1600 kbp
200 kbp
400 kbp
600 kbp
800 kbp
1000 kbp
1200 kbp
1400 kbp
Patient ref. 194
1644132 bp
194A1
100% identity
99% identity
96% identity
194A2
100% identity
99% identity
96% identity
194A4
100% identity
99% identity
96% identity
194A6
100% identity
99% identity
96% identity
194A9
100% identity
99% identity
96% identity
194A12
100% identity
99% identity
96% identity
