# Supplementary material for: Genomic diversity of Helicobacter pylori populations from different regions of the human stomach
Source: Gut Microbes. 2022 Dec 5;14(1):2152306. doi: 10.1080/19490976.2022.2152306 (PMC9728471; doi:10.1080/19490976.2022.2152306)
Supplement: Supplemental Material [file KGMI_A_2152306_SM1608.zip › SupplFig7.pdf]

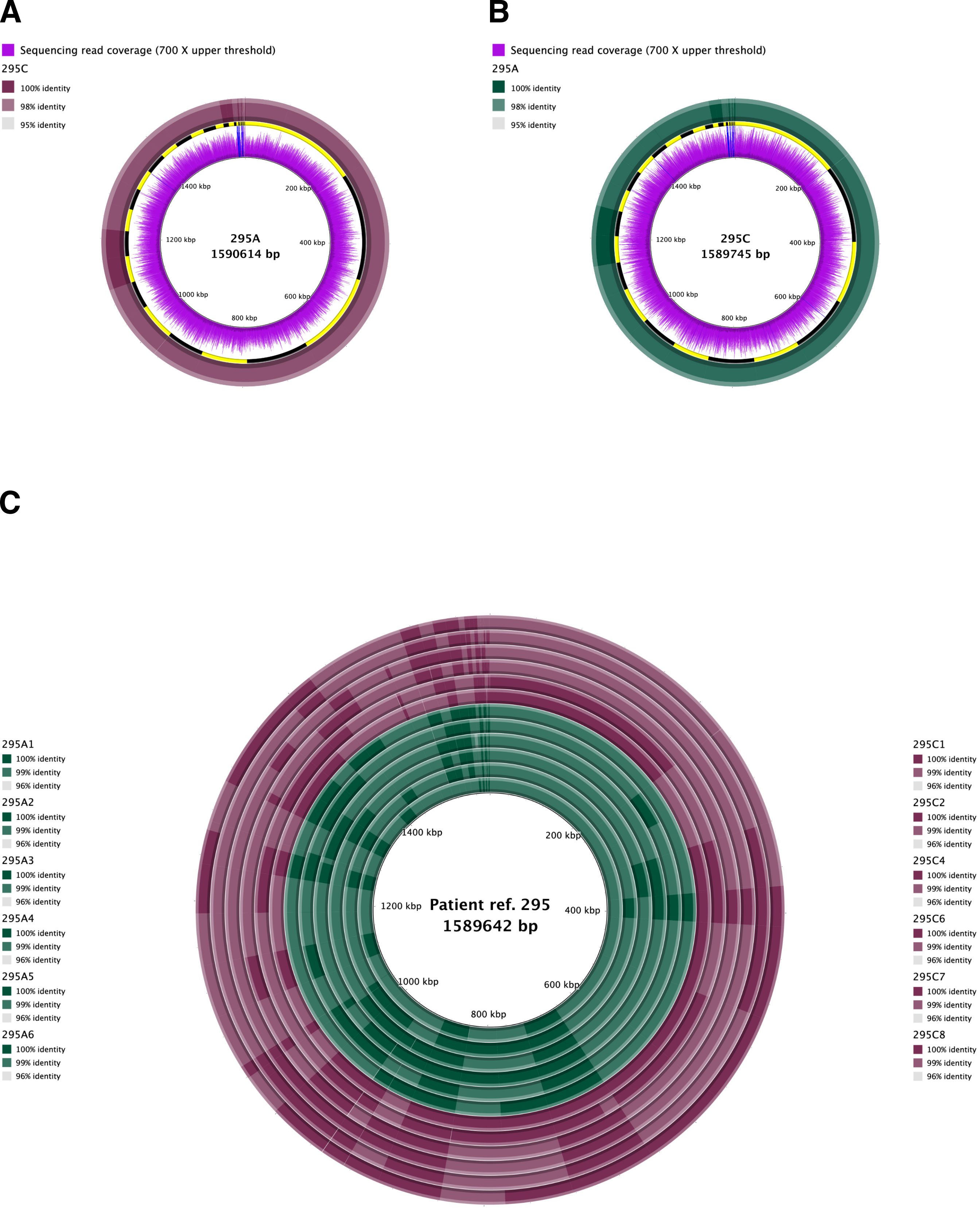
A
Sequencing read coverage (700 X upper threshold)
295C
100% identity
98% identity
95% identity
295A
1590614 bp
200 kbp
400 kbp
600 kbp
800 kbp
1000 kbp
1200 kbp
1400 kbp
B
Sequencing read coverage (700 X upper threshold)
295A
100% identity
98% identity
95% identity
295C
1589745 bp
200 kbp
400 kbp
600 kbp
800 kbp
1000 kbp
1200 kbp
1400 kbp
C
295A1
100% identity
99% identity
96% identity
295A2
100% identity
99% identity
96% identity
295A3
100% identity
99% identity
96% identity
295A4
100% identity
99% identity
96% identity
295A5
100% identity
99% identity
96% identity
295A6
100% identity
99% identity
96% identity
Patient ref. 295
1589642 bp
200 kbp
400 kbp
600 kbp
800 kbp
1000 kbp
1200 kbp
1400 kbp
295C1
100% identity
99% identity
96% identity
295C2
100% identity
99% identity
96% identity
295C4
100% identity
99% identity
96% identity
295C6
100% identity
99% identity
96% identity
295C7
100% identity
99% identity
96% identity
295C8
100% identity
99% identity
96% identity
