# Supplementary material for: Genomic diversity of Helicobacter pylori populations from different regions of the human stomach
Source: Gut Microbes. 2022 Dec 5;14(1):2152306. doi: 10.1080/19490976.2022.2152306 (PMC9728471; doi:10.1080/19490976.2022.2152306)
Supplement: Supplemental Material [file KGMI_A_2152306_SM1608.zip › SupplFig8.pdf]

A

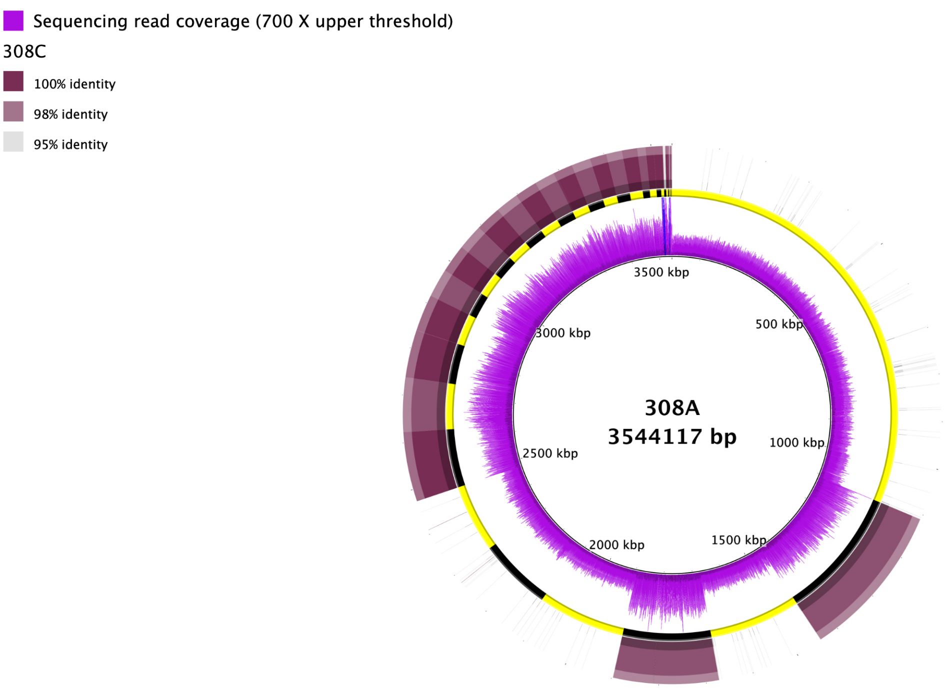
Sequencing read coverage (700 X upper threshold)
308C
100% identity
98% identity
95% identity
3500 kbp
500 kbp
3000 kbp
308A
3544117 bp
1000 kbp
2500 kbp
2000 kbp
1500 kbp

B

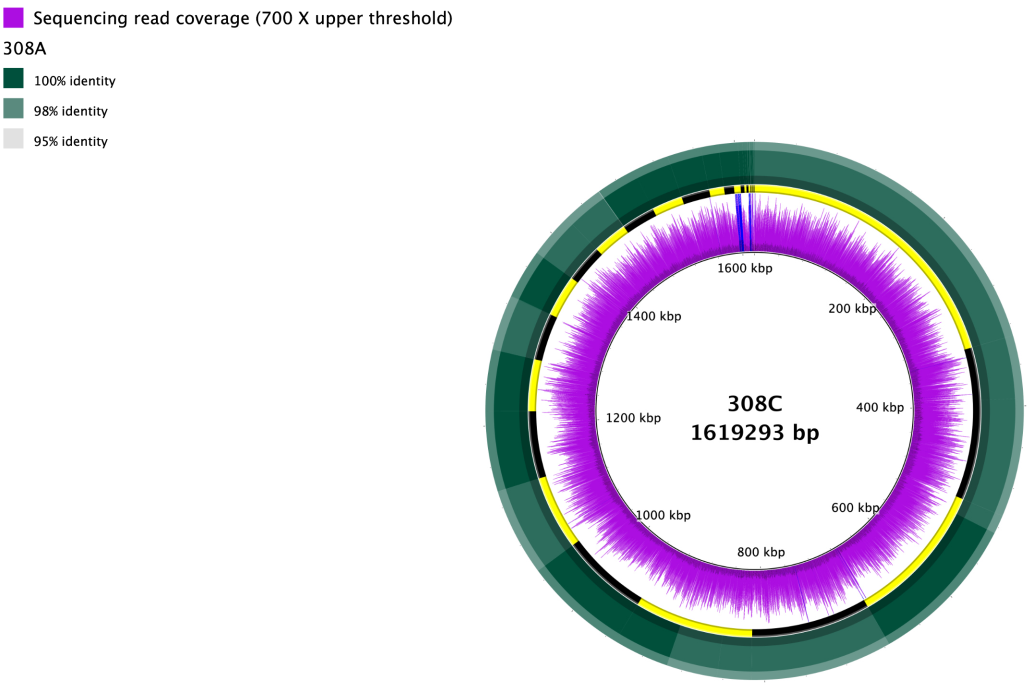
Sequencing read coverage (700 X upper threshold)
308A
100% identity
98% identity
95% identity
1600 kbp
200 kbp
1400 kbp
308C
1619293 bp
400 kbp
1200 kbp
1000 kbp
600 kbp
800 kbp
