# Supplementary material for: Genomic diversity of Helicobacter pylori populations from different regions of the human stomach
Source: Gut Microbes. 2022 Dec 5;14(1):2152306. doi: 10.1080/19490976.2022.2152306 (PMC9728471; doi:10.1080/19490976.2022.2152306)
Supplement: Supplemental Material [file KGMI_A_2152306_SM1608.zip › SupplFig9.pdf]

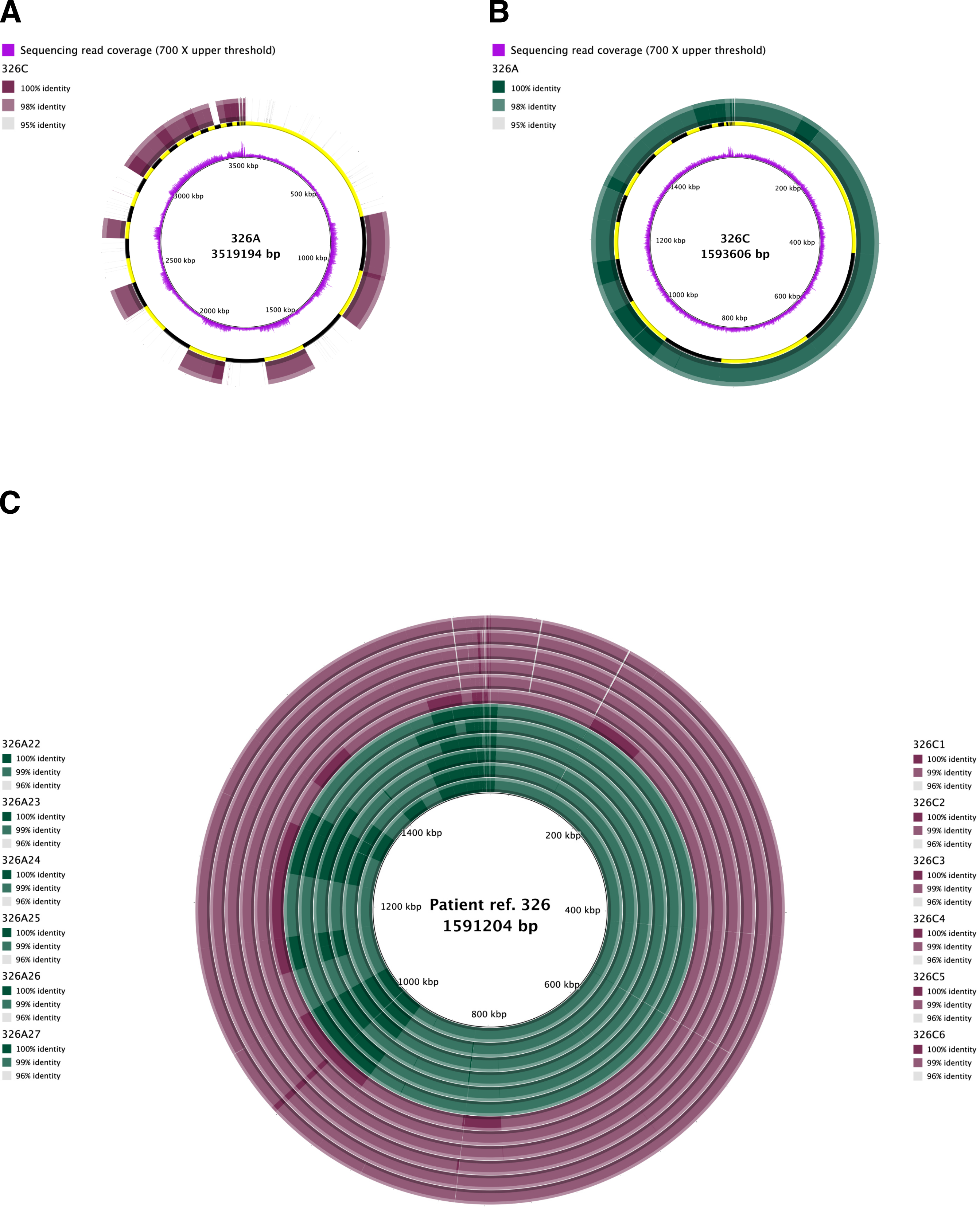

A
Sequencing read coverage (700 X upper threshold)
326C
100% identity
98% identity
95% identity
326A
3519194 bp
3500 kbp
500 kbp
3000 kbp
1000 kbp
2500 kbp
2000 kbp
1500 kbp
B
Sequencing read coverage (700 X upper threshold)
326A
100% identity
98% identity
95% identity
326C
1593606 bp
1400 kbp
200 kbp
1200 kbp
400 kbp
1000 kbp
600 kbp
800 kbp
C
326A22
100% identity
99% identity
96% identity
326A23
100% identity
99% identity
96% identity
326A24
100% identity
99% identity
96% identity
326A25
100% identity
99% identity
96% identity
326A26
100% identity
99% identity
96% identity
326A27
100% identity
99% identity
96% identity
Patient ref. 326
1591204 bp
1400 kbp
200 kbp
1200 kbp
400 kbp
1000 kbp
600 kbp
800 kbp
326C1
100% identity
99% identity
96% identity
326C2
100% identity
99% identity
96% identity
326C3
100% identity
99% identity
96% identity
326C4
100% identity
99% identity
96% identity
326C5
100% identity
99% identity
96% identity
326C6
100% identity
99% identity
96% identity
