# Supplementary figures and images for: Genomic diversity of Helicobacter pylori populations from different regions of the human stomach
Source: Gut Microbes. 2022 Dec 5;14(1):2152306. doi: 10.1080/19490976.2022.2152306 (PMC9728471; doi:10.1080/19490976.2022.2152306)

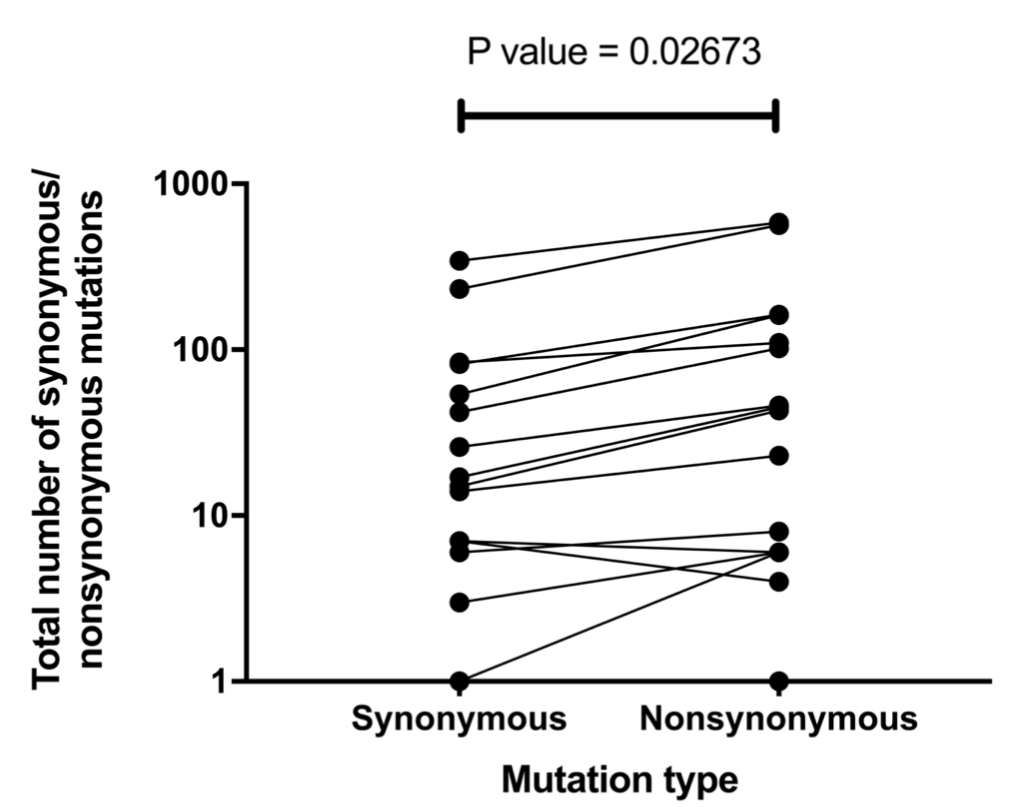

Supplement: Supplemental Material [file KGMI_A_2152306_SM1608.zip › SupplFig17.png]

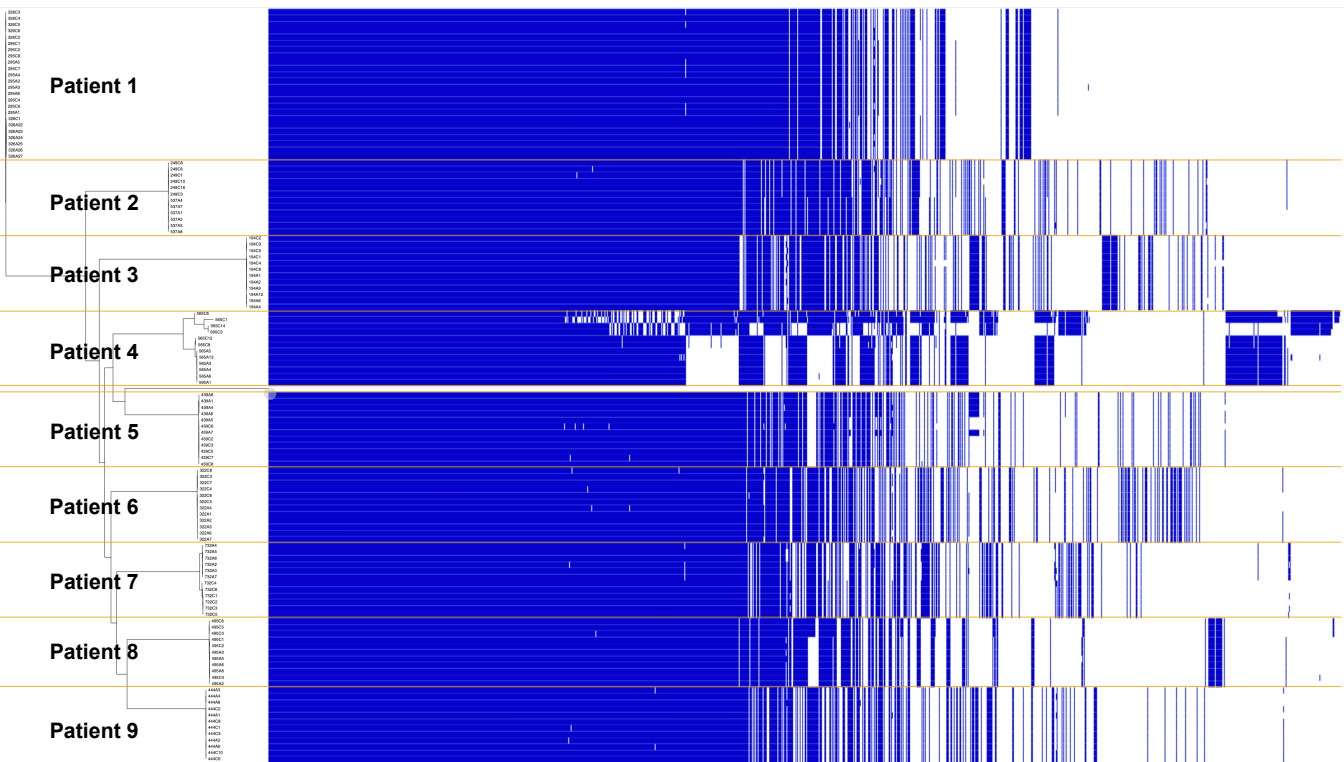

Supplement: Supplemental Material [file KGMI_A_2152306_SM1608.zip › SupplFig18.pdf]

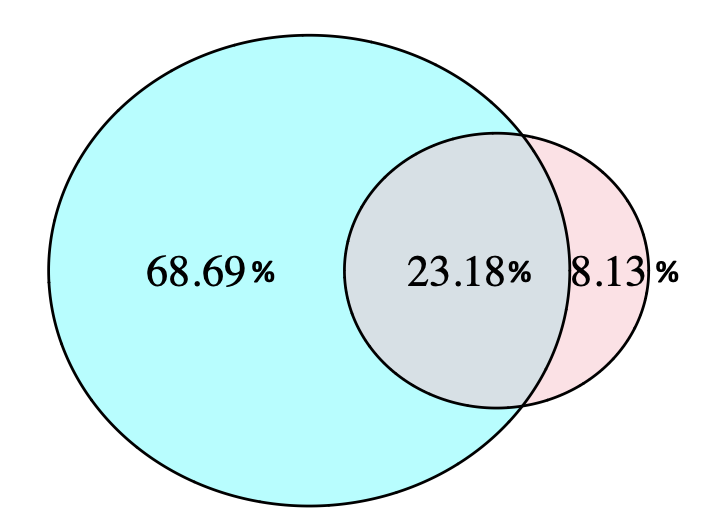

Supplement: Supplemental Material [file KGMI_A_2152306_SM1608.zip › SupplFig22.png]
