# Supplementary material for: Genomic diversity of Helicobacter pylori populations from different regions of the human stomach
Source: Gut Microbes. 2022 Dec 5;14(1):2152306. doi: 10.1080/19490976.2022.2152306 (PMC9728471; doi:10.1080/19490976.2022.2152306)
Supplement: Supplemental Material [file SupplFig12.pdf › SupplFig12.pdf]

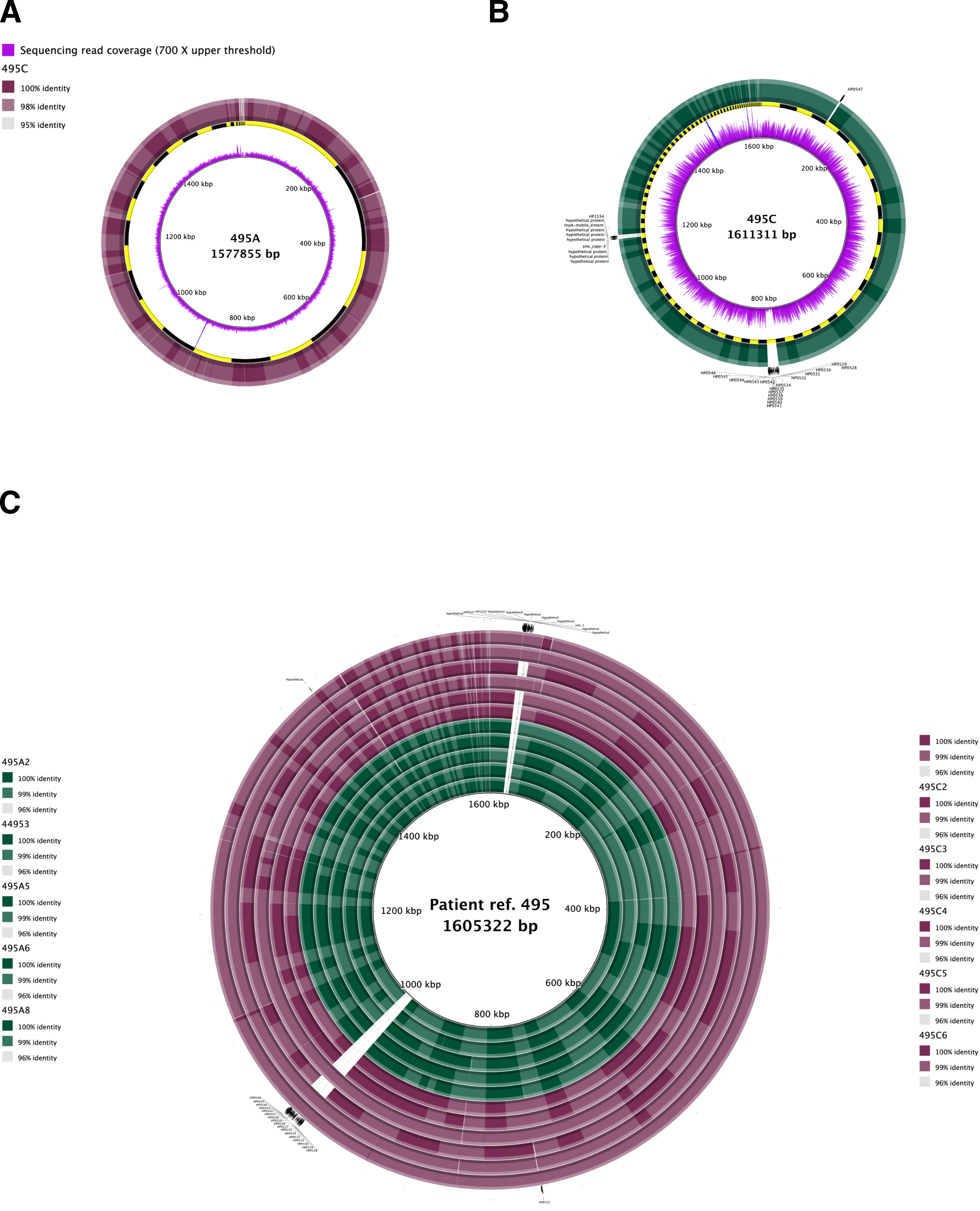
A
Sequencing read coverage (700 X upper threshold)
495C
100% identity
98% identity
95% identity
1400 kbp
200 kbp
1200 kbp
495A
1577855 bp
400 kbp
1000 kbp
600 kbp
800 kbp
B
HP0547
1600 kbp
1400 kbp
200 kbp
1200 kbp
495C
1611311 bp
400 kbp
1000 kbp
600 kbp
800 kbp
HP1534
hypothetical protein
tnpA-mobile_elment
hypothetical protein
hypothetical protein
hypothetical protein
smc_copy-3
hypothetical protein
hypothetical protein
hypothetical protein
HP0546
HP0545
HP0544
HP0543
HP0542
HP0534
HP0535
HP0537
HP0538
HP0539
HP0540
HP0541
HP0532
HP0531
HP0530
HP0529
HP0528
C
495A2
100% identity
99% identity
96% identity
44953
100% identity
99% identity
96% identity
495A5
100% identity
99% identity
96% identity
495A6
100% identity
99% identity
96% identity
495A8
100% identity
99% identity
96% identity
1600 kbp
1400 kbp
200 kbp
1200 kbp
Patient ref. 495
1605322 bp
400 kbp
1000 kbp
600 kbp
800 kbp
HP0722
100% identity
99% identity
96% identity
495C2
100% identity
99% identity
96% identity
495C3
100% identity
99% identity
96% identity
495C4
100% identity
99% identity
96% identity
495C5
100% identity
99% identity
96% identity
495C6
100% identity
99% identity
96% identity
