# Supplementary material for: Genomic diversity of Helicobacter pylori populations from different regions of the human stomach
Source: Gut Microbes. 2022 Dec 5;14(1):2152306. doi: 10.1080/19490976.2022.2152306 (PMC9728471; doi:10.1080/19490976.2022.2152306)
Supplement: Supplemental Material [file SupplFig21.pdf › SupplFig21.pdf]

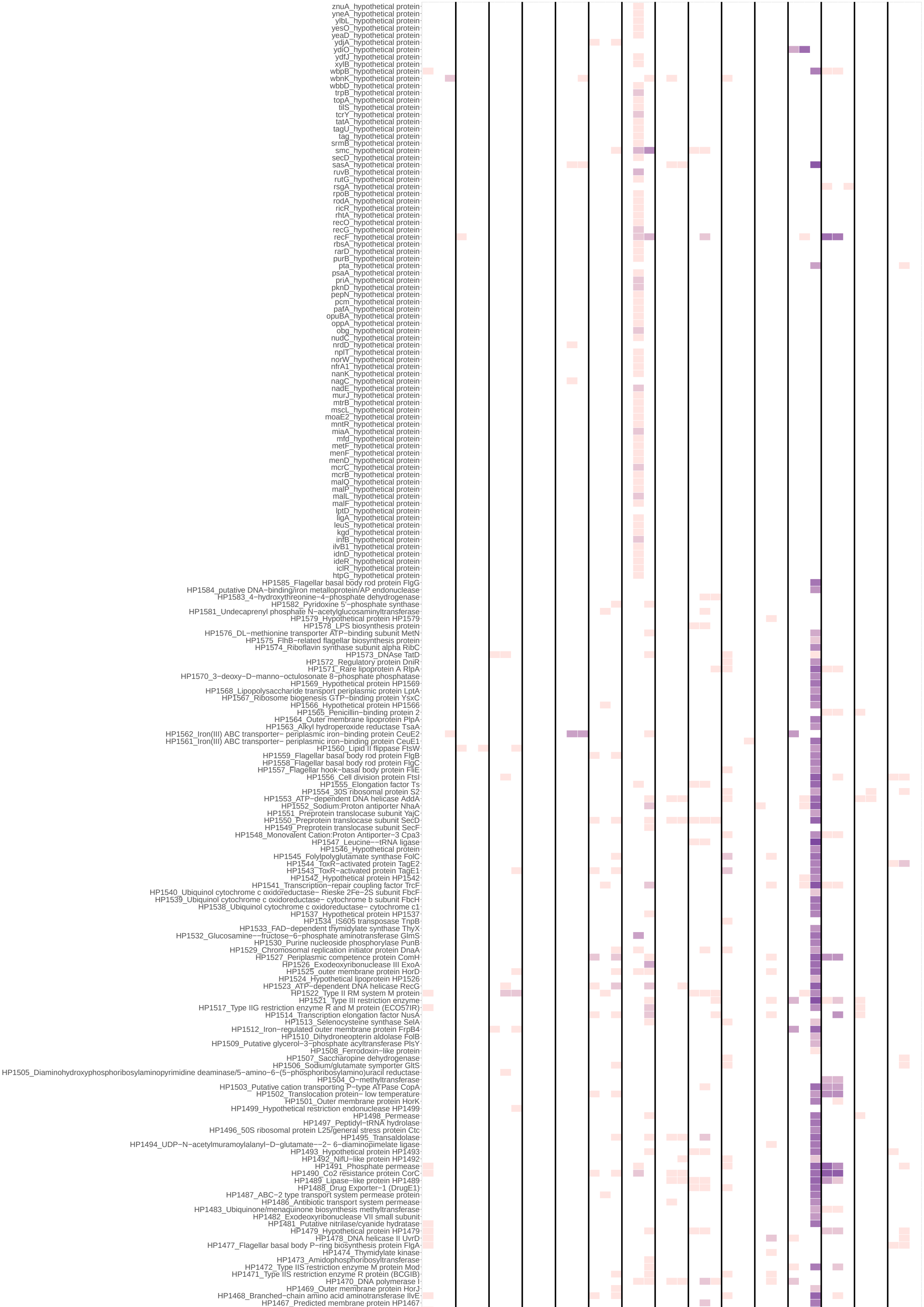
znuA_hypothetical protein
yneA_hypothetical protein
ylbL_hypothetical protein
yesO_hypothetical protein
yeaD_hypothetical protein
ydjA_hypothetical protein
ydiO_hypothetical protein
ydfJ_hypothetical protein
xylB_hypothetical protein
wbpB_hypothetical protein
wbnK_hypothetical protein
wbbD_hypothetical protein
trpB_hypothetical protein
topA_hypothetical protein
tilS_hypothetical protein
tcrY_hypothetical protein
tatA_hypothetical protein
tagU_hypothetical protein
tag_hypothetical protein
srmB_hypothetical protein
smc_hypothetical protein
secD_hypothetical protein
sasA_hypothetical protein
ruvB_hypothetical protein
rutG_hypothetical protein
rsgA_hypothetical protein
rpoB_hypothetical protein
rodA_hypothetical protein
ricR_hypothetical protein
rhtA_hypothetical protein
recO_hypothetical protein
recG_hypothetical protein
recF_hypothetical protein
rbsA_hypothetical protein
rarD_hypothetical protein
purB_hypothetical protein
pta_hypothetical protein
psaA_hypothetical protein
priA_hypothetical protein
pknD_hypothetical protein
pepN_hypothetical protein
pcm_hypothetical protein
pafA_hypothetical protein
opuBA_hypothetical protein
oppA_hypothetical protein
obg_hypothetical protein
nudC_hypothetical protein
nrdD_hypothetical protein
nplT_hypothetical protein
norW_hypothetical protein
nfrA1_hypothetical protein
nanK_hypothetical protein
nagC_hypothetical protein
nadE_hypothetical protein
murJ_hypothetical protein
mtrB_hypothetical protein
mscL_hypothetical protein
moaE2_hypothetical protein
mntR_hypothetical protein
miaA_hypothetical protein
mfd_hypothetical protein
metF_hypothetical protein
menF_hypothetical protein
menD_hypothetical protein
mcrC_hypothetical protein
mcrB_hypothetical protein
malQ_hypothetical protein
malP_hypothetical protein
malL_hypothetical protein
malF_hypothetical protein
lptD_hypothetical protein
ligA_hypothetical protein
leuS_hypothetical protein
kgd_hypothetical protein
infB_hypothetical protein
ilvB1_hypothetical protein
idnD_hypothetical protein
ideR_hypothetical protein
iclR_hypothetical protein
htpG_hypothetical protein
HP1585_Flagellar basal body rod protein FlgG
HP1584_putative DNA−binding/iron metalloprotein/AP endonuclease
HP1583_4−hydroxythreonine−4−phosphate dehydrogenase
HP1582_Pyridoxine 5'−phosphate synthase
HP1581_Undecaprenyl phosphate N−acetylglucosaminyltransferase
HP1579_Hypothetical protein HP1579
HP1578_LPS biosynthesis protein
HP1576_DL−methionine transporter ATP−binding subunit MetN
HP1575_FlhB−related flagellar biosynthesis protein
HP1574_Riboflavin synthase subunit alpha RibC
HP1573_DNAse TatD
HP1572_Regulatory protein DniR
HP1571_Rare lipoprotein A RlpA
HP1570_3−deoxy−D−manno−octulosonate 8−phosphate phosphatase
HP1569_Hypothetical protein HP1569
HP1568_Lipopolysaccharide transport periplasmic protein LptA
HP1567_Ribosome biogenesis GTP−binding protein YsxC
HP1566_Hypothetical protein HP1566
HP1565_Penicillin−binding protein 2
HP1564_Outer membrane lipoprotein PlpA
HP1563_Alkyl hydroperoxide reductase TsaA
HP1562_Iron(III) ABC transporter− periplasmic iron−binding protein CeuE2
HP1561_Iron(III) ABC transporter− periplasmic iron−binding protein CeuE1
HP1560_Lipid II flippase FtsW
HP1559_Flagellar basal body rod protein FlgB
HP1558_Flagellar basal body rod protein FlgC
HP1557_Flagellar hook−basal body protein FliE
HP1556_Cell division protein FtsI
HP1555_Elongation factor Ts
HP1554_30S ribosomal protein S2
HP1553_ATP−dependent DNA helicase AddA
HP1552_Sodium:Proton antiporter NhaA
HP1551_Preprotein translocase subunit YajC
HP1550_Preprotein translocase subunit SecD
HP1549_Preprotein translocase subunit SecF
HP1548_Monovalent Cation:Proton Antiporter−3 Cpa3
HP1547_Leucine−−tRNA ligase
HP1546_Hypothetical protein
HP1545_Folylpolyglutamate synthase FolC
HP1544_ToxR−activated protein TagE2
HP1543_ToxR−activated protein TagE1
HP1542_Hypothetical protein HP1542
HP1541_Transcription−repair coupling factor TrcF
HP1540_Ubiquinol cytochrome c oxidoreductase− Rieske 2Fe−2S subunit FbcF
HP1539_Ubiquinol cytochrome c oxidoreductase− cytochrome b subunit FbcH
HP1538_Ubiquinol cytochrome c oxidoreductase− cytochrome c1
HP1537_Hypothetical protein HP1537
HP1534_IS605 transposase TnpB
HP1533_FAD−dependent thymidylate synthase ThyX
HP1532_Glucosamine−−fructose−6−phosphate aminotransferase GlmS
HP1530_Purine nucleoside phosphorylase PunB
HP1529_Chromosomal replication initiator protein DnaA
HP1527_Periplasmic competence protein ComH
HP1526_Exodeoxyribonuclease III ExoA
HP1525_outer membrane protein HorD
HP1524_Hypothetical lipoprotein HP1526
HP1523_ATP−dependent DNA helicase RecG
HP1522_Type II RM system M protein
HP1521_Type III restriction enzyme
HP1517_Type IIG restriction enzyme R and M protein (ECO57IR)
HP1514_Transcription elongation factor NusA
HP1513_Selenocysteine synthase SelA
HP1512_Iron−regulated outer membrane protein FrpB4
HP1510_Dihydroneopterin aldolase FolB
HP1509_Putative glycerol−3−phosphate acyltransferase PlsY
HP1508_Ferrodoxin−like protein
HP1507_Saccharopine dehydrogenase
HP1506_Sodium/glutamate symporter GltS
HP1505_Diaminohydroxyphosphoribosylaminopyrimidine deaminase/5−amino−6−(5−phosphoribosylamino)uracil reductase
HP1504_O−methyltransferase
HP1503_Putative cation transporting P−type ATPase CopA
HP1502_Translocation protein− low temperature
HP1501_Outer membrane protein HorK
HP1499_Hypothetical restriction endonuclease HP1499
HP1498_Permease
HP1497_Peptidyl−tRNA hydrolase
HP1496_50S ribosomal protein L25/general stress protein Ctc
HP1495_Transaldolase
HP1494_UDP−N−acetylmuramoylalanyl−D−glutamate−−2− 6−diaminopimelate ligase
HP1493_Hypothetical protein HP1493
HP1492_NifU−like protein HP1492
HP1491_Phosphate permease
HP1490_Co2 resistance protein CorC
HP1489_Lipase−like protein HP1489
HP1488_Drug Exporter−1 (DrugE1)
HP1487_ABC−2 type transport system permease protein
HP1486_Antibiotic transport system permease
HP1483_Ubiquinone/menaquinone biosynthesis methyltransferase
HP1482_Exodeoxyribonuclease VII small subunit
HP1481_Putative nitrilase/cyanide hydratase
HP1479_Hypothetical protein HP1479
HP1478_DNA helicase II UvrD
HP1477_Flagellar basal body P−ring biosynthesis protein FlgA
HP1474_Thymidylate kinase
HP1473_Amidophosphoribosyltransferase
HP1472_Type IIS restriction enzyme M protein Mod
HP1471_Type IIS restriction enzyme R protein (BCGIB)
HP1470_DNA polymerase I
HP1469_Outer membrane protein HorJ
HP1468_Branched−chain amino acid aminotransferase IlvE
HP1467_Predicted membrane protein HP1467

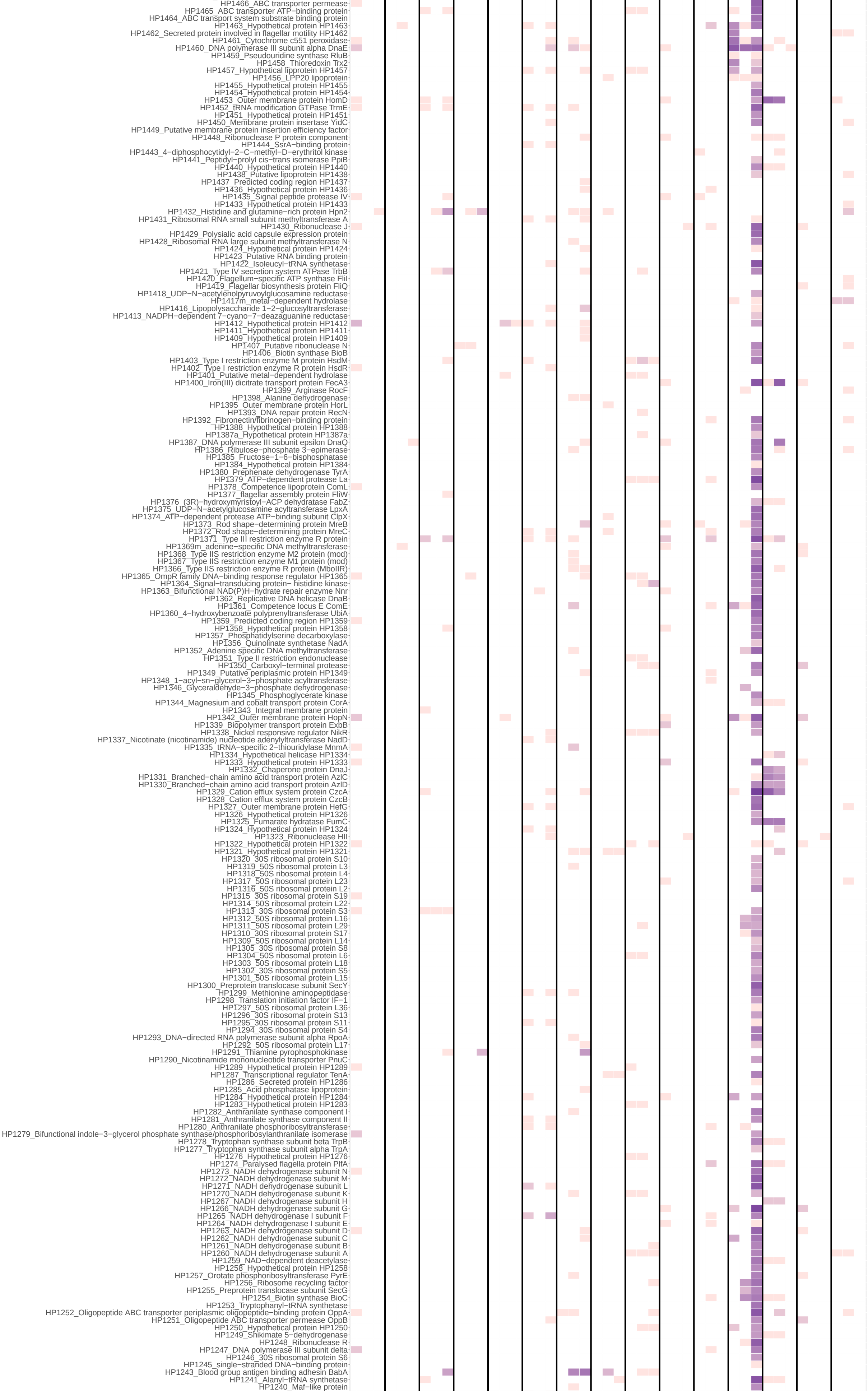
HP1466_ABC transporter permease
HP1465_ABC transporter ATP−binding protein
HP1464_ABC transport system substrate binding protein
HP1463_Hypothetical protein HP1463
HP1462_Secreted protein involved in flagellar motility HP1462
HP1461_Cytochrome c551 peroxidase
HP1460_DNA polymerase III subunit alpha DnaE
HP1459_Pseudouridine synthase RluB
HP1458_Thioredoxin Trx2
HP1457_Hypothetical lipprotein HP1457
HP1456_LPP20 lipoprotein
HP1455_Hypothetical protein HP1455
HP1454_Hypothetical protein HP1454
HP1453_Outer membrane protein HomD
HP1452_tRNA modification GTPase TrmE
HP1451_Hypothetical protein HP1451
HP1450_Membrane protein insertase YidC
HP1449_Putative membrane protein insertion efficiency factor
HP1448_Ribonuclease P protein component
HP1444_SsrA−binding protein
HP1443_4−diphosphocytidyl−2−C−methyl−D−erythritol kinase
HP1441_Peptidyl−prolyl cis−trans isomerase PpiB
HP1440_Hypothetical protein HP1440
HP1438_Putative lipoprotein HP1438
HP1437_Predicted coding region HP1437
HP1436_Hypothetical protein HP1436
HP1435_Signal peptide protease IV
HP1433_Hypothetical protein HP1433
HP1432_Histidine and glutamine−rich protein Hpn2
HP1431_Ribosomal RNA small subunit methyltransferase A
HP1430_Ribonuclease J
HP1429_Polysialic acid capsule expression protein
HP1428_Ribosomal RNA large subunit methyltransferase N
HP1424_Hypothetical protein HP1424
HP1423_Putative RNA binding protein
HP1422_Isoleucyl−tRNA synthetase
HP1421_Type IV secretion system ATPase TrbB
HP1420_Flagellum−specific ATP synthase FliI
HP1419_Flagellar biosynthesis protein FliQ
HP1418_UDP−N−acetylenolpyruvoylglucosamine reductase
HP1417m_metal−dependent hydrolase
HP1416_Lipopolysaccharide 1−2−glucosyltransferase
HP1413_NADPH−dependent 7−cyano−7−deazaguanine reductase
HP1412_Hypothetical protein HP1412
HP1411_Hypothetical protein HP1411
HP1409_Hypothetical protein HP1409
HP1407_Putative ribonuclease N
HP1406_Biotin synthase BioB
HP1403_Type I restriction enzyme M protein HsdM
HP1402_Type I restriction enzyme R protein HsdR
HP1401_Putative metal−dependent hydrolase
HP1400_Iron(III) dicitrate transport protein FecA3
HP1399_Arginase RocF
HP1398_Alanine dehydrogenase
HP1395_Outer membrane protein HorL
HP1393_DNA repair protein RecN
HP1392_Fibronectin/fibrinogen−binding protein
HP1388_Hypothetical protein HP1388
HP1387a_Hypothetical protein HP1387a
HP1387_DNA polymerase III subunit epsilon DnaQ
HP1386_Ribulose−phosphate 3−epimerase
HP1385_Fructose−1−6−bisphosphatase
HP1384_Hypothetical protein HP1384
HP1380_Prephenate dehydrogenase TyrA
HP1379_ATP−dependent protease La
HP1378_Competence lipoprotein ComL
HP1377_flagellar assembly protein FliW
HP1376_(3R)−hydroxymyristoyl−ACP dehydratase FabZ
HP1375_UDP−N−acetylglucosamine acyltransferase LpxA
HP1374_ATP−dependent protease ATP−binding subunit ClpX
HP1373_Rod shape−determining protein MreB
HP1372_Rod shape−determining protein MreC
HP1371_Type III restriction enzyme R protein
HP1369m_adenine−specific DNA methyltransferase
HP1368_Type IIS restriction enzyme M2 protein (mod)
HP1367_Type IIS restriction enzyme M1 protein (mod)
HP1366_Type IIS restriction enzyme R protein (MboIIR)
HP1365_OmpR family DNA−binding response regulator HP1365
HP1364_Signal−transducing protein− histidine kinase
HP1363_Bifunctional NAD(P)H−hydrate repair enzyme Nnr
HP1362_Replicative DNA helicase DnaB
HP1361_Competence locus E ComE
HP1360_4−hydroxybenzoate polyprenyltransferase UbiA
HP1359_Predicted coding region HP1359
HP1358_Hypothetical protein HP1358
HP1357_Phosphatidylserine decarboxylase
HP1356_Quinolinate synthetase NadA
HP1352_Adenine specific DNA methyltransferase
HP1351_Type II restriction endonuclease
HP1350_Carboxyl−terminal protease
HP1349_Putative periplasmic protein HP1349
HP1348_1−acyl−sn−glycerol−3−phosphate acyltransferase
HP1346_Glyceraldehyde−3−phosphate dehydrogenase
HP1345_Phosphoglycerate kinase
HP1344_Magnesium and cobalt transport protein CorA
HP1343_Integral membrane protein
HP1342_Outer membrane protein HopN
HP1339_Biopolymer transport protein ExbB
HP1338_Nickel responsive regulator NikR
HP1337_Nicotinate (nicotinamide) nucleotide adenylyltransferase NadD
HP1335_tRNA−specific 2−thiouridylase MnmA
HP1334_Hypothetical helicase HP1334
HP1333_Hypothetical protein HP1333
HP1332_Chaperone protein DnaJ
HP1331_Branched−chain amino acid transport protein AzlC
HP1330_Branched−chain amino acid transport protein AzlD
HP1329_Cation efflux system protein CzcA
HP1328_Cation efflux system protein CzcB
HP1327_Outer membrane protein HefG
HP1326_Hypothetical protein HP1326
HP1325_Fumarate hydratase FumC
HP1324_Hypothetical protein HP1324
HP1323_Ribonuclease HII
HP1322_Hypothetical protein HP1322
HP1321_Hypothetical protein HP1321
HP1320_30S ribosomal protein S10
HP1319_50S ribosomal protein L3
HP1318_50S ribosomal protein L4
HP1317_50S ribosomal protein L23
HP1316_50S ribosomal protein L2
HP1315_30S ribosomal protein S19
HP1314_50S ribosomal protein L22
HP1313_30S ribosomal protein S3
HP1312_50S ribosomal protein L16
HP1311_50S ribosomal protein L29
HP1310_30S ribosomal protein S17
HP1309_50S ribosomal protein L14
HP1305_30S ribosomal protein S8
HP1304_50S ribosomal protein L6
HP1303_50S ribosomal protein L18
HP1302_30S ribosomal protein S5
HP1301_50S ribosomal protein L15
HP1300_Preprotein translocase subunit SecY
HP1299_Methionine aminopeptidase
HP1298_Translation initiation factor IF−1
HP1297_50S ribosomal protein L36
HP1296_30S ribosomal protein S13
HP1295_30S ribosomal protein S11
HP1294_30S ribosomal protein S4
HP1293_DNA−directed RNA polymerase subunit alpha RpoA
HP1292_50S ribosomal protein L17
HP1291_Thiamine pyrophosphokinase
HP1290_Nicotinamide mononucleotide transporter PnuC
HP1289_Hypothetical protein HP1289
HP1287_Transcriptional regulator TenA
HP1286_Secreted protein HP1286
HP1285_Acid phosphatase lipoprotein
HP1284_Hypothetical protein HP1284
HP1283_Hypothetical protein HP1283
HP1282_Anthranilate synthase component I
HP1281_Anthranilate synthase component II
HP1280_Anthranilate phosphoribosyltransferase
HP1279_Bifunctional indole−3−glycerol phosphate synthase/phosphoribosylanthranilate isomerase
HP1278_Tryptophan synthase subunit beta TrpB
HP1277_Tryptophan synthase subunit alpha TrpA
HP1276_Hypothetical protein HP1276
HP1274_Paralysed flagella protein PflA
HP1273_NADH dehydrogenase subunit N
HP1272_NADH dehydrogenase subunit M
HP1271_NADH dehydrogenase subunit L
HP1270_NADH dehydrogenase subunit K
HP1267_NADH dehydrogenase subunit H
HP1266_NADH dehydrogenase subunit G
HP1265_NADH dehydrogenase I subunit F
HP1264_NADH dehydrogenase I subunit E
HP1263_NADH dehydrogenase subunit D
HP1262_NADH dehydrogenase subunit C
HP1261_NADH dehydrogenase subunit B
HP1260_NADH dehydrogenase subunit A
HP1259_NAD−dependent deacetylase
HP1258_Hypothetical protein HP1258
HP1257_Orotate phosphoribosyltransferase PyrE
HP1256_Ribosome recycling factor
HP1255_Preprotein translocase subunit SecG
HP1254_Biotin synthase BioC
HP1253_Tryptophanyl−tRNA synthetase
HP1252_Oligopeptide ABC transporter periplasmic oligopeptide−binding protein OppA
HP1251_Oligopeptide ABC transporter permease OppB
HP1250_Hypothetical protein HP1250
HP1249_Shikimate 5−dehydrogenase
HP1248_Ribonuclease R
HP1247_DNA polymerase III subunit delta
HP1246_30S ribosomal protein S6
HP1245_single−stranded DNA−binding protein
HP1243_Blood group antigen binding adhesin BabA
HP1241_Alanyl−tRNA synthetase
HP1240_Maf−like protein

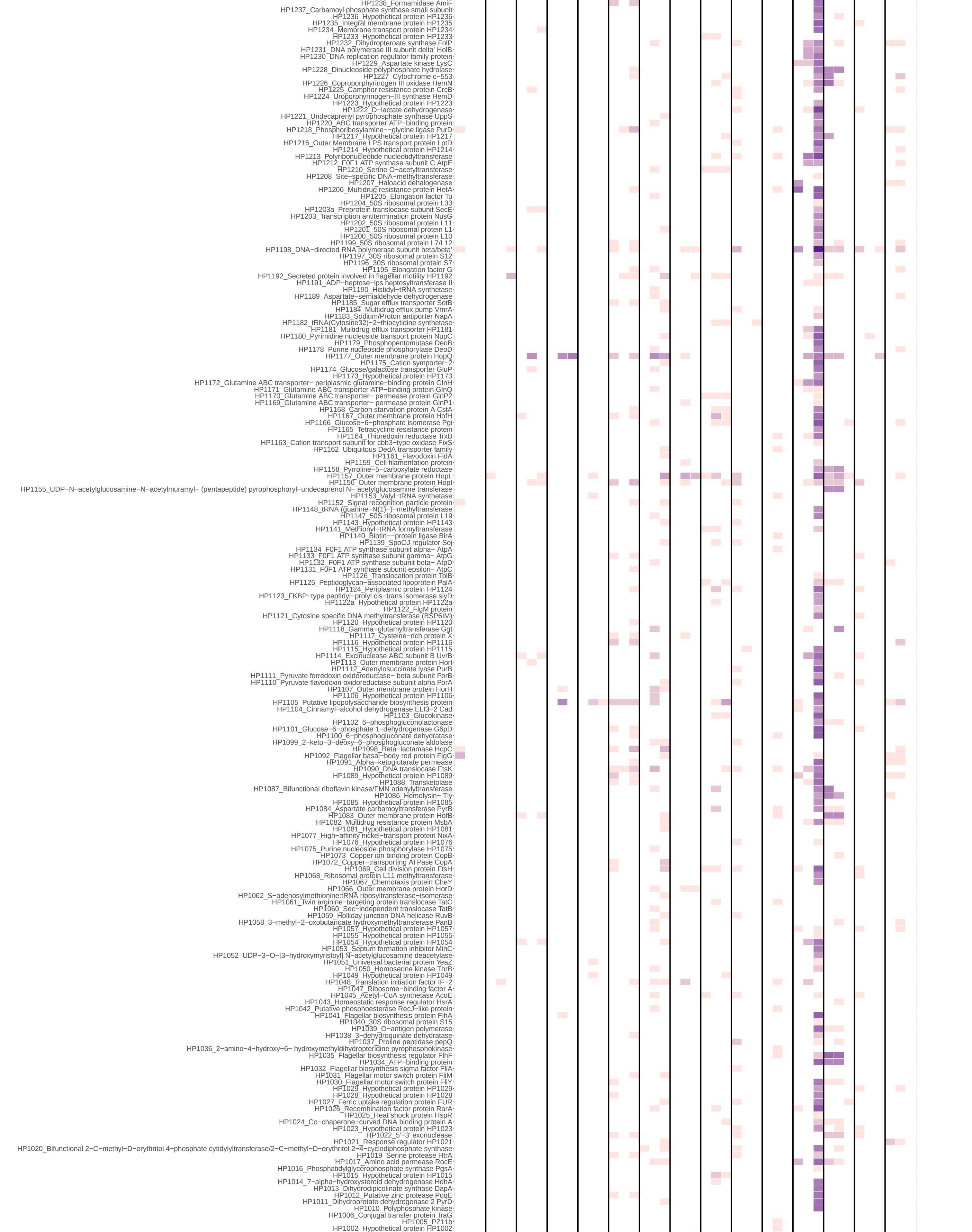
HP1238_Formamidase AmiF
HP1237_Carbamoyl phosphate synthase small subunit
HP1236_Hypothetical protein HP1236
HP1235_Integral membrane protein HP1235
HP1234_Membrane transport protein HP1234
HP1233_Hypothetical protein HP1233
HP1232_Dihydropteroate synthase FolP
HP1231_DNA polymerase III subunit delta' HolB
HP1230_DNA replication regulator family protein
HP1229_Aspartate kinase LysC
HP1228_Dinucleoside polyphosphate hydrolase
HP1227_Cytochrome c−553
HP1226_Coproporphyrinogen III oxidase HemN
HP1225_Camphor resistance protein CrcB
HP1224_Uroporphyrinogen−III synthase HemD
HP1223_Hypothetical protein HP1223
HP1222_D−lactate dehydrogenase
HP1221_Undecaprenyl pyrophosphate synthase UppS
HP1220_ABC transporter ATP−binding protein
HP1218_Phosphoribosylamine−−glycine ligase PurD
HP1217_Hypothetical protein HP1217
HP1216_Outer Membrane LPS transport protein LptD
HP1214_Hypothetical protein HP1214
HP1213_Polyribonucleotide nucleotidyltransferase
HP1212_F0F1 ATP synthase subunit C AtpE
HP1210_Serine O−acetyltransferase
HP1208_Site−specific DNA−methyltransferase
HP1207_Haloacid dehalogenase
HP1206_Multidrug resistance protein HetA
HP1205_Elongation factor Tu
HP1204_50S ribosomal protein L33
HP1203a_Preprotein translocase subunit SecE
HP1203_Transcription antitermination protein NusG
HP1202_50S ribosomal protein L11
HP1201_50S ribosomal protein L1
HP1200_50S ribosomal protein L10
HP1199_50S ribosomal protein L7/L12
HP1198_DNA−directed RNA polymerase subunit beta/beta'
HP1197_30S ribosomal protein S12
HP1196_30S ribosomal protein S7
HP1195_Elongation factor G
HP1192_Secreted protein involved in flagellar motility HP1192
HP1191_ADP−heptose−lps heptosyltransferase II
HP1190_Histidyl−tRNA synthetase
HP1189_Aspartate−semialdehyde dehydrogenase
HP1185_Sugar efflux transporter SotB
HP1184_Multidrug efflux pump VmrA
HP1183_Sodium/Proton antiporter NapA
HP1182_tRNA(Cytosine32)−2−thiocytidine synthetase
HP1181_Multidrug efflux transporter HP1181
HP1180_Pyrimidine nucleoside transport protein NupC
HP1179_Phosphopentomutase DeoB
HP1178_Purine nucleoside phosphorylase DeoD
HP1177_Outer membrane protein HopQ
HP1175_Cation symporter−2
HP1174_Glucose/galactose transporter GluP
HP1173_Hypothetical protein HP1173
HP1172_Glutamine ABC transporter− periplasmic glutamine−binding protein GlnH
HP1171_Glutamine ABC transporter ATP−binding protein GlnQ
HP1170_Glutamine ABC transporter− permease protein GlnP2
HP1169_Glutamine ABC transporter− permease protein GlnP1
HP1168_Carbon starvation protein A CstA
HP1167_Outer membrane protein HofH
HP1166_Glucose−6−phosphate isomerase Pgi
HP1165_Tetracycline resistance protein
HP1164_Thioredoxin reductase TrxB
HP1163_Cation transport subunit for cbb3−type oxidase FixS
HP1162_Ubiquitous DedA transporter family
HP1161_Flavodoxin FldA
HP1159_Cell filamentation protein
HP1158_Pyrroline−5−carboxylate reductase
HP1157_Outer membrane protein HopL
HP1156_Outer membrane protein HopI
HP1155_UDP−N−acetylglucosamine−N−acetylmuramyl− (pentapeptide) pyrophosphoryl−undecaprenol N− acetylglucosamine transferase
HP1153_Valyl−tRNA synthetase
HP1152_Signal recognition particle protein
HP1148_tRNA (guanine−N(1)−)−methyltransferase
HP1147_50S ribosomal protein L19
HP1143_Hypothetical protein HP1143
HP1141_Methionyl−tRNA formyltransferase
HP1140_Biotin−−protein ligase BirA
HP1139_SpoOJ regulator Soj
HP1134_F0F1 ATP synthase subunit alpha− AtpA
HP1133_F0F1 ATP synthase subunit gamma− AtpG
HP1132_F0F1 ATP synthase subunit beta− AtpD
HP1131_F0F1 ATP synthase subunit epsilon− AtpC
HP1126_Translocation protein TolB
HP1125_Peptidoglycan−associated lipoprotein PalA
HP1124_Periplasmic protein HP1124
HP1123_FKBP−type peptidyl−prolyl cis−trans isomerase slyD
HP1122a_Hypothetical protein HP1122a
HP1122_FlgM protein
HP1121_Cytosine specific DNA methyltransferase (BSP6IM)
HP1120_Hypothetical protein HP1120
HP1118_Gamma−glutamyltransferase Ggt
HP1117_Cysteine−rich protein X
HP1116_Hypothetical protein HP1116
HP1115_Hypothetical protein HP1115
HP1114_Excinuclease ABC subunit B UvrB
HP1113_Outer membrane protein HorI
HP1112_Adenylosuccinate lyase PurB
HP1111_Pyruvate ferredoxin oxidoreductase− beta subunit PorB
HP1110_Pyruvate flavodoxin oxidoreductase subunit alpha PorA
HP1107_Outer membrane protein HorH
HP1106_Hypothetical protein HP1106
HP1105_Putative lipopolysaccharide biosynthesis protein
HP1104_Cinnamyl−alcohol dehydrogenase ELI3−2 Cad
HP1103_Glucokinase
HP1102_6−phosphogluconolactonase
HP1101_Glucose−6−phosphate 1−dehydrogenase G6pD
HP1100_6−phosphogluconate dehydratase
HP1099_2−keto−3−deoxy−6−phosphogluconate aldolase
HP1098_Beta−lactamase HcpC
HP1092_Flagellar basal−body rod protein FlgG
HP1091_Alpha−ketoglutarate permease
HP1090_DNA translocase FtsK
HP1089_Hypothetical protein HP1089
HP1088_Transketolase
HP1087_Bifunctional riboflavin kinase/FMN adenylyltransferase
HP1086_Hemolysin− Tly
HP1085_Hypothetical protein HP1085
HP1084_Aspartate carbamoyltransferase PyrB
HP1083_Outer membrane protein HofB
HP1082_Multidrug resistance protein MsbA
HP1081_Hypothetical protein HP1081
HP1077_High−affinity nickel−transport protein NixA
HP1076_Hypothetical protein HP1076
HP1075_Purine nucleoside phosphorylase HP1075
HP1073_Copper ion binding protein CopB
HP1072_Copper−transporting ATPase CopA
HP1069_Cell division protein FtsH
HP1068_Ribosomal protein L11 methyltransferase
HP1067_Chemotaxis protein CheY
HP1066_Outer membrane protein HorD
HP1062_S−adenosylmethionine:tRNA ribosyltransferase−isomerase
HP1061_Twin arginine−targeting protein translocase TatC
HP1060_Sec−independent translocase TatB
HP1059_Holliday junction DNA helicase RuvB
HP1058_3−methyl−2−oxobutanoate hydroxymethyltransferase PanB
HP1057_Hypothetical protein HP1057
HP1055_Hypothetical protein HP1055
HP1054_Hypothetical protein HP1054
HP1053_Septum formation inhibitor MinC
HP1052_UDP−3−O−[3−hydroxymyristoyl] N−acetylglucosamine deacetylase
HP1051_Universal bacterial protein YeaZ
HP1050_Homoserine kinase ThrB
HP1049_Hypothetical protein HP1049
HP1048_Translation initiation factor IF−2
HP1047_Ribosome−binding factor A
HP1045_Acetyl−CoA synthetase AcoE
HP1043_Homeostatic response regulator HsrA
HP1042_Putative phosphoesterase RecJ−like protein
HP1041_Flagellar biosynthesis protein FlhA
HP1040_30S ribosomal protein S15
HP1039_O−antigen polymerase
HP1038_3−dehydroquinate dehydratase
HP1037_Proline peptidase pepQ
HP1036_2−amino−4−hydroxy−6− hydroxymethyldihydropteridine pyrophosphokinase
HP1035_Flagellar biosynthesis regulator FlhF
HP1034_ATP−binding protein
HP1032_Flagellar biosynthesis sigma factor FliA
HP1031_Flagellar motor switch protein FliM
HP1030_Flagellar motor switch protein FliY
HP1029_Hypothetical protein HP1029
HP1028_Hypothetical protein HP1028
HP1027_Ferric uptake regulation protein FUR
HP1026_Recombination factor protein RarA
HP1025_Heat shock protein HspR
HP1024_Co−chaperone−curved DNA binding protein A
HP1023_Hypothetical protein HP1023
HP1022_5'−3' exonuclease
HP1021_Response regulator HP1021
HP1020_Bifunctional 2−C−methyl−D−erythritol 4−phosphate cytidylyltransferase/2−C−methyl−D−erythritol 2−4−cyclodiphosphate synthase
HP1019_Serine protease HtrA
HP1017_Amino acid permease RocE
HP1016_Phosphatidylglycerophosphate synthase PgsA
HP1015_Hypothetical protein HP1015
HP1014_7−alpha−hydroxysteroid dehydrogenase HdhA
HP1013_Dihydrodipicolinate synthase DapA
HP1012_Putative zinc protease PqqE
HP1011_Dihydroorotate dehydrogenase 2 PyrD
HP1010_Polyphosphate kinase
HP1006_Conjugal transfer protein TraG
HP1005_PZ11b
HP1002_Hypothetical protein HP1002

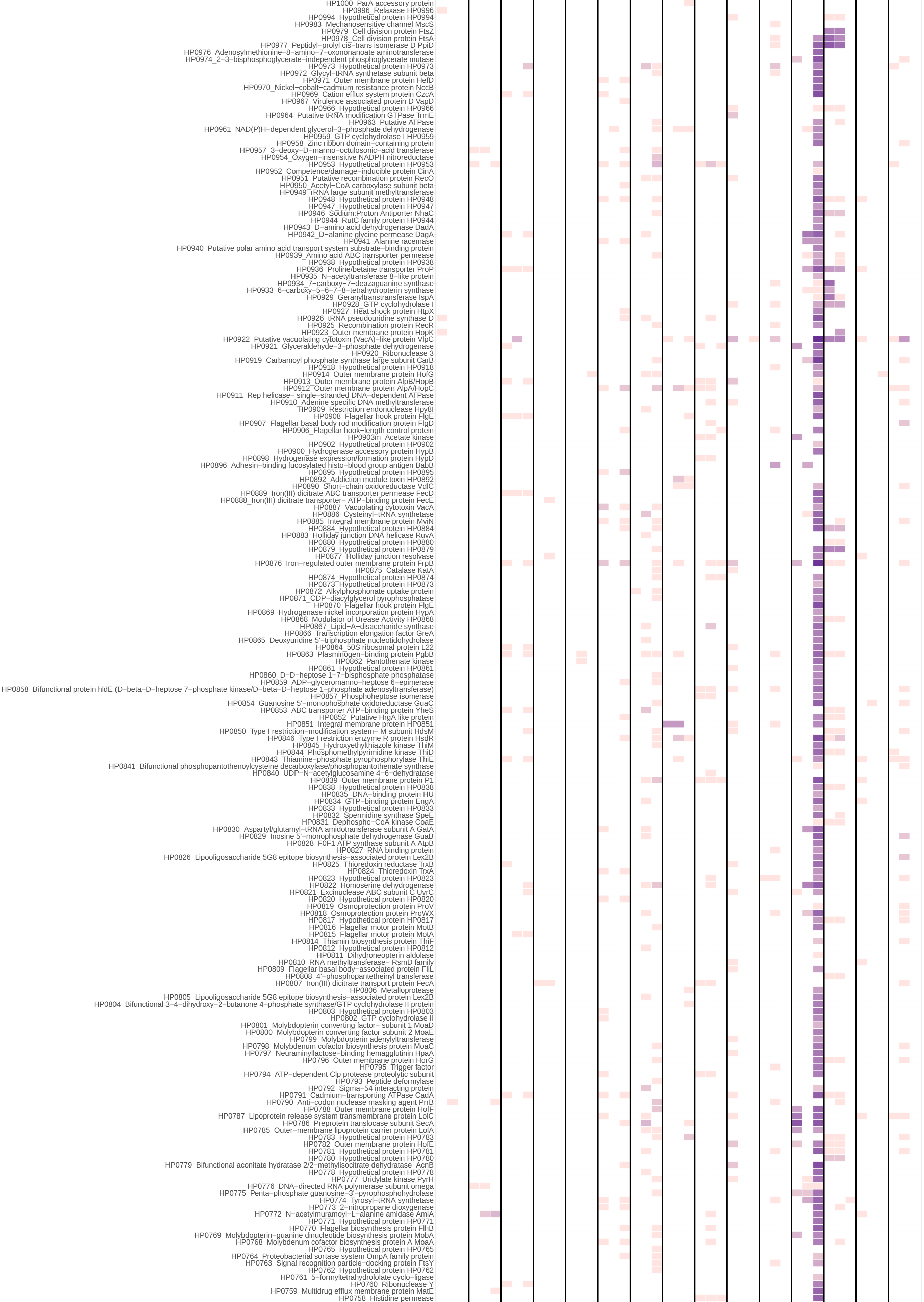

HP1000_ParA accessory protein
HP0996_Relaxase HP0996
HP0994_Hypothetical protein HP0994
HP0983_Mechanosensitive channel MscS
HP0979_Cell division protein FtsZ
HP0978_Cell division protein FtsA
HP0977_Peptidyl−prolyl cis−trans isomerase D PpiD
HP0976_Adenosylmethionine−8−amino−7−oxononanoate aminotransferase
HP0974_2−3−bisphosphoglycerate−independent phosphoglycerate mutase
HP0973_Hypothetical protein HP0973
HP0972_Glycyl−tRNA synthetase subunit beta
HP0971_Outer membrane protein HefD
HP0970_Nickel−cobalt−cadmium resistance protein NccB
HP0969_Cation efflux system protein CzcA
HP0967_Virulence associated protein D VapD
HP0966_Hypothetical protein HP0966
HP0964_Putative tRNA modification GTPase TrmE
HP0963_Putative ATPase
HP0961_NAD(P)H−dependent glycerol−3−phosphate dehydrogenase
HP0959_GTP cyclohydrolase I HP0959
HP0958_Zinc ribbon domain−containing protein
HP0957_3−deoxy−D−manno−octulosonic−acid transferase
HP0954_Oxygen−insensitive NADPH nitroreductase
HP0953_Hypothetical protein HP0953
HP0952_Competence/damage−inducible protein CinA
HP0951_Putative recombination protein RecO
HP0950_Acetyl−CoA carboxylase subunit beta
HP0949_rRNA large subunit methyltransferase
HP0948_Hypothetical protein HP0948
HP0947_Hypothetical protein HP0947
HP0946_Sodium:Proton Antiporter NhaC
HP0944_RutC family protein HP0944
HP0943_D−amino acid dehydrogenase DadA
HP0942_D−alanine glycine permease DagA
HP0941_Alanine racemase
HP0940_Putative polar amino acid transport system substrate−binding protein
HP0939_Amino acid ABC transporter permease
HP0938_Hypothetical protein HP0938
HP0936_Proline/betaine transporter ProP
HP0935_N−acetyltransferase 8−like protein
HP0934_7−carboxy−7−deazaguanine synthase
HP0933_6−carboxy−5−6−7−8−tetrahydropterin synthase
HP0929_Geranyltranstransferase IspA
HP0928_GTP cyclohydrolase I
HP0927_Heat shock protein HtpX
HP0926_tRNA pseudouridine synthase D
HP0925_Recombination protein RecR
HP0923_Outer membrane protein HopK
HP0922_Putative vacuolating cytotoxin (VacA)−like protein VlpC
HP0921_Glyceraldehyde−3−phosphate dehydrogenase
HP0920_Ribonuclease 3
HP0919_Carbamoyl phosphate synthase large subunit CarB
HP0918_Hypothetical protein HP0918
HP0914_Outer membrane protein HofG
HP0913_Outer membrane protein AlpB/HopB
HP0912_Outer membrane protein AlpA/HopC
HP0911_Rep helicase− single−stranded DNA−dependent ATPase
HP0910_Adenine specific DNA methyltransferase
HP0909_Restriction endonuclease Hpy8I
HP0908_Flagellar hook protein FlgE
HP0907_Flagellar basal body rod modification protein FlgD
HP0906_Flagellar hook−length control protein
HP0903m_Acetate kinase
HP0902_Hypothetical protein HP0902
HP0900_Hydrogenase accessory protein HypB
HP0898_Hydrogenase expression/formation protein HypD
HP0896_Adhesin−binding fucosylated histo−blood group antigen BabB
HP0895_Hypothetical protein HP0895
HP0892_Addiction module toxin HP0892
HP0890_Short−chain oxidoreductase VdlC
HP0889_Iron(III) dicitrate ABC transporter permease FecD
HP0888_Iron(III) dicitrate transporter− ATP−binding protein FecE
HP0887_Vacuolating cytotoxin VacA
HP0886_Cysteinyl−tRNA synthetase
HP0885_Integral membrane protein MviN
HP0884_Hypothetical protein HP0884
HP0883_Holliday junction DNA helicase RuvA
HP0880_Hypothetical protein HP0880
HP0879_Hypothetical protein HP0879
HP0877_Holliday junction resolvase
HP0876_Iron−regulated outer membrane protein FrpB
HP0875_Catalase KatA
HP0874_Hypothetical protein HP0874
HP0873_Hypothetical protein HP0873
HP0872_Alkylphosphonate uptake protein
HP0871_CDP−diacylglycerol pyrophosphatase
HP0870_Flagellar hook protein FlgE
HP0869_Hydrogenase nickel incorporation protein HypA
HP0868_Modulator of Urease Activity HP0868
HP0867_Lipid−A−disaccharide synthase
HP0866_Transcription elongation factor GreA
HP0865_Deoxyuridine 5'−triphosphate nucleotidohydrolase
HP0864_50S ribosomal protein L22
HP0863_Plasminogen−binding protein PgbB
HP0862_Pantothenate kinase
HP0861_Hypothetical protein HP0861
HP0860_D−D−heptose 1−7−bisphosphate phosphatase
HP0859_ADP−glyceromanno−heptose 6−epimerase
HP0858_Bifunctional protein hldE (D−beta−D−heptose 7−phosphate kinase/D−beta−D−heptose 1−phosphate adenosyltransferase)
HP0857_Phosphoheptose isomerase
HP0854_Guanosine 5'−monophosphate oxidoreductase GuaC
HP0853_ABC transporter ATP−binding protein YheS
HP0852_Putative HrgA like protein
HP0851_Integral membrane protein HP0851
HP0850_Type I restriction−modification system− M subunit HdsM
HP0846_Type I restriction enzyme R protein HsdR
HP0845_Hydroxyethylthiazole kinase ThiM
HP0844_Phosphomethylpyrimidine kinase ThiD
HP0843_Thiamine−phosphate pyrophosphorylase ThiE
HP0841_Bifunctional phosphopantothenoylcysteine decarboxylase/phosphopantothenate synthase
HP0840_UDP−N−acetylglucosamine 4−6−dehydratase
HP0839_Outer membrane protein P1
HP0838_Hypothetical protein HP0838
HP0835_DNA−binding protein HU
HP0834_GTP−binding protein EngA
HP0833_Hypothetical protein HP0833
HP0832_Spermidine synthase SpeE
HP0831_Dephospho−CoA kinase CoaE
HP0830_Aspartyl/glutamyl−tRNA amidotransferase subunit A GatA
HP0829_Inosine 5'−monophosphate dehydrogenase GuaB
HP0828_F0F1 ATP synthase subunit A AtpB
HP0827_RNA binding protein
HP0826_Lipooligosaccharide 5G8 epitope biosynthesis−associated protein Lex2B
HP0825_Thioredoxin reductase TrxB
HP0824_Thioredoxin TrxA
HP0823_Hypothetical protein HP0823
HP0822_Homoserine dehydrogenase
HP0821_Excinuclease ABC subunit C UvrC
HP0820_Hypothetical protein HP0820
HP0819_Osmoprotection protein ProV
HP0818_Osmoprotection protein ProWX
HP0817_Hypothetical protein HP0817
HP0816_Flagellar motor protein MotB
HP0815_Flagellar motor protein MotA
HP0814_Thiamin biosynthesis protein ThiF
HP0812_Hypothetical protein HP0812
HP0811_Dihydroneopterin aldolase
HP0810_RNA methyltransferase− RsmD family
HP0809_Flagellar basal body−associated protein FliL
HP0808_4'−phosphopantetheinyl transferase
HP0807_Iron(III) dicitrate transport protein FecA
HP0806_Metalloprotease
HP0805_Lipooligosaccharide 5G8 epitope biosynthesis−associated protein Lex2B
HP0804_Bifunctional 3−4−dihydroxy−2−butanone 4−phosphate synthase/GTP cyclohydrolase II protein
HP0803_Hypothetical protein HP0803
HP0802_GTP cyclohydrolase II
HP0801_Molybdopterin converting factor− subunit 1 MoaD
HP0800_Molybdopterin converting factor subunit 2 MoaE
HP0799_Molybdopterin adenylyltransferase
HP0798_Molybdenum cofactor biosynthesis protein MoaC
HP0797_Neuraminyllactose−binding hemagglutinin HpaA
HP0796_Outer membrane protein HorG
HP0795_Trigger factor
HP0794_ATP−dependent Clp protease proteolytic subunit
HP0793_Peptide deformylase
HP0792_Sigma−54 interacting protein
HP0791_Cadmium−transporting ATPase CadA
HP0790_Anti−codon nuclease masking agent PrrB
HP0788_Outer membrane protein HofF
HP0787_Lipoprotein release system transmembrane protein LolC
HP0786_Preprotein translocase subunit SecA
HP0785_Outer−membrane lipoprotein carrier protein LolA
HP0783_Hypothetical protein HP0783
HP0782_Outer membrane protein HofE
HP0781_Hypothetical protein HP0781
HP0780_Hypothetical protein HP0780
HP0779_Bifunctional aconitate hydratase 2/2−methylisocitrate dehydratase AcnB
HP0778_Hypothetical protein HP0778
HP0777_Uridylate kinase PyrH
HP0776_DNA−directed RNA polymerase subunit omega
HP0775_Penta−phosphate guanosine−3'−pyrophosphohydrolase
HP0774_Tyrosyl−tRNA synthetase
HP0773_2−nitropropane dioxygenase
HP0772_N−acetylmuramoyl−L−alanine amidase AmiA
HP0771_Hypothetical protein HP0771
HP0770_Flagellar biosynthesis protein FlhB
HP0769_Molybdopterin−guanine dinucleotide biosynthesis protein MobA
HP0768_Molybdenum cofactor biosynthesis protein A MoaA
HP0765_Hypothetical protein HP0765
HP0764_Proteobacterial sortase system OmpA family protein
HP0763_Signal recognition particle−docking protein FtsY
HP0762_Hypothetical protein HP0762
HP0761_5−formyltetrahydrofolate cyclo−ligase
HP0760_Ribonuclease Y
HP0759_Multidrug efflux membrane protein MatE
HP0758_Histidine permease

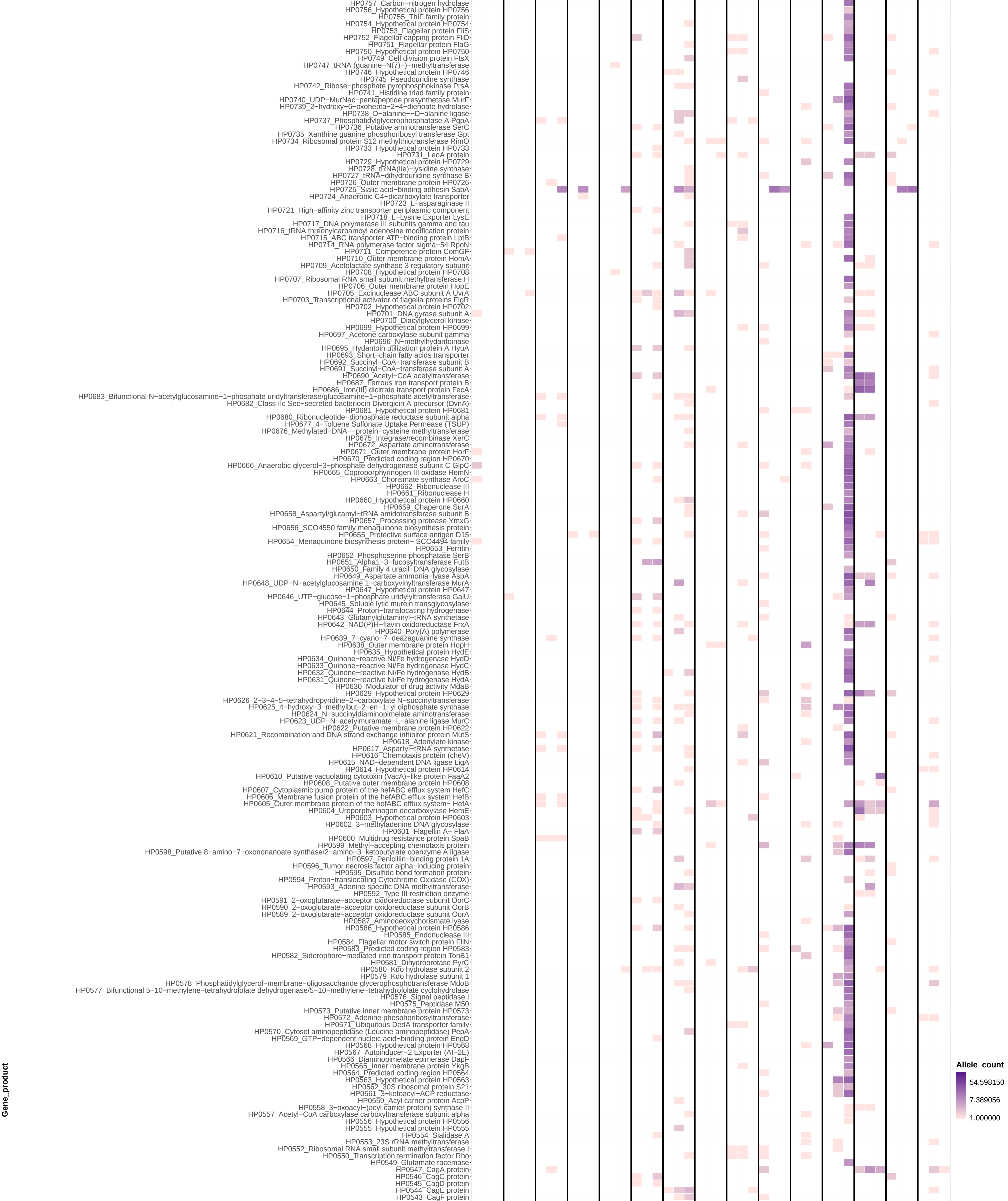
HP0757_Carbon−nitrogen hydrolase
HP0756_Hypothetical protein HP0756
HP0755_ThiF family protein
HP0754_Hypothetical protein HP0754
HP0753_Flagellar protein FliS
HP0752_Flagellar capping protein FliD
HP0751_Flagellar protein FlaG
HP0750_Hypothetical protein HP0750
HP0749_Cell division protein FtsX
HP0747_tRNA (guanine−N(7)−)−methyltransferase
HP0746_Hypothetical protein HP0746
HP0745_Pseudouridine synthase
HP0742_Ribose−phosphate pyrophosphokinase PrsA
HP0741_Histidine triad family protein
HP0740_UDP−MurNac−pentapeptide presynthetase MurF
HP0739_2−hydroxy−6−oxohepta−2−4−dienoate hydrolase
HP0738_D−alanine−−D−alanine ligase
HP0737_Phosphatidylglycerophosphatase A PgpA
HP0736_Putative aminotransferase SerC
HP0735_Xanthine guanine phosphoribosyl transferase Gpt
HP0734_Ribosomal protein S12 methylthiotransferase RimO
HP0733_Hypothetical protein HP0733
HP0731_LeoA protein
HP0729_Hypothetical protein HP0729
HP0728_tRNA(Ile)−lysidine synthase
HP0727_tRNA−dihydrouridine synthase B
HP0726_Outer membrane protein HP0726
HP0725_Sialic acid−binding adhesin SabA
HP0724_Anaerobic C4−dicarboxylate transporter
HP0723_L−asparaginase II
HP0721_High−affinity zinc transporter periplasmic component
HP0718_L−Lysine Exporter LysE
HP0717_DNA polymerase III subunits gamma and tau
HP0716_tRNA threonylcarbamoyl adenosine modification protein
HP0715_ABC transporter ATP−binding protein LptB
HP0714_RNA polymerase factor sigma−54 RpoN
HP0711_Competence protein ComGF
HP0710_Outer membrane protein HomA
HP0709_Acetolactate synthase 3 regulatory subunit
HP0708_Hypothetical protein HP0708
HP0707_Ribosomal RNA small subunit methyltransferase H
HP0706_Outer membrane protein HopE
HP0705_Excinuclease ABC subunit A UvrA
HP0703_Transcriptional activator of flagella proteins FlgR
HP0702_Hypothetical protein HP0702
HP0701_DNA gyrase subunit A
HP0700_Diacylglycerol kinase
HP0699_Hypothetical protein HP0699
HP0697_Acetone carboxylase subunit gamma
HP0696_N−methylhydantoinase
HP0695_Hydantoin utilization protein A HyuA
HP0693_Short−chain fatty acids transporter
HP0692_Succinyl−CoA−transferase subunit B
HP0691_Succinyl−CoA−transferase subunit A
HP0690_Acetyl−CoA acetyltransferase
HP0687_Ferrous iron transport protein B
HP0686_Iron(III) dicitrate transport protein FecA
HP0683_Bifunctional N−acetylglucosamine−1−phosphate uridyltransferase/glucosamine−1−phosphate acetyltransferase
HP0682_Class IIc Sec−secreted bacteriocin Divergicin A precursor (DvnA)
HP0681_Hypothetical protein HP0681
HP0680_Ribonucleotide−diphosphate reductase subunit alpha
HP0677_4−Toluene Sulfonate Uptake Permease (TSUP)
HP0676_Methylated−DNA−−protein−cysteine methyltransferase
HP0675_Integrase/recombinase XerC
HP0672_Aspartate aminotransferase
HP0671_Outer membrane protein HorF
HP0670_Predicted coding region HP0670
HP0666_Anaerobic glycerol−3−phosphate dehydrogenase subunit C GlpC
HP0665_Coproporphyrinogen III oxidase HemN
HP0663_Chorismate synthase AroC
HP0662_Ribonuclease III
HP0661_Ribonuclease H
HP0660_Hypothetical protein HP0660
HP0659_Chaperone SurA
HP0658_Aspartyl/glutamyl−tRNA amidotransferase subunit B
HP0657_Processing protease YmxG
HP0656_SCO4550 family menaquinone biosynthesis protein
HP0655_Protective surface antigen D15
HP0654_Menaquinone biosynthesis protein− SCO4494 family
HP0653_Ferritin
HP0652_Phosphoserine phosphatase SerB
HP0651_Alpha1−3−fucosyltransferase FutB
HP0650_Family 4 uracil−DNA glycosylase
HP0649_Aspartate ammonia−lyase AspA
HP0648_UDP−N−acetylglucosamine 1−carboxyvinyltransferase MurA
HP0647_Hypothetical protein HP0647
HP0646_UTP−glucose−1−phosphate uridylyltransferase GalU
HP0645_Soluble lytic murein transglycosylase
HP0644_Proton−translocating hydrogenase
HP0643_Glutamylglutaminyl−tRNA synthetase
HP0642_NAD(P)H−flavin oxidoreductase FrxA
HP0640_Poly(A) polymerase
HP0639_7−cyano−7−deazaguanine synthase
HP0638_Outer membrane protein HopH
HP0635_Hypothetical protein HydE
HP0634_Quinone−reactive Ni/Fe hydrogenase HydD
HP0633_Quinone−reactive Ni/Fe hydrogenase HydC
HP0632_Quinone−reactive Ni/Fe hydrogenase HydB
HP0631_Quinone−reactive Ni/Fe hydrogenase HydA
HP0630_Modulator of drug activity MdaB
HP0629_Hypothetical protein HP0629
HP0626_2−3−4−5−tetrahydropyridine−2−carboxylate N−succinyltransferase
HP0625_4−hydroxy−3−methylbut−2−en−1−yl diphosphate synthase
HP0624_N−succinyldiaminopimelate aminotransferase
HP0623_UDP−N−acetylmuramate−L−alanine ligase MurC
HP0622_Putative membrane protein HP0622
HP0621_Recombination and DNA strand exchange inhibitor protein MutS
HP0618_Adenylate kinase
HP0617_Aspartyl−tRNA synthetase
HP0616_Chemotaxis protein (cheV)
HP0615_NAD−dependent DNA ligase LigA
HP0614_Hypothetical protein HP0614
HP0610_Putative vacuolating cytotoxin (VacA)−like protein FaaA2
HP0608_Putative outer membrane protein HP0608
HP0607_Cytoplasmic pump protein of the hefABC efflux system HefC
HP0606_Membrane fusion protein of the hefABC efflux system HefB
HP0605_Outer membrane protein of the hefABC efflux system− HefA
HP0604_Uroporphyrinogen decarboxylase HemE
HP0603_Hypothetical protein HP0603
HP0602_3−methyladenine DNA glycosylase
HP0601_Flagellin A− FlaA
HP0600_Multidrug resistance protein SpaB
HP0599_Methyl−accepting chemotaxis protein
HP0598_Putative 8−amino−7−oxononanoate synthase/2−amino−3−ketobutyrate coenzyme A ligase
HP0597_Penicillin−binding protein 1A
HP0596_Tumor necrosis factor alpha−inducing protein
HP0595_Disulfide bond formation protein
HP0594_Proton−translocating Cytochrome Oxidase (COX)
HP0593_Adenine specific DNA methyltransferase
HP0592_Type III restriction enzyme
HP0591_2−oxoglutarate−acceptor oxidoreductase subunit OorC
HP0590_2−oxoglutarate−acceptor oxidoreductase subunit OorB
HP0589_2−oxoglutarate−acceptor oxidoreductase subunit OorA
HP0587_Aminodeoxychorismate lyase
HP0586_Hypothetical protein HP0586
HP0585_Endonuclease III
HP0584_Flagellar motor switch protein FliN
HP0583_Predicted coding region HP0583
HP0582_Siderophore−mediated iron transport protein TonB1
HP0581_Dihydroorotase PyrC
HP0580_Kdo hydrolase subunit 2
HP0579_Kdo hydrolase subunit 1
HP0578_Phosphatidylglycerol−membrane−oligosaccharide glycerophosphotransferase MdoB
HP0577_Bifunctional 5−10−methylene−tetrahydrofolate dehydrogenase/5−10−methylene−tetrahydrofolate cyclohydrolase
HP0576_Signal peptidase I
HP0575_Peptidase M50
HP0573_Putative inner membrane protein HP0573
HP0572_Adenine phosphoribosyltransferase
HP0571_Ubiquitous DedA transporter family
HP0570_Cytosol aminopeptidase (Leucine aminopeptidase) PepA
HP0569_GTP−dependent nucleic acid−binding protein EngD
HP0568_Hypothetical protein HP0568
HP0567_Autoinducer−2 Exporter (AI−2E)
HP0566_Diaminopimelate epimerase DapF
HP0565_Inner membrane protein YkgB
HP0564_Predicted coding region HP0564
HP0563_Hypothetical protein HP0563
HP0562_30S ribosomal protein S21
HP0561_3−ketoacyl−ACP reductase
HP0559_Acyl carrier protein AcpP
HP0558_3−oxoacyl−(acyl carrier protein) synthase II
HP0557_Acetyl−CoA carboxylase carboxyltransferase subunit alpha
HP0556_Hypothetical protein HP0556
HP0555_Hypothetical protein HP0555
HP0554_Sialidase A
HP0553_23S rRNA methyltransferase
HP0552_Ribosomal RNA small subunit methyltransferase I
HP0550_Transcription termination factor Rho
HP0549_Glutamate racemase
HP0547_CagA protein
HP0546_CagC protein
HP0545_CagD protein
HP0544_CagE protein
HP0543_CagF protein
Gene_product
Allele_count
54.598150
7.389056
1.000000

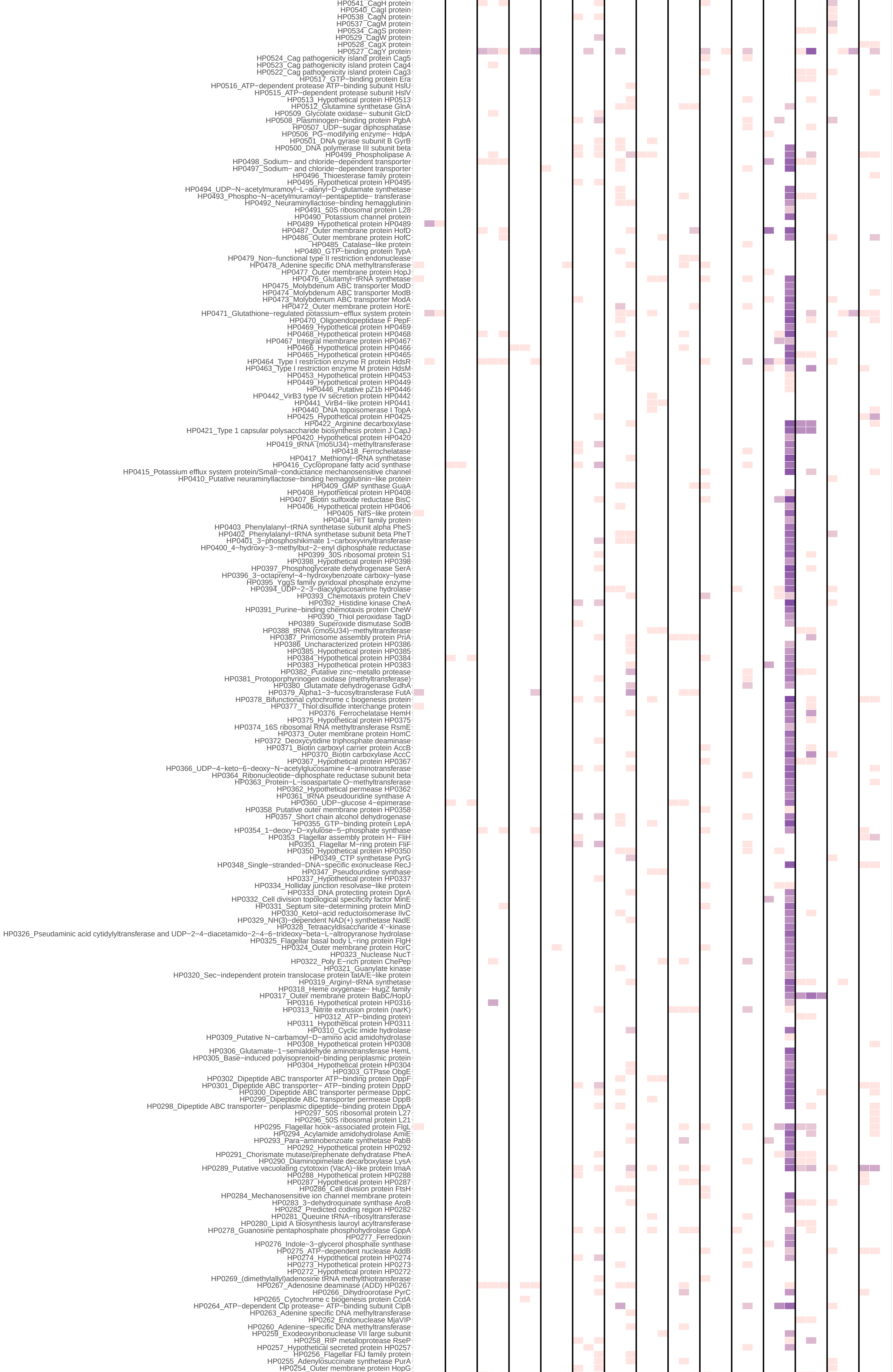
HP0541_CagH protein
HP0540_CagI protein
HP0538_CagN protein
HP0537_CagM protein
HP0534_CagS protein
HP0529_CagW protein
HP0528_CagX protein
HP0527_CagY protein
HP0524_Cag pathogenicity island protein Cag5
HP0523_Cag pathogenicity island protein Cag4
HP0522_Cag pathogenicity island protein Cag3
HP0517_GTP−binding protein Era
HP0516_ATP−dependent protease ATP−binding subunit HslU
HP0515_ATP−dependent protease subunit HslV
HP0513_Hypothetical protein HP0513
HP0512_Glutamine synthetase GlnA
HP0509_Glycolate oxidase− subunit GlcD
HP0508_Plasminogen−binding protein PgbA
HP0507_UDP−sugar diphosphatase
HP0506_PG−modifying enzyme− HdpA
HP0501_DNA gyrase subunit B GyrB
HP0500_DNA polymerase III subunit beta
HP0499_Phospholipase A
HP0498_Sodium− and chloride−dependent transporter
HP0497_Sodium− and chloride−dependent transporter
HP0496_Thioesterase family protein
HP0495_Hypothetical protein HP0495
HP0494_UDP−N−acetylmuramoyl−L−alanyl−D−glutamate synthetase
HP0493_Phospho−N−acetylmuramoyl−pentapeptide− transferase
HP0492_Neuraminyllactose−binding hemagglutinin
HP0491_50S ribosomal protein L28
HP0490_Potassium channel protein
HP0489_Hypothetical protein HP0489
HP0487_Outer membrane protein HofD
HP0486_Outer membrane protein HofC
HP0485_Catalase−like protein
HP0480_GTP−binding protein TypA
HP0479_Non−functional type II restriction endonuclease
HP0478_Adenine specific DNA methyltransferase
HP0477_Outer membrane protein HopJ
HP0476_Glutamyl−tRNA synthetase
HP0475_Molybdenum ABC transporter ModD
HP0474_Molybdenum ABC transporter ModB
HP0473_Molybdenum ABC transporter ModA
HP0472_Outer membrane protein HorE
HP0471_Glutathione−regulated potassium−efflux system protein
HP0470_Oligoendopeptidase F PepF
HP0469_Hypothetical protein HP0469
HP0468_Hypothetical protein HP0468
HP0467_Integral membrane protein HP0467
HP0466_Hypothetical protein HP0466
HP0465_Hypothetical protein HP0465
HP0464_Type I restriction enzyme R protein HsdR
HP0463_Type I restriction enzyme M protein HsdM
HP0453_Hypothetical protein HP0453
HP0449_Hypothetical protein HP0449
HP0446_Putative pZ1b HP0446
HP0442_VirB3 type IV secretion protein HP0442
HP0441_VirB4−like protein HP0441
HP0440_DNA topoisomerase I TopA
HP0425_Hypothetical protein HP0425
HP0422_Arginine decarboxylase
HP0421_Type 1 capsular polysaccharide biosynthesis protein J CapJ
HP0420_Hypothetical protein HP0420
HP0419_tRNA (mo5U34)−methyltransferase
HP0418_Ferrochelatase
HP0417_Methionyl−tRNA synthetase
HP0416_Cyclopropane fatty acid synthase
HP0415_Potassium efflux system protein/Small−conductance mechanosensitive channel
HP0410_Putative neuraminyllactose−binding hemagglutinin−like protein
HP0409_GMP synthase GuaA
HP0408_Hypothetical protein HP0408
HP0407_Biotin sulfoxide reductase BisC
HP0406_Hypothetical protein HP0406
HP0405_NifS−like protein
HP0404_HIT family protein
HP0403_Phenylalanyl−tRNA synthetase subunit alpha PheS
HP0402_Phenylalanyl−tRNA synthetase subunit beta PheT
HP0401_3−phosphoshikimate 1−carboxyvinyltransferase
HP0400_4−hydroxy−3−methylbut−2−enyl diphosphate reductase
HP0399_30S ribosomal protein S1
HP0398_Hypothetical protein HP0398
HP0397_Phosphoglycerate dehydrogenase SerA
HP0396_3−octaprenyl−4−hydroxybenzoate carboxy−lyase
HP0395_YggS family pyridoxal phosphate enzyme
HP0394_UDP−2−3−diacylglucosamine hydrolase
HP0393_Chemotaxis protein CheV
HP0392_Histidine kinase CheA
HP0391_Purine−binding chemotaxis protein CheW
HP0390_Thiol peroxidase TagD
HP0389_Superoxide dismutase SodB
HP0388_tRNA (cmo5U34)−methyltransferase
HP0387_Primosome assembly protein PriA
HP0386_Uncharacterized protein HP0386
HP0385_Hypothetical protein HP0385
HP0384_Hypothetical protein HP0384
HP0383_Hypothetical protein HP0383
HP0382_Putative zinc−metallo protease
HP0381_Protoporphyrinogen oxidase (methyltransferase)
HP0380_Glutamate dehydrogenase GdhA
HP0379_Alpha1−3−fucosyltransferase FutA
HP0378_Bifunctional cytochrome c biogenesis protein
HP0377_Thiol:disulfide interchange protein
HP0376_Ferrochelatase HemH
HP0375_Hypothetical protein HP0375
HP0374_16S ribosomal RNA methyltransferase RsmE
HP0373_Outer membrane protein HomC
HP0372_Deoxycytidine triphosphate deaminase
HP0371_Biotin carboxyl carrier protein AccB
HP0370_Biotin carboxylase AccC
HP0367_Hypothetical protein HP0367
HP0366_UDP−4−keto−6−deoxy−N−acetylglucosamine 4−aminotransferase
HP0364_Ribonucleotide−diphosphate reductase subunit beta
HP0363_Protein−L−isoaspartate O−methyltransferase
HP0362_Hypothetical permease HP0362
HP0361_tRNA pseudouridine synthase A
HP0360_UDP−glucose 4−epimerase
HP0358_Putative outer membrane protein HP0358
HP0357_Short chain alcohol dehydrogenase
HP0355_GTP−binding protein LepA
HP0354_1−deoxy−D−xylulose−5−phosphate synthase
HP0353_Flagellar assembly protein H− FliH
HP0351_Flagellar M−ring protein FliF
HP0350_Hypothetical protein HP0350
HP0349_CTP synthetase PyrG
HP0348_Single−stranded−DNA−specific exonuclease RecJ
HP0347_Pseudouridine synthase
HP0337_Hypothetical protein HP0337
HP0334_Holliday junction resolvase−like protein
HP0333_DNA protecting protein DprA
HP0332_Cell division topological specificity factor MinE
HP0331_Septum site−determining protein MinD
HP0330_Ketol−acid reductoisomerase IlvC
HP0329_NH(3)−dependent NAD(+) synthetase NadE
HP0328_Tetraacyldisaccharide 4'−kinase
HP0326_Pseudaminic acid cytidylyltransferase and UDP−2−4−diacetamido−2−4−6−trideoxy−beta−L−altropyranose hydrolase
HP0325_Flagellar basal body L−ring protein FlgH
HP0324_Outer membrane protein HorC
HP0323_Nuclease NucT
HP0322_Poly E−rich protein ChePep
HP0321_Guanylate kinase
HP0320_Sec−independent protein translocase protein TatA/E−like protein
HP0319_Arginyl−tRNA synthetase
HP0318_Heme oxygenase− HugZ family
HP0317_Outer membrane protein BabC/HopU
HP0316_Hypothetical protein HP0316
HP0313_Nitrite extrusion protein (narK)
HP0312_ATP−binding protein
HP0311_Hypothetical protein HP0311
HP0310_Cyclic imide hydrolase
HP0309_Putative N−carbamoyl−D−amino acid amidohydrolase
HP0308_Hypothetical protein HP0308
HP0306_Glutamate−1−semialdehyde aminotransferase HemL
HP0305_Base−induced polyisoprenoid−binding periplasmic protein
HP0304_Hypothetical protein HP0304
HP0303_GTPase ObgE
HP0302_Dipeptide ABC transporter ATP−binding protein DppF
HP0301_Dipeptide ABC transporter− ATP−binding protein DppD
HP0300_Dipeptide ABC transporter permease DppC
HP0299_Dipeptide ABC transporter permease DppB
HP0298_Dipeptide ABC transporter− periplasmic dipeptide−binding protein DppA
HP0297_50S ribosomal protein L27
HP0296_50S ribosomal protein L21
HP0295_Flagellar hook−associated protein FlgL
HP0294_Acylamide amidohydrolase AmiE
HP0293_Para−aminobenzoate synthetase PabB
HP0292_Hypothetical protein HP0292
HP0291_Chorismate mutase/prephenate dehydratase PheA
HP0290_Diaminopimelate decarboxylase LysA
HP0289_Putative vacuolating cytotoxin (VacA)−like protein ImaA
HP0288_Hypothetical protein HP0288
HP0287_Hypothetical protein HP0287
HP0286_Cell division protein FtsH
HP0284_Mechanosensitive ion channel membrane protein
HP0283_3−dehydroquinate synthase AroB
HP0282_Predicted coding region HP0282
HP0281_Queuine tRNA−ribosyltransferase
HP0280_Lipid A biosynthesis lauroyl acyltransferase
HP0278_Guanosine pentaphosphate phosphohydrolase GppA
HP0277_Ferredoxin
HP0276_Indole−3−glycerol phosphate synthase
HP0275_ATP−dependent nuclease AddB
HP0274_Hypothetical protein HP0274
HP0273_Hypothetical protein HP0273
HP0272_Hypothetical protein HP0272
HP0269_(dimethylallyl)adenosine tRNA methylthiotransferase
HP0267_Adenosine deaminase (ADD) HP0267
HP0266_Dihydroorotase PyrC
HP0265_Cytochrome c biogenesis protein CcdA
HP0264_ATP−dependent Clp protease− ATP−binding subunit ClpB
HP0263_Adenine specific DNA methyltransferase
HP0262_Endonuclease MjaVIP
HP0260_Adenine−specific DNA methyltransferase
HP0259_Exodeoxyribonuclease VII large subunit
HP0258_RIP metalloprotease RseP
HP0257_Hypothetical secreted protein HP0257
HP0256_Flagellar FliJ family protein
HP0255_Adenylosuccinate synthetase PurA
HP0254_Outer membrane protein HopG

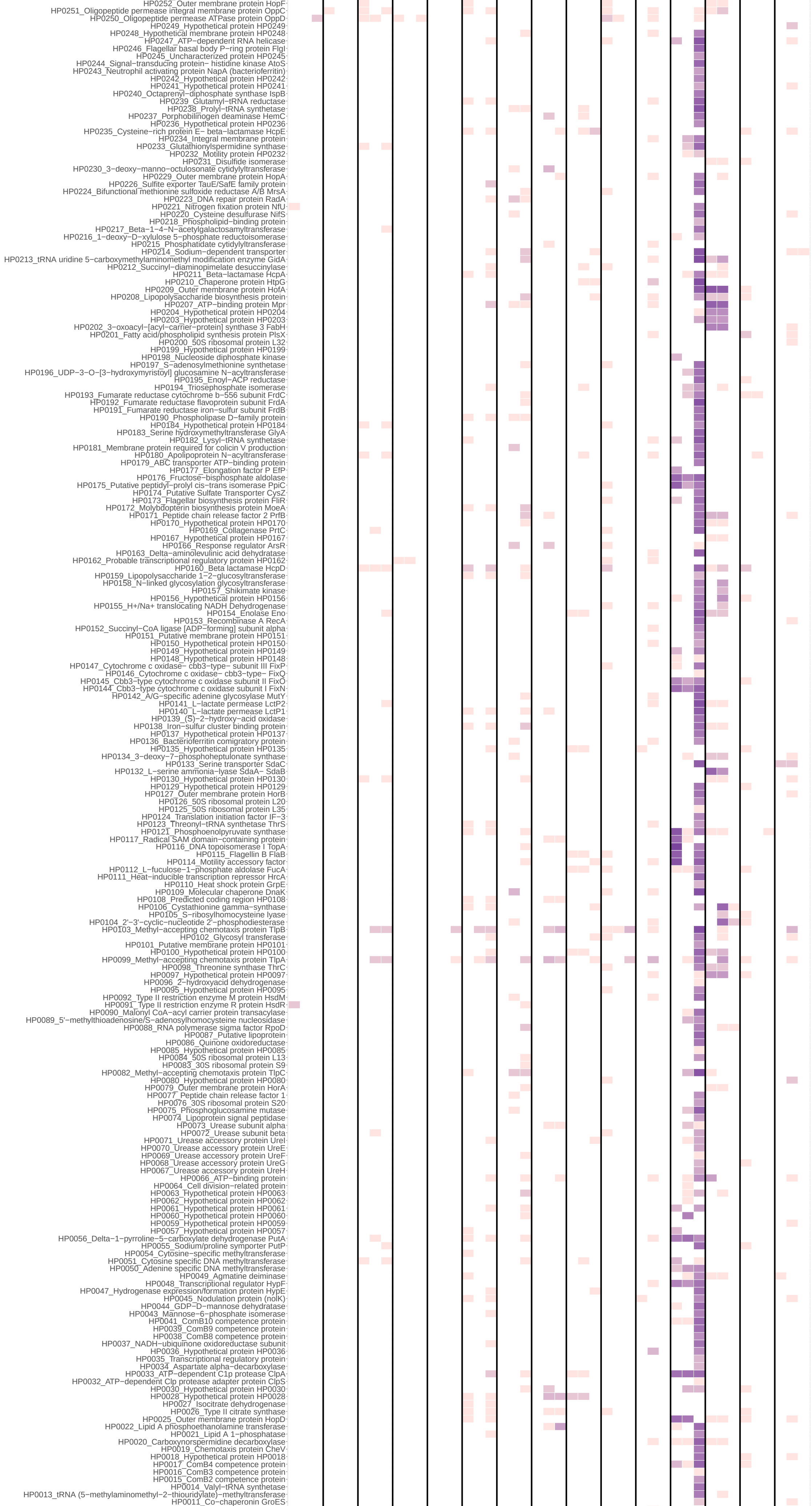

HP0252_Outer membrane protein HopF
HP0251_Oligopeptide permease integral membrane protein OppC
HP0250_Oligopeptide permease ATPase protein OppD
HP0249_Hypothetical protein HP0249
HP0248_Hypothetical membrane protein HP0248
HP0247_ATP−dependent RNA helicase
HP0246_Flagellar basal body P−ring protein FlgI
HP0245_Uncharacterized protein HP0245
HP0244_Signal−transducing protein− histidine kinase AtoS
HP0243_Neutrophil activating protein NapA (bacterioferritin)
HP0242_Hypothetical protein HP0242
HP0241_Hypothetical protein HP0241
HP0240_Octaprenyl−diphosphate synthase IspB
HP0239_Glutamyl−tRNA reductase
HP0238_Prolyl−tRNA synthetase
HP0237_Porphobilinogen deaminase HemC
HP0236_Hypothetical protein HP0236
HP0235_Cysteine−rich protein E− beta−lactamase HcpE
HP0234_Integral membrane protein
HP0233_Glutathionylspermidine synthase
HP0232_Motility protein HP0232
HP0231_Disulfide isomerase
HP0230_3−deoxy−manno−octulosonate cytidylyltransferase
HP0229_Outer membrane protein HopA
HP0226_Sulfite exporter TauE/SafE family protein
HP0224_Bifunctional methionine sulfoxide reductase A/B MrsA
HP0223_DNA repair protein RadA
HP0221_Nitrogen fixation protein NfU
HP0220_Cysteine desulfurase NifS
HP0218_Phospholipid−binding protein
HP0217_Beta−1−4−N−acetylgalactosamyltransferase
HP0216_1−deoxy−D−xylulose 5−phosphate reductoisomerase
HP0215_Phosphatidate cytidylyltransferase
HP0214_Sodium−dependent transporter
HP0213_tRNA uridine 5−carboxymethylaminomethyl modification enzyme GidA
HP0212_Succinyl−diaminopimelate desuccinylase
HP0211_Beta−lactamase HcpA
HP0210_Chaperone protein HtpG
HP0209_Outer membrane protein HofA
HP0208_Lipopolysaccharide biosynthesis protein
HP0207_ATP−binding protein Mpr
HP0204_Hypothetical protein HP0204
HP0203_Hypothetical protein HP0203
HP0202_3−oxoacyl−[acyl−carrier−protein] synthase 3 FabH
HP0201_Fatty acid/phospholipid synthesis protein PlsX
HP0200_50S ribosomal protein L32
HP0199_Hypothetical protein HP0199
HP0198_Nucleoside diphosphate kinase
HP0197_S−adenosylmethionine synthetase
HP0196_UDP−3−O−[3−hydroxymyristoyl] glucosamine N−acyltransferase
HP0195_Enoyl−ACP reductase
HP0194_Triosephosphate isomerase
HP0193_Fumarate reductase cytochrome b−556 subunit FrdC
HP0192_Fumarate reductase flavoprotein subunit FrdA
HP0191_Fumarate reductase iron−sulfur subunit FrdB
HP0190_Phospholipase D−family protein
HP0184_Hypothetical protein HP0184
HP0183_Serine hydroxymethyltransferase GlyA
HP0182_Lysyl−tRNA synthetase
HP0181_Membrane protein required for colicin V production
HP0180_Apolipoprotein N−acyltransferase
HP0179_ABC transporter ATP−binding protein
HP0177_Elongation factor P EfP
HP0176_Fructose−bisphosphate aldolase
HP0175_Putative peptidyl−prolyl cis−trans isomerase PpiC
HP0174_Putative Sulfate Transporter CysZ
HP0173_Flagellar biosynthesis protein FliR
HP0172_Molybdopterin biosynthesis protein MoeA
HP0171_Peptide chain release factor 2 PrfB
HP0170_Hypothetical protein HP0170
HP0169_Collagenase PrtC
HP0167_Hypothetical protein HP0167
HP0166_Response regulator ArsR
HP0163_Delta−aminolevulinic acid dehydratase
HP0162_Probable transcriptional regulatory protein HP0162
HP0160_Beta lactamase HcpD
HP0159_Lipopolysaccharide 1−2−glucosyltransferase
HP0158_N−linked glycosylation glycosyltransferase
HP0157_Shikimate kinase
HP0156_Hypothetical protein HP0156
HP0155_H+/Na+ translocating NADH Dehydrogenase
HP0154_Enolase Eno
HP0153_Recombinase A RecA
HP0152_Succinyl−CoA ligase [ADP−forming] subunit alpha
HP0151_Putative membrane protein HP0151
HP0150_Hypothetical protein HP0150
HP0149_Hypothetical protein HP0149
HP0148_Hypothetical protein HP0148
HP0147_Cytochrome c oxidase− cbb3−type− subunit III FixP
HP0146_Cytochrome c oxidase− cbb3−type− FixQ
HP0145_Cbb3−type cytochrome c oxidase subunit II FixO
HP0144_Cbb3−type cytochrome c oxidase subunit I FixN
HP0142_A/G−specific adenine glycosylase MutY
HP0141_L−lactate permease LctP2
HP0140_L−lactate permease LctP1
HP0139_(S)−2−hydroxy−acid oxidase
HP0138_Iron−sulfur cluster binding protein
HP0137_Hypothetical protein HP0137
HP0136_Bacterioferritin comigratory protein
HP0135_Hypothetical protein HP0135
HP0134_3−deoxy−7−phosphoheptulonate synthase
HP0133_Serine transporter SdaC
HP0132_L−serine ammonia−lyase SdaA− SdaB
HP0130_Hypothetical protein HP0130
HP0129_Hypothetical protein HP0129
HP0127_Outer membrane protein HorB
HP0126_50S ribosomal protein L20
HP0125_50S ribosomal protein L35
HP0124_Translation initiation factor IF−3
HP0123_Threonyl−tRNA synthetase ThrS
HP0121_Phosphoenolpyruvate synthase
HP0117_Radical SAM domain−containing protein
HP0116_DNA topoisomerase I TopA
HP0115_Flagellin B FlaB
HP0114_Motility accessory factor
HP0112_L−fuculose−1−phosphate aldolase FucA
HP0111_Heat−inducible transcription repressor HrcA
HP0110_Heat shock protein GrpE
HP0109_Molecular chaperone DnaK
HP0108_Predicted coding region HP0108
HP0106_Cystathionine gamma−synthase
HP0105_S−ribosylhomocysteine lyase
HP0104_2'−3'−cyclic−nucleotide 2'−phosphodiesterase
HP0103_Methyl−accepting chemotaxis protein TlpB
HP0102_Glycosyl transferase
HP0101_Putative membrane protein HP0101
HP0100_Hypothetical protein HP0100
HP0099_Methyl−accepting chemotaxis protein TlpA
HP0098_Threonine synthase ThrC
HP0097_Hypothetical protein HP0097
HP0096_2−hydroxyacid dehydrogenase
HP0095_Hypothetical protein HP0095
HP0092_Type II restriction enzyme M protein HsdM
HP0091_Type II restriction enzyme R protein HsdR
HP0090_Malonyl CoA−acyl carrier protein transacylase
HP0089_5'−methylthioadenosine/S−adenosylhomocysteine nucleosidase
HP0088_RNA polymerase sigma factor RpoD
HP0087_Putative lipoprotein
HP0086_Quinone oxidoreductase
HP0085_Hypothetical protein HP0085
HP0084_50S ribosomal protein L13
HP0083_30S ribosomal protein S9
HP0082_Methyl−accepting chemotaxis protein TlpC
HP0080_Hypothetical protein HP0080
HP0079_Outer membrane protein HorA
HP0077_Peptide chain release factor 1
HP0076_30S ribosomal protein S20
HP0075_Phosphoglucosamine mutase
HP0074_Lipoprotein signal peptidase
HP0073_Urease subunit alpha
HP0072_Urease subunit beta
HP0071_Urease accessory protein UreI
HP0070_Urease accessory protein UreE
HP0069_Urease accessory protein UreF
HP0068_Urease accessory protein UreG
HP0067_Urease accessory protein UreH
HP0066_ATP−binding protein
HP0064_Cell division−related protein
HP0063_Hypothetical protein HP0063
HP0062_Hypothetical protein HP0062
HP0061_Hypothetical protein HP0061
HP0060_Hypothetical protein HP0060
HP0059_Hypothetical protein HP0059
HP0057_Hypothetical protein HP0057
HP0056_Delta−1−pyrroline−5−carboxylate dehydrogenase PutA
HP0055_Sodium/proline symporter PutP
HP0054_Cytosine−specific methyltransferase
HP0051_Cytosine specific DNA methyltransferase
HP0050_Adenine specific DNA methyltransferase
HP0049_Agmatine deiminase
HP0048_Transcriptional regulator HypF
HP0047_Hydrogenase expression/formation protein HypE
HP0045_Nodulation protein (nolK)
HP0044_GDP−D−mannose dehydratase
HP0043_Mannose−6−phosphate isomerase
HP0041_ComB10 competence protein
HP0039_ComB9 competence protein
HP0038_ComB8 competence protein
HP0037_NADH−ubiquinone oxidoreductase subunit
HP0036_Hypothetical protein HP0036
HP0035_Transcriptional regulatory protein
HP0034_Aspartate alpha−decarboxylase
HP0033_ATP−dependent C1p protease ClpA
HP0032_ATP−dependent Clp protease adapter protein ClpS
HP0030_Hypothetical protein HP0030
HP0028_Hypothetical protein HP0028
HP0027_Isocitrate dehydrogenase
HP0026_Type II citrate synthase
HP0025_Outer membrane protein HopD
HP0022_Lipid A phosphoethanolamine transferase
HP0021_Lipid A 1−phosphatase
HP0020_Carboxynorspermidine decarboxylase
HP0019_Chemotaxis protein CheV
HP0018_Hypothetical protein HP0018
HP0017_ComB4 competence protein
HP0016_ComB3 competence protein
HP0015_ComB2 competence protein
HP0014_Valyl−tRNA synthetase
HP0013_tRNA (5−methylaminomethyl−2−thiouridylate)−methyltransferase
HP0011_Co−chaperonin GroES

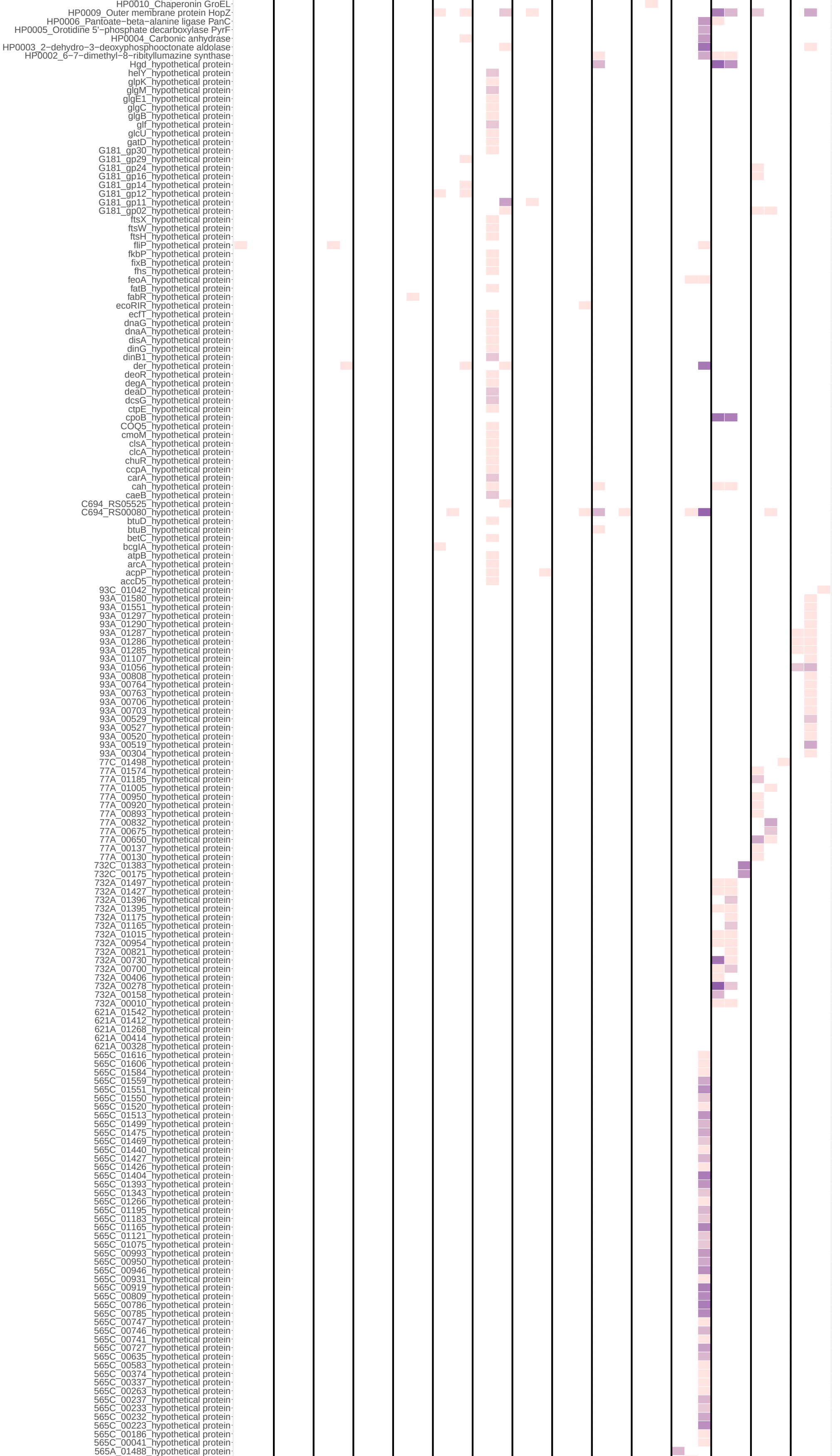
HP0010_Chaperonin GroEL
HP0009_Outer membrane protein HopZ
HP0006_Pantoate−beta−alanine ligase PanC
HP0005_Orotidine 5'−phosphate decarboxylase PyrF
HP0004_Carbonic anhydrase
HP0003_2−dehydro−3−deoxyphosphooctonate aldolase
HP0002_6−7−dimethyl−8−ribityllumazine synthase
Hgd_hypothetical protein
helY_hypothetical protein
glpK_hypothetical protein
glgM_hypothetical protein
glgE1_hypothetical protein
glgC_hypothetical protein
glgB_hypothetical protein
glf_hypothetical protein
glcU_hypothetical protein
gatD_hypothetical protein
G181_gp30_hypothetical protein
G181_gp29_hypothetical protein
G181_gp24_hypothetical protein
G181_gp16_hypothetical protein
G181_gp14_hypothetical protein
G181_gp12_hypothetical protein
G181_gp11_hypothetical protein
G181_gp02_hypothetical protein
ftsX_hypothetical protein
ftsW_hypothetical protein
ftsH_hypothetical protein
fliP_hypothetical protein
fkbP_hypothetical protein
fixB_hypothetical protein
fhs_hypothetical protein
feoA_hypothetical protein
fatB_hypothetical protein
fabR_hypothetical protein
ecoRIR_hypothetical protein
ecfT_hypothetical protein
dnaG_hypothetical protein
dnaA_hypothetical protein
disA_hypothetical protein
dinG_hypothetical protein
dinB1_hypothetical protein
der_hypothetical protein
deoR_hypothetical protein
degA_hypothetical protein
deaD_hypothetical protein
dcsG_hypothetical protein
ctpE_hypothetical protein
cpoB_hypothetical protein
COQ5_hypothetical protein
cmoM_hypothetical protein
clsA_hypothetical protein
clcA_hypothetical protein
chuR_hypothetical protein
ccpA_hypothetical protein
carA_hypothetical protein
cah_hypothetical protein
caeB_hypothetical protein
C694_RS05525_hypothetical protein
C694_RS00080_hypothetical protein
btuD_hypothetical protein
btuB_hypothetical protein
betC_hypothetical protein
bcgIA_hypothetical protein
atpB_hypothetical protein
arcA_hypothetical protein
acpP_hypothetical protein
accD5_hypothetical protein
93C_01042_hypothetical protein
93A_01580_hypothetical protein
93A_01551_hypothetical protein
93A_01297_hypothetical protein
93A_01290_hypothetical protein
93A_01287_hypothetical protein
93A_01286_hypothetical protein
93A_01285_hypothetical protein
93A_01107_hypothetical protein
93A_01056_hypothetical protein
93A_00808_hypothetical protein
93A_00764_hypothetical protein
93A_00763_hypothetical protein
93A_00706_hypothetical protein
93A_00703_hypothetical protein
93A_00529_hypothetical protein
93A_00527_hypothetical protein
93A_00520_hypothetical protein
93A_00519_hypothetical protein
93A_00304_hypothetical protein
77C_01498_hypothetical protein
77A_01574_hypothetical protein
77A_01185_hypothetical protein
77A_01005_hypothetical protein
77A_00950_hypothetical protein
77A_00920_hypothetical protein
77A_00893_hypothetical protein
77A_00832_hypothetical protein
77A_00675_hypothetical protein
77A_00650_hypothetical protein
77A_00137_hypothetical protein
77A_00130_hypothetical protein
732C_01383_hypothetical protein
732C_00175_hypothetical protein
732A_01497_hypothetical protein
732A_01427_hypothetical protein
732A_01396_hypothetical protein
732A_01395_hypothetical protein
732A_01175_hypothetical protein
732A_01165_hypothetical protein
732A_01015_hypothetical protein
732A_00954_hypothetical protein
732A_00821_hypothetical protein
732A_00730_hypothetical protein
732A_00700_hypothetical protein
732A_00406_hypothetical protein
732A_00278_hypothetical protein
732A_00158_hypothetical protein
732A_00010_hypothetical protein
621A_01542_hypothetical protein
621A_01412_hypothetical protein
621A_01268_hypothetical protein
621A_00414_hypothetical protein
621A_00328_hypothetical protein
565C_01616_hypothetical protein
565C_01606_hypothetical protein
565C_01584_hypothetical protein
565C_01559_hypothetical protein
565C_01551_hypothetical protein
565C_01550_hypothetical protein
565C_01520_hypothetical protein
565C_01513_hypothetical protein
565C_01499_hypothetical protein
565C_01475_hypothetical protein
565C_01469_hypothetical protein
565C_01440_hypothetical protein
565C_01427_hypothetical protein
565C_01426_hypothetical protein
565C_01404_hypothetical protein
565C_01393_hypothetical protein
565C_01343_hypothetical protein
565C_01266_hypothetical protein
565C_01195_hypothetical protein
565C_01183_hypothetical protein
565C_01165_hypothetical protein
565C_01121_hypothetical protein
565C_01075_hypothetical protein
565C_00993_hypothetical protein
565C_00950_hypothetical protein
565C_00946_hypothetical protein
565C_00931_hypothetical protein
565C_00919_hypothetical protein
565C_00809_hypothetical protein
565C_00786_hypothetical protein
565C_00785_hypothetical protein
565C_00747_hypothetical protein
565C_00746_hypothetical protein
565C_00741_hypothetical protein
565C_00727_hypothetical protein
565C_00635_hypothetical protein
565C_00583_hypothetical protein
565C_00374_hypothetical protein
565C_00337_hypothetical protein
565C_00263_hypothetical protein
565C_00237_hypothetical protein
565C_00233_hypothetical protein
565C_00232_hypothetical protein
565C_00223_hypothetical protein
565C_00186_hypothetical protein
565C_00041_hypothetical protein
565A_01488_hypothetical protein

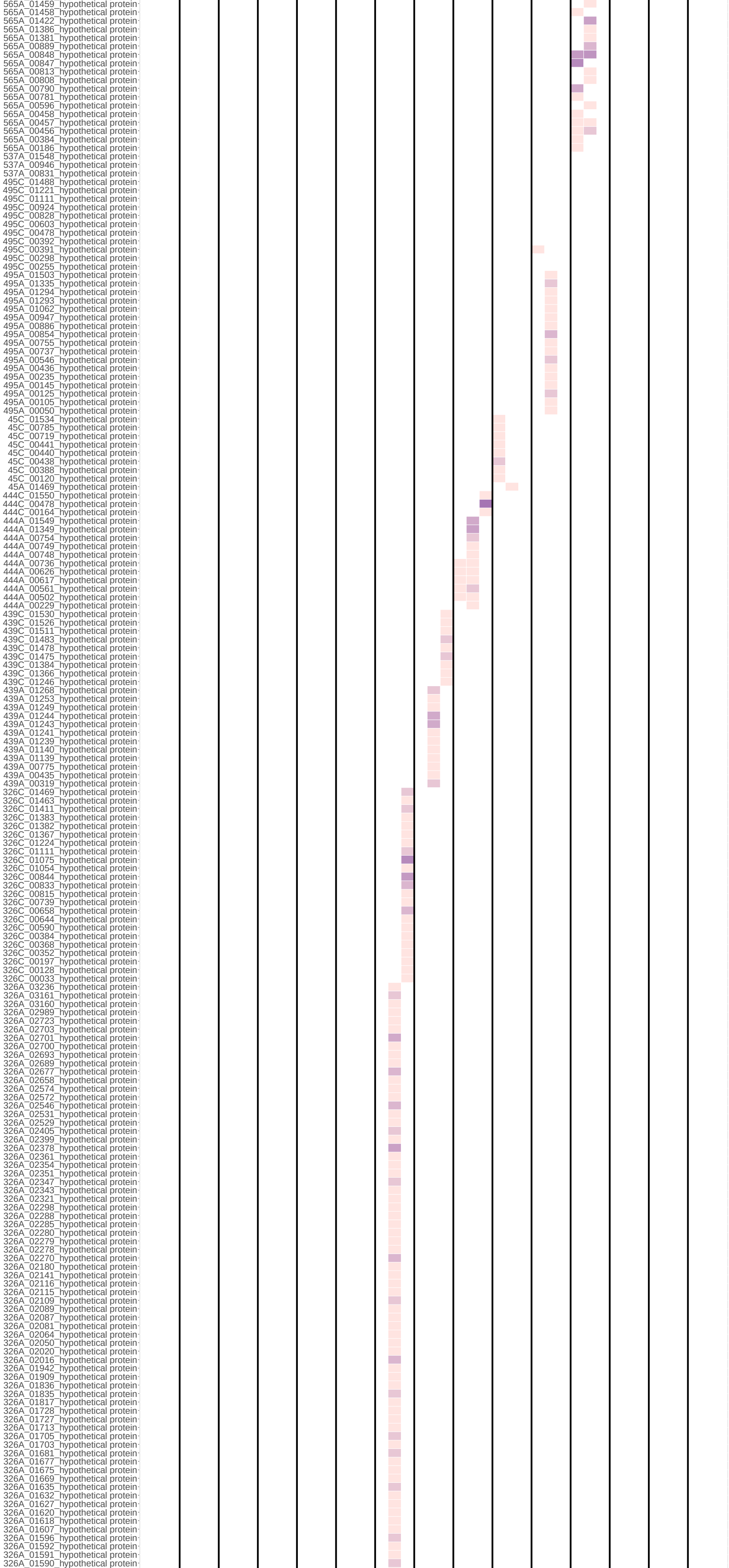
565A_01459_hypothetical protein
565A_01458_hypothetical protein
565A_01422_hypothetical protein
565A_01386_hypothetical protein
565A_01381_hypothetical protein
565A_00889_hypothetical protein
565A_00848_hypothetical protein
565A_00847_hypothetical protein
565A_00813_hypothetical protein
565A_00808_hypothetical protein
565A_00790_hypothetical protein
565A_00781_hypothetical protein
565A_00596_hypothetical protein
565A_00458_hypothetical protein
565A_00457_hypothetical protein
565A_00456_hypothetical protein
565A_00384_hypothetical protein
565A_00186_hypothetical protein
537A_01548_hypothetical protein
537A_00946_hypothetical protein
537A_00831_hypothetical protein
495C_01488_hypothetical protein
495C_01221_hypothetical protein
495C_01111_hypothetical protein
495C_00924_hypothetical protein
495C_00828_hypothetical protein
495C_00603_hypothetical protein
495C_00478_hypothetical protein
495C_00392_hypothetical protein
495C_00391_hypothetical protein
495C_00298_hypothetical protein
495C_00255_hypothetical protein
495A_01503_hypothetical protein
495A_01335_hypothetical protein
495A_01294_hypothetical protein
495A_01293_hypothetical protein
495A_01062_hypothetical protein
495A_00947_hypothetical protein
495A_00886_hypothetical protein
495A_00854_hypothetical protein
495A_00755_hypothetical protein
495A_00737_hypothetical protein
495A_00546_hypothetical protein
495A_00436_hypothetical protein
495A_00235_hypothetical protein
495A_00145_hypothetical protein
495A_00125_hypothetical protein
495A_00105_hypothetical protein
495A_00050_hypothetical protein
45C_01534_hypothetical protein
45C_00785_hypothetical protein
45C_00719_hypothetical protein
45C_00441_hypothetical protein
45C_00440_hypothetical protein
45C_00438_hypothetical protein
45C_00388_hypothetical protein
45C_00120_hypothetical protein
45A_01469_hypothetical protein
444C_01550_hypothetical protein
444C_00478_hypothetical protein
444C_00164_hypothetical protein
444A_01549_hypothetical protein
444A_01349_hypothetical protein
444A_00754_hypothetical protein
444A_00749_hypothetical protein
444A_00748_hypothetical protein
444A_00736_hypothetical protein
444A_00626_hypothetical protein
444A_00617_hypothetical protein
444A_00561_hypothetical protein
444A_00502_hypothetical protein
444A_00229_hypothetical protein
439C_01530_hypothetical protein
439C_01526_hypothetical protein
439C_01511_hypothetical protein
439C_01483_hypothetical protein
439C_01478_hypothetical protein
439C_01475_hypothetical protein
439C_01384_hypothetical protein
439C_01366_hypothetical protein
439C_01246_hypothetical protein
439A_01268_hypothetical protein
439A_01253_hypothetical protein
439A_01249_hypothetical protein
439A_01244_hypothetical protein
439A_01243_hypothetical protein
439A_01241_hypothetical protein
439A_01239_hypothetical protein
439A_01140_hypothetical protein
439A_01139_hypothetical protein
439A_00775_hypothetical protein
439A_00435_hypothetical protein
439A_00319_hypothetical protein
326C_01469_hypothetical protein
326C_01463_hypothetical protein
326C_01411_hypothetical protein
326C_01383_hypothetical protein
326C_01382_hypothetical protein
326C_01367_hypothetical protein
326C_01224_hypothetical protein
326C_01111_hypothetical protein
326C_01075_hypothetical protein
326C_01054_hypothetical protein
326C_00844_hypothetical protein
326C_00833_hypothetical protein
326C_00815_hypothetical protein
326C_00739_hypothetical protein
326C_00658_hypothetical protein
326C_00644_hypothetical protein
326C_00590_hypothetical protein
326C_00384_hypothetical protein
326C_00368_hypothetical protein
326C_00352_hypothetical protein
326C_00197_hypothetical protein
326C_00128_hypothetical protein
326C_00033_hypothetical protein
326A_03236_hypothetical protein
326A_03161_hypothetical protein
326A_03160_hypothetical protein
326A_02989_hypothetical protein
326A_02723_hypothetical protein
326A_02703_hypothetical protein
326A_02701_hypothetical protein
326A_02700_hypothetical protein
326A_02693_hypothetical protein
326A_02689_hypothetical protein
326A_02677_hypothetical protein
326A_02658_hypothetical protein
326A_02574_hypothetical protein
326A_02572_hypothetical protein
326A_02546_hypothetical protein
326A_02531_hypothetical protein
326A_02529_hypothetical protein
326A_02405_hypothetical protein
326A_02399_hypothetical protein
326A_02378_hypothetical protein
326A_02361_hypothetical protein
326A_02354_hypothetical protein
326A_02351_hypothetical protein
326A_02347_hypothetical protein
326A_02343_hypothetical protein
326A_02321_hypothetical protein
326A_02298_hypothetical protein
326A_02288_hypothetical protein
326A_02285_hypothetical protein
326A_02280_hypothetical protein
326A_02279_hypothetical protein
326A_02278_hypothetical protein
326A_02270_hypothetical protein
326A_02180_hypothetical protein
326A_02141_hypothetical protein
326A_02116_hypothetical protein
326A_02115_hypothetical protein
326A_02109_hypothetical protein
326A_02089_hypothetical protein
326A_02087_hypothetical protein
326A_02081_hypothetical protein
326A_02064_hypothetical protein
326A_02050_hypothetical protein
326A_02020_hypothetical protein
326A_02016_hypothetical protein
326A_01942_hypothetical protein
326A_01909_hypothetical protein
326A_01836_hypothetical protein
326A_01835_hypothetical protein
326A_01817_hypothetical protein
326A_01728_hypothetical protein
326A_01727_hypothetical protein
326A_01713_hypothetical protein
326A_01705_hypothetical protein
326A_01703_hypothetical protein
326A_01681_hypothetical protein
326A_01677_hypothetical protein
326A_01675_hypothetical protein
326A_01669_hypothetical protein
326A_01635_hypothetical protein
326A_01632_hypothetical protein
326A_01627_hypothetical protein
326A_01620_hypothetical protein
326A_01618_hypothetical protein
326A_01607_hypothetical protein
326A_01596_hypothetical protein
326A_01592_hypothetical protein
326A_01591_hypothetical protein
326A_01590_hypothetical protein

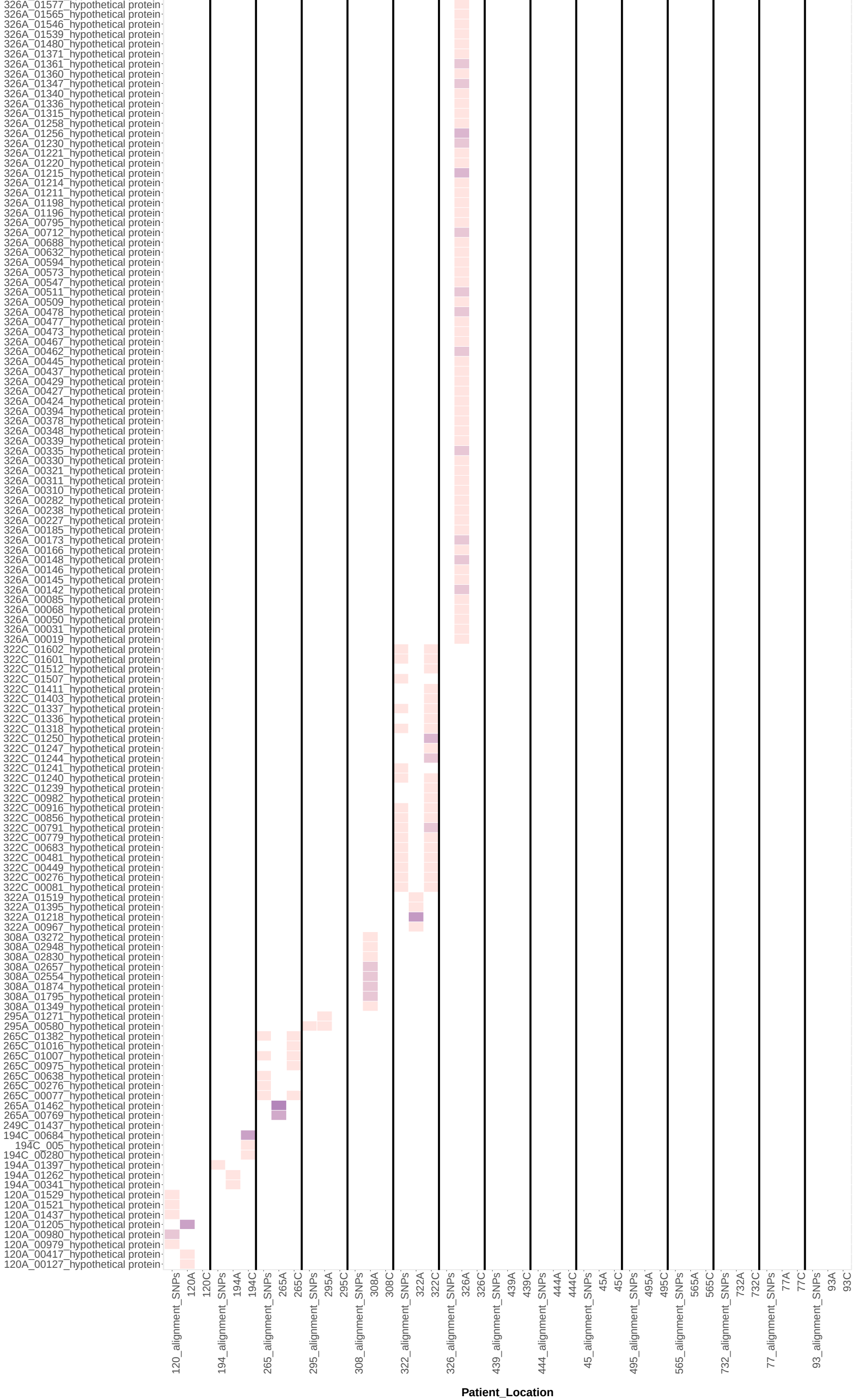
326A_01577_hypothetical protein
326A_01565_hypothetical protein
326A_01546_hypothetical protein
326A_01539_hypothetical protein
326A_01480_hypothetical protein
326A_01371_hypothetical protein
326A_01361_hypothetical protein
326A_01360_hypothetical protein
326A_01347_hypothetical protein
326A_01340_hypothetical protein
326A_01336_hypothetical protein
326A_01315_hypothetical protein
326A_01258_hypothetical protein
326A_01256_hypothetical protein
326A_01230_hypothetical protein
326A_01221_hypothetical protein
326A_01220_hypothetical protein
326A_01215_hypothetical protein
326A_01214_hypothetical protein
326A_01211_hypothetical protein
326A_01198_hypothetical protein
326A_01196_hypothetical protein
326A_00795_hypothetical protein
326A_00712_hypothetical protein
326A_00688_hypothetical protein
326A_00632_hypothetical protein
326A_00594_hypothetical protein
326A_00573_hypothetical protein
326A_00547_hypothetical protein
326A_00511_hypothetical protein
326A_00509_hypothetical protein
326A_00478_hypothetical protein
326A_00477_hypothetical protein
326A_00473_hypothetical protein
326A_00467_hypothetical protein
326A_00462_hypothetical protein
326A_00445_hypothetical protein
326A_00437_hypothetical protein
326A_00429_hypothetical protein
326A_00427_hypothetical protein
326A_00424_hypothetical protein
326A_00394_hypothetical protein
326A_00378_hypothetical protein
326A_00348_hypothetical protein
326A_00339_hypothetical protein
326A_00335_hypothetical protein
326A_00330_hypothetical protein
326A_00321_hypothetical protein
326A_00311_hypothetical protein
326A_00310_hypothetical protein
326A_00282_hypothetical protein
326A_00238_hypothetical protein
326A_00227_hypothetical protein
326A_00185_hypothetical protein
326A_00173_hypothetical protein
326A_00166_hypothetical protein
326A_00148_hypothetical protein
326A_00146_hypothetical protein
326A_00145_hypothetical protein
326A_00142_hypothetical protein
326A_00085_hypothetical protein
326A_00068_hypothetical protein
326A_00050_hypothetical protein
326A_00031_hypothetical protein
326A_00019_hypothetical protein
322C_01602_hypothetical protein
322C_01601_hypothetical protein
322C_01512_hypothetical protein
322C_01507_hypothetical protein
322C_01411_hypothetical protein
322C_01403_hypothetical protein
322C_01337_hypothetical protein
322C_01336_hypothetical protein
322C_01318_hypothetical protein
322C_01250_hypothetical protein
322C_01247_hypothetical protein
322C_01244_hypothetical protein
322C_01241_hypothetical protein
322C_01240_hypothetical protein
322C_01239_hypothetical protein
322C_00982_hypothetical protein
322C_00916_hypothetical protein
322C_00856_hypothetical protein
322C_00791_hypothetical protein
322C_00779_hypothetical protein
322C_00683_hypothetical protein
322C_00481_hypothetical protein
322C_00449_hypothetical protein
322C_00276_hypothetical protein
322C_00081_hypothetical protein
322A_01519_hypothetical protein
322A_01395_hypothetical protein
322A_01218_hypothetical protein
322A_00967_hypothetical protein
308A_03272_hypothetical protein
308A_02948_hypothetical protein
308A_02830_hypothetical protein
308A_02657_hypothetical protein
308A_02554_hypothetical protein
308A_01874_hypothetical protein
308A_01795_hypothetical protein
308A_01349_hypothetical protein
295A_01271_hypothetical protein
295A_00580_hypothetical protein
265C_01382_hypothetical protein
265C_01016_hypothetical protein
265C_01007_hypothetical protein
265C_00975_hypothetical protein
265C_00638_hypothetical protein
265C_00276_hypothetical protein
265C_00077_hypothetical protein
265A_01462_hypothetical protein
265A_00769_hypothetical protein
249C_01437_hypothetical protein
194C_00684_hypothetical protein
194C_005_hypothetical protein
194C_00280_hypothetical protein
194A_01397_hypothetical protein
194A_01262_hypothetical protein
194A_00341_hypothetical protein
120A_01529_hypothetical protein
120A_01521_hypothetical protein
120A_01437_hypothetical protein
120A_01205_hypothetical protein
120A_00980_hypothetical protein
120A_00979_hypothetical protein
120A_00417_hypothetical protein
120A_00127_hypothetical protein
120_alignment_SNPs
120A
120C
194_alignment_SNPs
194A
194C
265_alignment_SNPs
265A
265C
295_alignment_SNPs
295A
295C
308_alignment_SNPs
308A
308C
322_alignment_SNPs
322A
322C
326_alignment_SNPs
326A
326C
439_alignment_SNPs
439A
439C
444_alignment_SNPs
444A
444C
45_alignment_SNPs
45A
45C
495_alignment_SNPs
495A
495C
565_alignment_SNPs
565A
565C
732_alignment_SNPs
732A
732C
77_alignment_SNPs
77A
77C
93_alignment_SNPs
93A
93C
Patient_Location
